# Genome-Wide Identification and Functional Characterization of the Chloride Channel TaCLC Gene Family in Wheat (*Triticum aestivum* L.)

**DOI:** 10.3389/fgene.2022.846795

**Published:** 2022-03-16

**Authors:** Peijun Mao, Yonghang Run, Hanghui Wang, Changdong Han, Lijun Zhang, Kehui Zhan, Haixia Xu, Xiyong Cheng

**Affiliations:** Co-construction State Key Laboratory of Wheat and Maize Crop Science, Collaborative Innovation Center of Henan Grain Crops, College of Agronomy, Henan Agricultural University, Zhengzhou, China

**Keywords:** wheat (*Triticum aestivum* L.), CLC, gene family, nitrate nitrogen, functional characterization

## Abstract

In plants, chloride channels (CLC) are involved in a series of specific functions, such as regulation of nutrient transport and stress tolerance. Members of the wheat *Triticum aestivum* L. CLC (TaCLC) gene family have been proposed to encode anion channels/transporters that may be related to nitrogen transportation. To better understand their roles, TaCLC family was screened and 23 *TaCLC* gene sequences were identified using a Hidden Markov Model in conjunction with wheat genome database. Gene structure, chromosome location, conserved motif, and expression pattern of the resulting family members were then analyzed. Phylogenetic analysis showed that the TaCLC family can be divided into two subclasses (I and II) and seven clusters (-a, -c1, -c2, -e, -f1, -f2, and -g2). Using a wheat RNA-seq database, the expression pattern of TaCLC family members was determined to be an inducible expression type. In addition, seven genes from seven different clusters were selected for quantitative real-time PCR (qRT-PCR) analysis under low nitrogen stress or salt stress conditions, respectively. The results indicated that the gene expression levels of this family were up-regulated under low nitrogen stress and salt stress, except the genes of TaCLC-c2 cluster which were from subfamily -c. The yeast complementary experiments illustrated that *TaCLC-a-6AS-1*, *TaCLC-c1-3AS*, and *TaCLC-e-3AL* all had anion transport functions for NO_3_^−^ or Cl^−^, and compensated the hypersensitivity of yeast GEF1 mutant strain YJR040w (*Δgef1*) in restoring anion-sensitive phenotype. This study establishes a theoretical foundation for further functional characterization of *TaCLC* genes and provides an initial reference for better understanding nitrate nitrogen transportation in wheat.

## Introduction

Nitrogen is the most important nutrient element for plant growth and development. The composition of more complex compounds such as proteins, nucleic acids and enzymes, which are crucial for plant functioning, is inseparable from nitrogen itself (Lv et al., 2021). Despite its benefits, excessive nitrogen application brings serious negative effects, including ground water nitrate pollution and eutrophication of rivers and lakes. According to available statistical data, 25 million tons of nitrogen fertilizer are applied annually in China, three times the world average. However, the utilization efficiency of nitrogen fertilizer has only reached 30% of the applied amount (Ju and Zhang, 2017). Given this, improving nitrogen use efficiency is of great significance to crops, as better efficiency will reduce nitrogen fertilizer pollution in surrounding ecosystems.

The main forms of nitrogen absorbed and used by most plants are nitrate nitrogen (NO_3_^−^) and ammonium nitrogen (NH_4_^+^). In plants, ammonium nitrogen absorption is mainly regulated by the ammonium transporter (AMT) genes (Li et al., 2017). Conversely, nitrate nitrogen uptake is mainly regulated by four types of NO_3_^−^ transporters: low affinity nitrate transporter NRT1, high affinity nitrate transporter NRT2, chloride channel protein CLC, and slow anion channel related homologue SLAC1/SLAH (Liu R. et al., 2020). Of these, the chloride channel (CLC) gene family is widely distributed across a variety of archaea, microbial fungi, mammals, and plants (Park et al., 2017). The members of the CLC family were first discovered in *Torpedo California* (electric ray fish) by White and Miller in 1979 (White and Miller, 1979). The first family gene was isolated from marine ray (*Torpedo marmorata*) by Jentsch in 1990 and named *CLC-0* (Jentsch et al., 1990). Later, it was discovered that the *CLC* family genes also existed in most plants, including *Arabidopsis thaliana*, *Nicotiana tabacum*, *Oryza sativa* (L.), *Poncirus trifoliata* (L.) Raf., *Zea mays* (L.), and *Glycine max* (Lurin et al., 1996; Diedhiou and Golldack, 2006; Lv et al., 2009; Wang et al., 2015; Wei et al., 2015). Plants CLC proteins play important roles in turgor maintenance, stomatal movement, ion homeostasis, as well as enhancing drought and salt tolerance, and increasing nitrate accumulation (Wei et al., 2015; Zhang et al., 2018; Liu C. et al., 2020).

Structurally, the identified *CLC* genes all have a highly conserved voltage-gated chloride channel (Voltage-gate CLC) domain and two conserved Cystathionine beta synthase (CBS) domains (Xing et al., 2020). At present, the function and classification of Arabidopsis *CLC* family genes have been extensively and deeply studied. Specifically, members of the AtCLC gene family have been divided into two subclasses (I and II) and 7 subfamilies (-a, -b, -c, -d, -e, -f, and -g) (Nedelyaeva et al., 2020). Subclass Ⅰ is mainly composed of AtCLC-a/-b/-c/-d/-g subfamily, while subclass II is composed of AtCLC-e/-f subfamily (Nedelyaeva et al., 2020). In addition, it was found that subclass I contained the patterns GxGIPE (I), GKxGPxxH (II), and PxxGxLF (III). Comparatively, subclass II did not contain any of the above conserved motifs. When x in the conserved region of the gene GxGIPE (I) is a proline (P, Pro) residue, NO_3_^−^ is preferentially transported. However, when x is a serine (S, Ser) residue, Cl^−^ is preferentially transported. Past work has also shown that when the x in the conserved region (II) is a conservative-gated glutamate (E, Glu) and the fourth residue in the conserved region (III) is proton glutamate (E, Glu) residue, the protein function is a CLC antiporter rather than a CLC channel (Subba et al., 2021). The *CLC* family genes are not only comprised of channel proteins for anions such as chloride ion and nitrate ion, but also plays vital functional roles in regulating stomatal movement, maintaining both the potential balance and proton gradient in plant cell, transporting and accumulating nutrients in plants (Zifarelli and Pusch, 2010). In Arabidopsis, the *AtCLC-a* gene is located on the vacuolar membrane of plant vacuole and functions as a NO_3_^−^/H^+^ exchanger (De Angeli et al., 2006; Wege et al., 2014). The C-terminus of *AtCLC-a* can be combined with ATP and nitrate/proton alkynol to regulate the specific accumulation of nitrate in the vacuole (De Angeli et al., 2009). *AtCLC-b* is located on the vacuolar membrane as the second vacuolar NO_3_^−^/H^+^ exchanger (Whiteman et al., 2008; von der Fecht-Bartenbach et al., 2010). *AtCLC-c* is involved in stomatal movement and associated with salt tolerance (Harada et al., 2004; Jossier et al., 2010). *AtCLC-d* mediates the transport of anions such as Cl^−^ or NO_3_^−^, and regulates the luminal pH of the Golgi network (von der Fecht-Bartenbach et al., 2007). *AtCLC-e* and *AtCLC-f* are related to the thylakoid and Golgi membranes, respectively (Marmagne et al., 2007). *AtCLC-a*, *AtCLC-b*, and *AtCLC-d* play important roles in regulating root elongation (Moradi et al., 2015). *OsCLC1* improves rice drought tolerance and increases yield (Um et al., 2018). The overexpression of the maize gene *ZmCLC-d* in *Arabidopsis thaliana* allows for better tolerance to cold, drought and salt stresses by an increased germination rate, root length, plant survival rate, antioxidant enzyme activities, and a reduced accumulation of Cl^−^ in transgenic plants (Wang et al., 2015).

Wheat (*Triticum aestivum* L.) serves as the staple food for 30% of global population, and is an important cereal crop with a high demand for nitrogen fertilizer to enable the grain protein accumulation (Zörb et al., 2018). As a preferred nitrate crop, wheat mainly absorbs nitrate nitrogen as its nitrogen source. Studies showed that wheat biomass under conditions of either single ammonium nitrogen or single nitrate nitrogen was lower than when combined ammonium nitrogen and nitrate nitrogen were used (Ijato et al., 2021). Single ammonium nitrogen has a toxic effect on wheat, while nitrate nitrogen alleviates part of this toxic effect (Wang et al., 2016). The *CLC* genes have been shown to be related to the transportation of nitrate. However, the functional roles of the *Triticum aestivum* L. *CLC* (*TaCLC*) genes remain less well known. In this study, 23 wheat *TaCLC* genes were identified by a genome-wide search using released wheat genome data. We analyzed the phylogeny as well as conserved motifs in TaCLC proteins, gene structures, and expression patterns under stress condition. In addition, the part of *TaCLC* genes were functionally characterized in yeast mutant. Our study will lay a preliminary foundation for future research into the functions of *TaCLCs* gene.

## Materials and Methods

### Identification of *Triticum aestivum* L. CLC Gene Family Members in Wheat

Wheat genome (*Triticum_aestivum*.IWGSC.dna.toplevel.fa, 2021), GFF3 file (*Triticum_aestivum*.IWGSC.49.gff3, 2021), and protein sequences (*Triticum_aestivum*.IWGSC.pep.all.fa, 2021) were downloaded from the ensembl plant website (http://plants.ensembl.org/index.html).

The Pfam protein family database (http://pfam.xfam.org/) was used to search the ID number of the CLC gene family and its distribution across species (Finn et al., 2006). The Pfam accession number ID PF00654 was then used to search the sequences with default parameter settings in the Emsembl Plant database (http://plants.ensembl.org/index.html, *Triticum aestivum* IWGSCv1.1) (Cheng et al., 2018). The Blastp program of TBtools software was used to compare the amino acid sequence of wheat genome (http://ftp.ensemblgenomes.org/pub/plants/release-49/fasta/triticum_aestivum/pep/) with the protein sequence of *AtCLC* genes (Supplementary Table S1) and *AtCLC* used as the reference sequence (Chen et al., 2020). Then the *TaCLC* candidate genes obtained by HMM search and Blastp were compared. The ID of the same gene obtained by search retained only the longest transcript sequence. Incomplete gene sequences without either the initial or termination codon and mutation sequences were removed. The remaining sequences were detected using the CDD tool in NCBI (https://www.ncbi.nlm.nih.gov/) and any sequences with incomplete voltage-gated CLC domains were discarded. The final genes were members of the wheat TaCLC gene family. The gene length, encoded amino acid length, intron number, exon number and other biological information were downloaded from the Emsembl Plant database. The TaCLC sequence of wheat was submitted to blast online program in Ensembl Plant to find homologous genes in *Arabidopsis thaliana genome* (TAIR10.1). The GFF annotation information of *TaCLCs* gene was downloaded from the Emsembl Plant database. Subcellular localization of each *TaCLC* gene was predicted using WolfPSORT (https://wolfpsort.hgc.jp/) online tools. The gene structure was analyzed using TBtools software. We identified 33 conserved motifs of TaCLC based on the HMM logo in the Pfam database. The conserved motifs of TaCLC protein were identified by MEME (http://meme-suite.org/). The number of motifs setting was 33 and the motif length was set to 2–200 aa. Default parameters were used for all the programmes unless otherwise stated.

### Phylogenetic Analysis of *Triticum aestivum* L. CLC Proteins

The protein sequences of AtCLC, OsCLC and GmCLC were retrieved from NCBI (https://www.ncbi.nlm.nih.gov/), RiceDate (https://www.ricedata.cn/), and SoyBase (https://www.soybase.org/), respectively. All these protein sequences are listed in Supplementary Table S1. The Clustal W tool in MAGA7.0 software was used to compare the reported CLC protein sequences of *Arabidopsis thaliana* (At), *Oryza sativa* L. (Os), *Glycine max* (Gm) and the newly identified wheat TaCLC protein sequences (Kumar et al., 2016). A phylogenetic tree was constructed using the neighbor-joining (NJ) method with the bootstrap replicates setting parameter of 1000.

### Physical Location on Chromosomes, Collinearity Analysis, and Transmembrane Structure Prediction of Encoding Protein

The physical, chromosomal position of each *TaCLC* gene was obtained from the IWGSC RefSeq V1.0 database (https://urgi.versailles.inra.fr/). The physical map was plotted using MapInspect software according to the starting position and chromosome length of each gene. One Step MCScanX program of TBtools software was used to compare and integrate the whole genome sequence and gene structure annotation information of wheat and the E-value parameter setting was 10E-05 (Chen et al., 2020). In order to further predict the interspecific evolution mechanism of TaCLC family members, we also used TBtools software to integrate and compare the whole wheat genome sequence and gene structure annotation with *Arabidopsis thaliana*, *Oryza sativa* L. and *Triticum dicoccoides*. The data were from Emsembl Plant database (http://ftp.ensemblgenomes.org/pub/plants/release-49/fasta/) and the E-value parameter setting was 10E-05. Dual Systeny Plot program and Circle Gene View of TBtools software were used to visualize the collinearity results of *TaCLC* gene sequences. PROTTER (http://wlab.ethz.ch/protter/start/) online tools were used to analyze the transmembrane structure of *TaCLCs* gene-encoded proteins.

### Expression Prediction of *Triticum aestivum* L. CLC Genes in Various Wheat Tissues

Using the wheat expression database ExpWheat (https://wheat.pw.usda.gov/WheatExp/), the expression patterns of *TaCLC* family genes in wheat plant roots, stems, leaves, panicles, grains, and other tissues were obtained. The expression of genes at different stages was also predicted. A heat map of *TaCLCs* gene expression was drawn by Heml software.

### Plant Materials, Growth Conditions, and Stress Conditions

Wheat (*Triticum aestivum* L. cv*.* Yunong 804) seeds were planted for 5 days in culture dishes containing water in a greenhouse at 22°C with a 16 h light/8 h dark photoperiod. The 5-day-old wheat seedlings were transplanted into a nutrient solution (Supplementary Table S2), which was replaced every 3 days until the seedlings reached the three-leaf stage. The plantlets were then subjected to low nitrogen (0.4 mM NH_4_NO_3_) or salt stress (100 mM NaCl) treatment. Roots and shoots were collected at 0, 2, 6, 12, and 24 h after treatment with 0.4 mM NH_4_NO_3_. Whole plantlets were collected at 0, 1, 3, 6, 9, 12, and 24 h after treatment with 100 mM NaCl. Each treatment was conducted using three independent biological replicates and samples were collected from three plants for each treatment at each replication. All plant samples were immediately collected and frozen in liquid nitrogen prior to storing at −80°C for further RNA isolation.

### Quantitative Real-Time PCR Validation

To understand the expression pattern of *TaCLC* genes under short-term stress of different environment conditions, we selected one gene from each cluster of the TaCLC gene family. In total, seven genes were screened to validate expression levels. Specific fluorescence quantitative primers (Supplementary Table S3) were designed using Primer 5 software (http://www.premierbiosoft.com/index.html). Total RNA was extracted from different treatment groups using TransZOL (TransGen Biotech, Beijing, China) according to the manufacturer’s instructions. Then, cDNA was synthesized using PrimeScript™ RT reagent Kit with gDNA Eraser (Takara, Dalian, China). Reverse transcription cDNA was used as template for amplification with Hieff UNICON® qPCR SYBR Green Master Mix (YEASEN Biotechnology, Shanghai, China) in Quantstudio™5 (Thermo Fisher, Shanghai, China). Quantitative real-time PCR (qRT-PCR) thermocycling conditions were as follows: 95°C for 5 min, followed by 40 cycles of 95°C for 10 s and 60°C for 30 s, and a final extension at 72°C for 5 min. The expression levels of six genes (TaCLC-c2 cluster genes were removed because they were not expressed and the relative expression cannot be calculated) selected from the TaCLC gene family under different stress conditions were calculated using the 2^−ΔΔct^ method. The wheat *β-actin* gene was used as an internal reference control. The average of three independent biological replicates was used for all data analysis.

### Functional Complementation Experiment in Yeast

Yeast mutant strain YJR040w (*Δgef1*; MATa; his3Δ1; leu2Δ0; met15Δ0; ura3Δ0; YJR040w: kanMX4) lacks the only CLC family protein—GEF1—in *Saccharomyces Cerevisiae* and is sensitive to low nitrogen medium. To further illustrate the function of *TaCLC* genes, we used the EUROSCARF (http://www.euroscarf.de/index.php?name=News) yeast mutant YJR040w that heterologously expressed the TaCLCs. The primer sequences of *TaCLC-a-6AS-1*, *TaCLC-c1-3AS*, and *TaCLC-e-3AL* used for gene amplification are listed in Supplementary Table S3. The selected *TaCLCs* gene was cloned into the yeast expression vector p416 by ClonExpress^®^ II One Step Cloning Kit (Vazyme Biotech Co., Ltd., Nanjing, China). The constructed recombinant plasmid was then transformed into YJR040w using the lithium acetate transformation method and uniformly plated on a SD/-Ura Broth (Coolaber Technology Co., Ltd., Beijing, China) solid plate. The monoclonal was selected and verified by PCR with p416-F/p416-R (Supplementary Table S3). The monoclonal containing the target band was transferred to liquid SD/-Ura medium and cultured to OD_600_ = 0.6. These initial yeast cultures were used to prepare a series of diluents (10^−1^). From each gradient, 5 μl samples of each diluted culture were plated into YTD medium (1% yeast extract/2% tryptone/2% dextrose), YTD medium supplemented with 1 M KCl, 1 M NaCl, or 1 M KNO_3_, respectively. The plates were incubated at 30°C for 3–5 days and photographed.

## Results

### Identification of *Triticum aestivum* L. CLC Family Genes in Wheat

We used the voltage-gated chloride channel protein conserved domain PF00654 as a search sequence in conjunction with a wheat genome database to perform alignment and to remove incomplete conserved domains. A total of 23 non-redundant *TaCLC* candidate gene sequences were identified and named based on the orthologous relationships of the rice family genes (Table 1). Information about the chromosome on which the gene was located was indicated by chromosomal arm symbols on the gene/protein name. When multiple gene sequences of the wheat TaCLC family members formed the same cluster as a certain gene as that seen in rice, a number was added after the gene name to distinguish these two genes, such as *TaCLC-c1-3DS-1* and *TaCLC-c1-3DS-2*.

**TABLE 1:** Main information of 23 wheat TaCLC genes.

Gene Name	Ensembl ID	Gene length	Amino acid length	Intron No.	Exon No.	Location		SL	TMS	Chromosome	Accession of Arabidopsis
						Start	End				
*TaCLC-a-6AS-1*	TraesCS6A02G098500.2	3,036	796	3	4	65681325	65684847	PM	11	6A	*AT3G27170 (AtCLC-b)*
*TaCLC-a-6AS-2*	TraesCS6A02G098600.1	2,813	778	3	4	65797706	65800777	PM	11	6A	*AT5G40890 (AtCLC-a)*
*TaCLC-a-6BS-1*	TraesCS6B02G126400.1	3,161	787	4	5	121811989	121815556	PM	11	6B	*AT3G27170 (AtCLC-b)*
*TaCLC-a-6BS-2*	TraesCS6B02G126800.1	2,870	784	3	4	122290496	122293648	PM	11	6B	*AT3G27170 (AtCLC-b)*
*TaCLC-a-6DS-1*	TraesCS6D02G084300.1	3,146	787	4	5	49218043	49221583	PM	11	6D	*AT3G27170 (AtCLC-b)*
*TaCLC-a-6DS-2*	TraesCS6D02G084000.2	2,735	784	3	4	48866564	48869557	PM	11	6D	*AT3G27170 (AtCLC-b)*
*TaCLC-c1-3AS*	TraesCS3A02G125300.1	3,435	805	7	8	100885506	100893368	PM	12	3A	*AT5G33280 (AtCLC-c)*
*TaCLC-c1-3DS-1*	TraesCS3D02G126600.1	3,913	805	7	8	84568670	84582503	PM	12	3D	*AT5G33280 (AtCLC-c)*
*TaCLC-c1-3DS-2*	TraesCS3D02G126700.1	1,722	573	3	4	84587555	84589918	PM	8	3D	*AT5G33280 (AtCLC-c)*
*TaCLC-c2-3AL*	TraesCS3A02G390100.1	2,919	795	7	8	638325536	638329362	PM	11	3A	*AT5G33280 (AtCLC-c)*
*TaCLC-c2-3B*	TraesCS3B02G418700.1	2,964	795	7	8	655435367	655439266	PM	11	3B	*AT5G33280 (AtCLC-c)*
*TaCLC-c2-3DL*	TraesCS3D02G379600.1	2,862	794	7	8	496503737	496507536	PM	11	3D	*AT5G33280 (AtCLC-c)*
*TaCLC-e-3AL*	TraesCS3A02G253600.3	2,838	717	6	7	474962330	474970320	PM	11	3A	*AT4G35440( AtCLC-e)*
*TaCLC-e-3B*	TraesCS3B02G285500.1	2,415	717	6	7	457013924	457026915	PM	11	3B	*AT4G35440( AtCLC-e)*
*TaCLC-f1-6AL*	TraesCS6A02G283600.3	3,223	781	8	9	514703319	514709411	PM	9	6A	*AT1G55620 (AtCLC-f)*
*TaCLC-f1-6BL*	TraesCS6B02G312100.1	2,136	711	7	8	559340524	559348729	PM	9	6B	*AT1G55620 (AtCLC-f)*
*TaCLC-f1-6DL*	TraesCS6D02G264100.1	3,246	785	8	9	372824290	372830240	PM	9	6D	*AT1G55620 (AtCLC-f)*
*TaCLC-f2-7AS*	TraesCS7A02G240700.2	2,776	743	8	9	216343576	216349884	PM	9	7A	*AT1G55620 (AtCLC-f)*
*TaCLC-f2-7BS*	TraesCS7B02G136300.1	2,785	743	8	9	168313998	168321034	PM	10	7B	*AT1G55620 (AtCLC-f)*
*TaCLC-f2-7DS*	TraesCS7D02G239700.3	2,454	764	8	9	204246408	204253040	PM	10	7D	*AT1G55620 (AtCLC-f)*
*TaCLC-g2-2AL*	TraesCS2A02G517500.3	2,097	822	8	9	740847366	740855721	PM	11	2A	*AT5G33280 (AtCLC-g)*
*TaCLC-g2-2BL*	TraesCS2B02G546000.1	3,033	817	8	9	742813858	742822299	PM	11	2B	*AT5G33280 (AtCLC-g)*
*TaCLC-g2-2DL*	TraesCS2D02G519000.2	3,035	818	8	9	608915455	608923751	PM	11	2D	*AT5G33280 (AtCLC-g)*

SL, subcellular location; TMS, transmembrane segments; PM, plasma membrane; Accession of Arabidopsis, Ensembl Plant database accession number of TaCLC genes’ ortholog in *Arabidopsis thaliana*.

There were five *TaCLC* family members, all of which had homologous genes on the A, B, and D genomes including TaCLC-a, -c2, -f1, -f2, and -g2. However, TaCLC-c1 cluster and TaCLC-e cluster members contained only two homologous sequences. Comparatively, the TaCLC-c1 cluster member had one gene on the A genome and two genes on the D genome. The amino acid length of the TaCLC proteins ranged from 573 aa (TaCLC-g2-2AL) to 822 aa (TaCLC-c1-3DS-2). TaCLC proteins contained 8–12 transmembrane regions, of which 14 TaCLC proteins had 11 transmembrane regions and one TaCLC proteins (TaCLC-c1-3DS) had the least transmembrane regions with 8. The results of the visualized protein topology diagram are shown in Supplementary Figure S1.

### Phylogenetic and Structural Analyses of *Triticum aestivum* L. CLC Proteins

**FIGURE 1:**
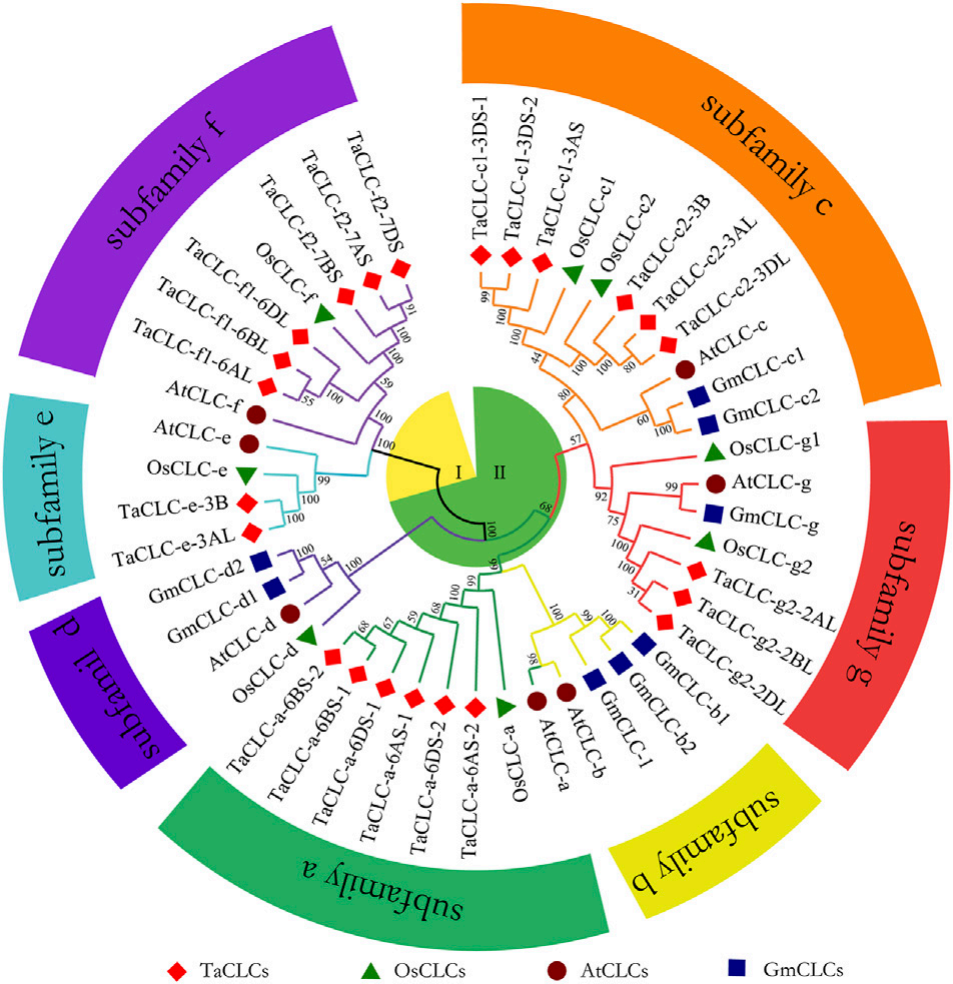
The phylogenetic tree of the CLC family of wheat, rice, soybean and Arabidopsis. The proteins belonging to each of four species are represented by different shapes and colors. The TaCLC family proteins were divided into seven subfamilies (-a, -b, -c, -d, -e, -f, and -g) and two subclasses (I and II), which are indicated with different shapes and colors of lines. The CLC proteins loci of wheat, rice, soybean, and Arabidopsis are listed in Supplementary Table S1. Ta, *Triticum aestivum*; Os, *Oryza sativa*; Gm, *Glycine max*; At, *Arabidopsis thaliana*.

In this study, the CLC family protein sequences of 23 *Triticum aestivum* (TaCLC), 7 *Arabidopsis thaliana* (AtCLC), 8 *Oryza sativa* (OsCLC), and 8 *Glycine max* (GmCLC) were aligned to construct a phylogenetic tree. This was performed using the neighbor-joining method with 1,000 bootstrap replication (Figure 1). According to the clustering criteria of rice and Arabidopsis, wheat TaCLC proteins were divided into seven clusters including TaCLC -a, -c1, -c2, -e, -f1, -f2 and -g2. Among these, the cluster -a was the largest group and contained six genes; comparatively, cluster -e was the smallest and only contained two members. The remaining five clusters all contained only three genes.

Structural analysis showed that cluster -a, cluster -c1, cluster -c2, and cluster -g2 all contained typical conserved regions GxGIPE (I), GKxGPxxH (II), and PxxGxLF (III). Cluster -e and cluster -f1/-f2 did not have this typical structure (Supplementary Figure S2). These results indicated that wheat *TaCLC* genes were divided into subclass I and subclass II according to the conserved structures of the *CLC* genes.

### Chromosomal Distribution of *Triticum aestivum* L. CLC Genes

**FIGURE 2:**
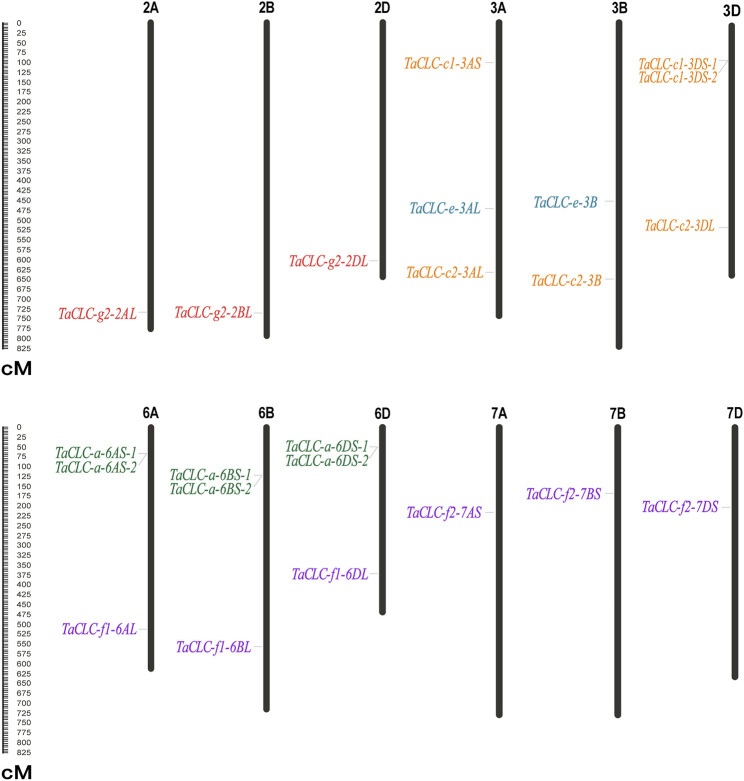
Distribution of *TaCLC* genes on chromosomes. The names of each chromosome are shown above. The gene names are indicated on the left and the starting positions are on the right side of the chromosomes. The *TaCLC* genes in each subfamily are specified by the same color. The chromosome lengths are shown in Mb (Millions of bases).

*TaCLC* genes were distributed on 12 chromosomes of wheat (Figure 2). The physical locations of *TaCLC* genes are shown in Table 1. The 23 *TaCLC* genes were unevenly distributed on chromosomes, of which 3A, 3D, 6A, 6B, and 6D chromosomes had three gene members. The distribution of the *TaCLC* genes in the A (8), B (7) and D (8) subgenomes was relatively balanced. This was consistent with the fact that nearly half of the family members have three homologous genes.

There were tandem repeat events in the cluster -a and cluster -c1, and they appeared on chromosomes in the form of tandem gene clusters. For example, *TaCLC-a-6AS-1* and *TaCLC-a-6AS-2*, *TaCLC-a-6BS-1* and *TaCLC-a-6BS-2*, *TaCLC-a-6DS-1* and *TaCLC-a-6DS-2*, and *TaCLC-c1-3DS-1* and *TaCLC-a-3DS-2* all formed different tandem gene clusters on chromosomes 6A, 6B, 6D, and 3D, respectively. This may also be the reason the number of TaCLC members was more than that of other species.

### Gene Collinearity Analysis

**FIGURE 3:**
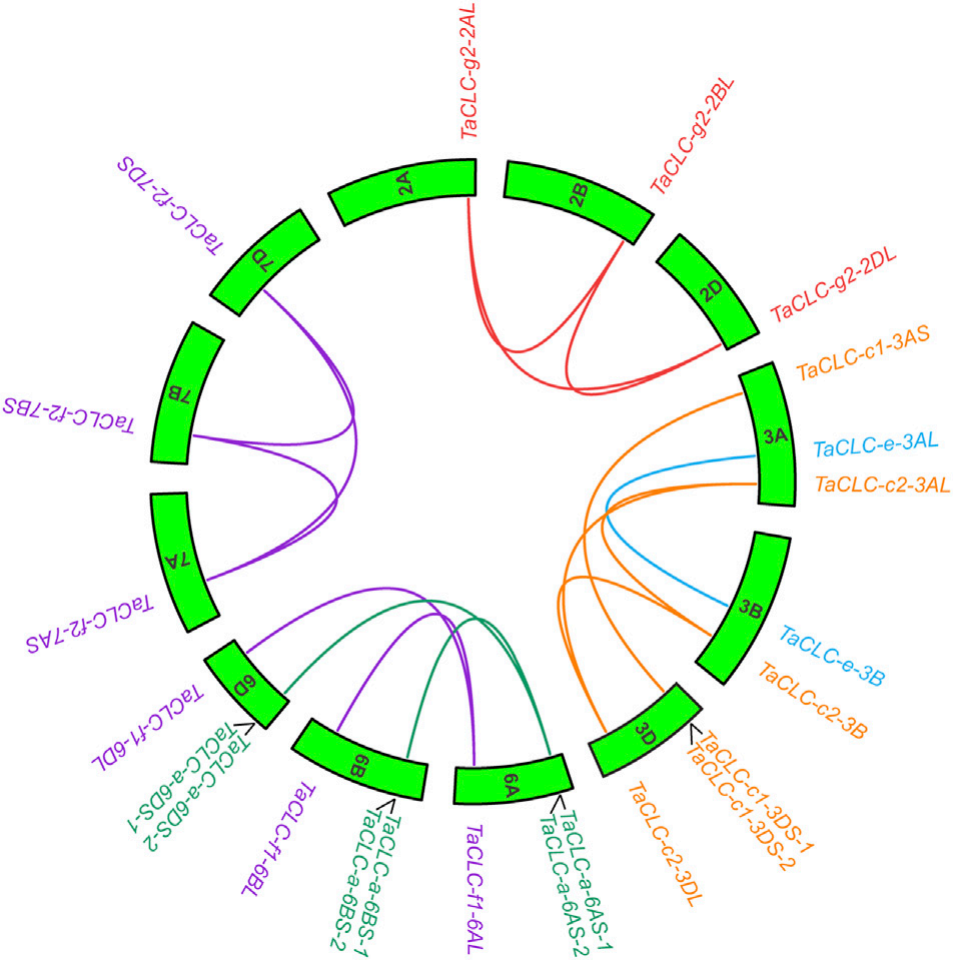
Collinearity analysis of *TaCLC* genes. *TaCLC* genes in five subfamilies (-a, -c, -e, -f, and -g) are represented by different colors of lines. Wheat chromosomes are represented by green boxes with chromosome names inside.

The results of intraspecies collinearity analysis showed that the 23 wheat *TaCLC* genes constituted 15 pairs of collinear genes (Figure 3). Among them, four genes including *TaCLC-a-6AS-2*, *TaCLC-a-6BS-2*, *TaCLC-a-6DS-2*, and *TaCLC-c1-6DS-2* had no collinearity with other *TaCLC* family genes and had tandem repeats. There was no collinearity between homologous genes *TaCLC-f1-6BL* and *TaCLC-f1-6DL*, between *TaCLC-a-6BS-1* and *TaCLC-a-6DS-1*, and no gene of cluster -c1 on the 3B chromosome. This also indicated that homologous fragment loss occurred simultaneously during evolution of the genes.

**FIGURE 4:**
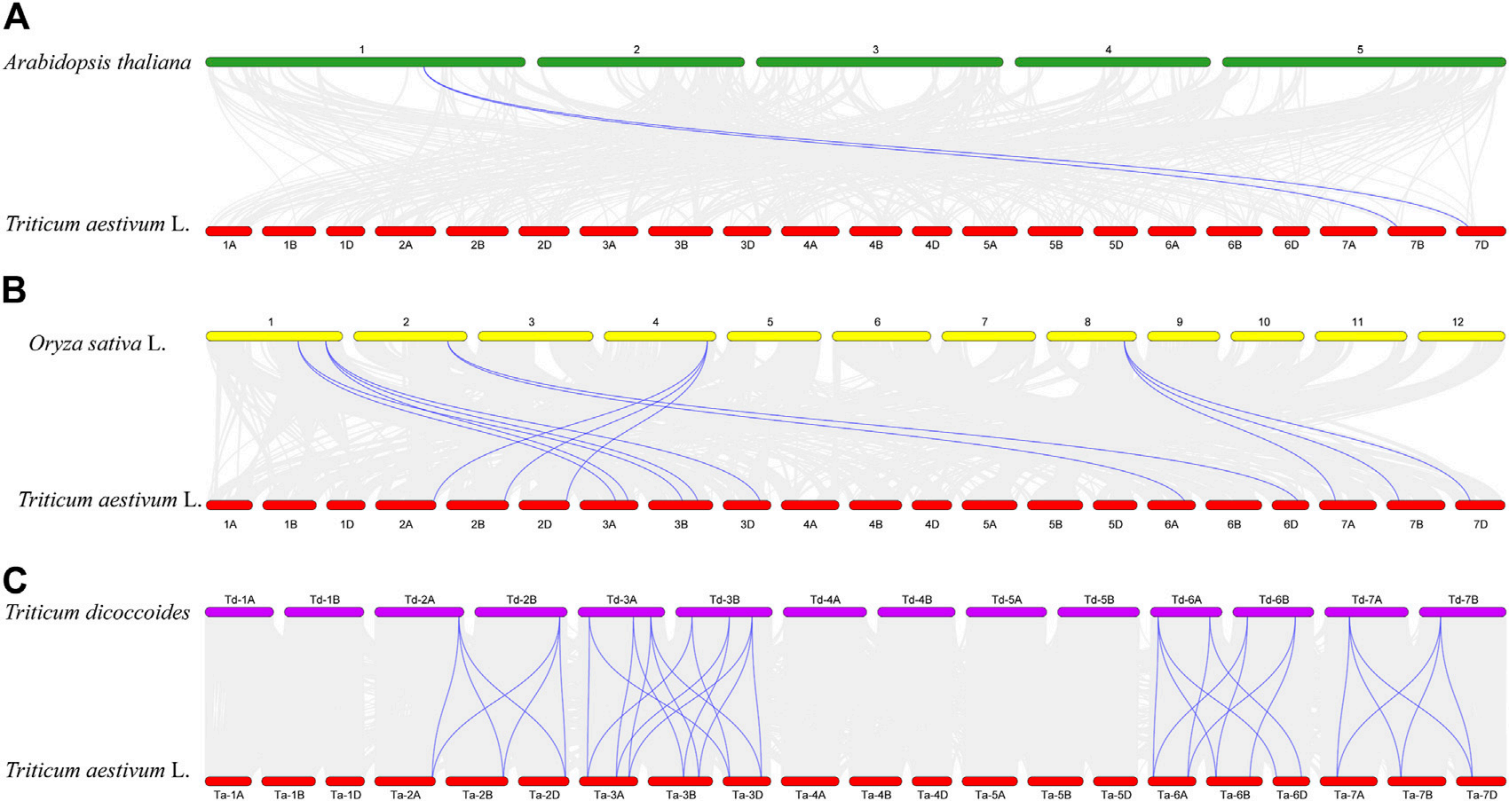
Collinearity analysis of *TaCLC* genes between *Triticum aestivum* L. and *Arabidopsis thaliana* **(A)**, *Oryza sativa* L. **(B)**, and *Triticum dicoccoides* **(C)**. The chromosomes of wheat, Arabidopsis, rice, and emmer are expressed in red, green, yellow and purple, respectively. The gray line represents all the collinearity gene pairs between the two species, and the blue bright line represents the collinearity gene pair between the *TaCLC* genes of wheat and the corresponding species. The names of each chromosome are shown above and below.

The results of collinearity analysis among species showed that *TaCLC* genes had collinearity with genes in genome of *Arabidopsis thaliana*, *Oryza sativa* L. and *Triticum dicoccoides* (Figure 4; Supplementary Table S4). The number of collinear gene pairs was different between species. Only two *TaCLC* genes between the genome of *Triticum aestivum* L. and *Arabidopsis thaliana* had collinearity, namely *TaCLC-f2-7BS* and *TaCLC-f2-7DS* (Figure 4A). The number of collinearity gene pairs in *Triticum aestivum* L. and *Oryza sativa* L. was 13, and the collinearity genes were mainly cluster -c2 and subfamily -e, -f, and -g (Figure 4B). *Triticum aestivum* L. and *Triticum dicoccoides* had 35 collinear gene pairs, which was the largest number (Figure 4C). Among them, 19 genes in *TaCLC* had collinearity with *Triticum dicoccoides* genes. There were 7, 6 and 6 *TaCLC* genes in the three subgenomes of A, B, and D of wheat, respectively, which had a collinearity relationship with the *Triticum dicoccoides* genes. This illustrated that the distribution of these 19 genes in the genome was uniform, and they were in a relatively conservative state during the evolution process from *Triticum dicoccoides* to *Triticum aestivum* L. Both *TaCLC-f2-7BS* and *TaCLC-f2-7DS* existed in the collinear gene pairs between *Triticum aestivum* L. and *Arabidopsis thaliana*, *Oryza sativa* L. and *Triticum dicoccoides*, indicating that these two genes may be ubiquitous in monocotyledons and dicotyledons. They formed before species differentiation and evolved for a longer time.

### *Triticum aestivum* L. CLCs Gene Structure and Conservative Motif Analysis

**FIGURE 5:**
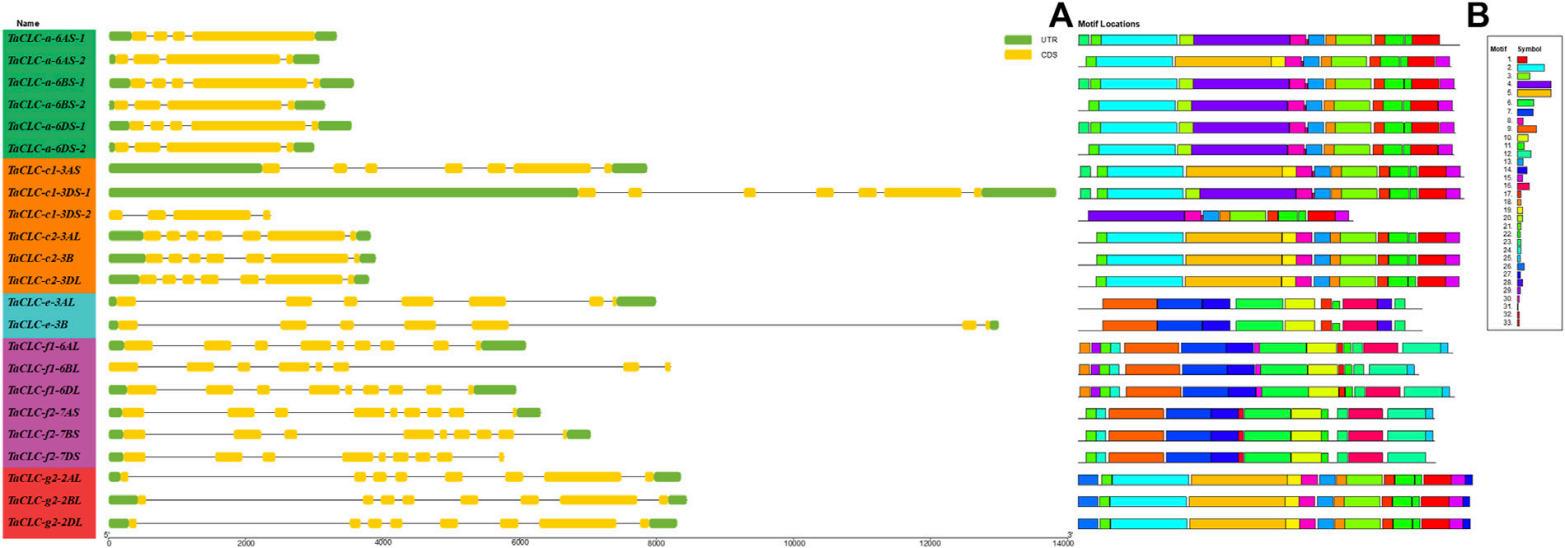
Gene structure and conservation motifs of *TaCLC* gene. **(A)** Gene structure. *TaCLC* genes are displayed in the order based on phylogenetic analysis of their proteins. Introns, exons and noncoding regions are represented with black lines, yellow boxes and green boxes, respectively. **(B)** Conserved motifs of TaCLC proteins. Thirty three motifs were identified and are marked with different colors.

TBtools software was used to compare the CDS sequences of 23 *TaCLCs* with their corresponding genome sequences. A visual structure map of the *TaCLC* genes was then obtained (Figure 5A). *TaCLC* genes contained 3–8 introns and 4–9 exons. The introns and exons of *TaCLC-a-6AS-1*, *TaCLC-a-6AS-2*, *TaCLC-a-6BS-2*, *TaCLC-a-6DS-2*, and *TaCLC-c1-3DS-2* had the smallest number among all identified *TaCLC* genes with 3 introns and 4 exons. *TaCLC-f1-6AL*, *TaCLC-f1-6DL*, *TaCLC-f2-7AS*, *TaCLC-f2-7BS*, *TaCLC-f2-7DS*, *TaCLC-g2-2AL*, *TaCLC-g2-2BL*, and *TaCLC-g2-2DL* had the largest number, with 8 introns and 9 exons. Exon and intron number among the same cluster of homologous genes also varied. For example, *TaCLC-f1-6AL* contained 8 introns and 9 exons, whereas *TaCLC-f1-6BL* contained 7 introns and 8 exons.

Using online tools to analyze conservative protein motifs, the motif structure of the same cluster of homologous genes was determined to be basically similar (Figure 5B). These 33 motifs were evenly distributed in 7 clusters, and the number of motifs in each cluster was also similar (Supplementary Table S5). The conserved motifs of TaCLC proteins were divided into two categories. The relatively conserved motifs in cluster -a and cluster -c1, -c2, and -g2 were different from those in cluster -e and cluster -f1 and -f2, indicating that the conserved motifs of the whole TaCLC gene family could be divided into two cases.

### Predictive Analysis of *Triticum aestivum* L. CLCs Gene Expression at Different Developmental Stages of Wheat

**FIGURE 6:**
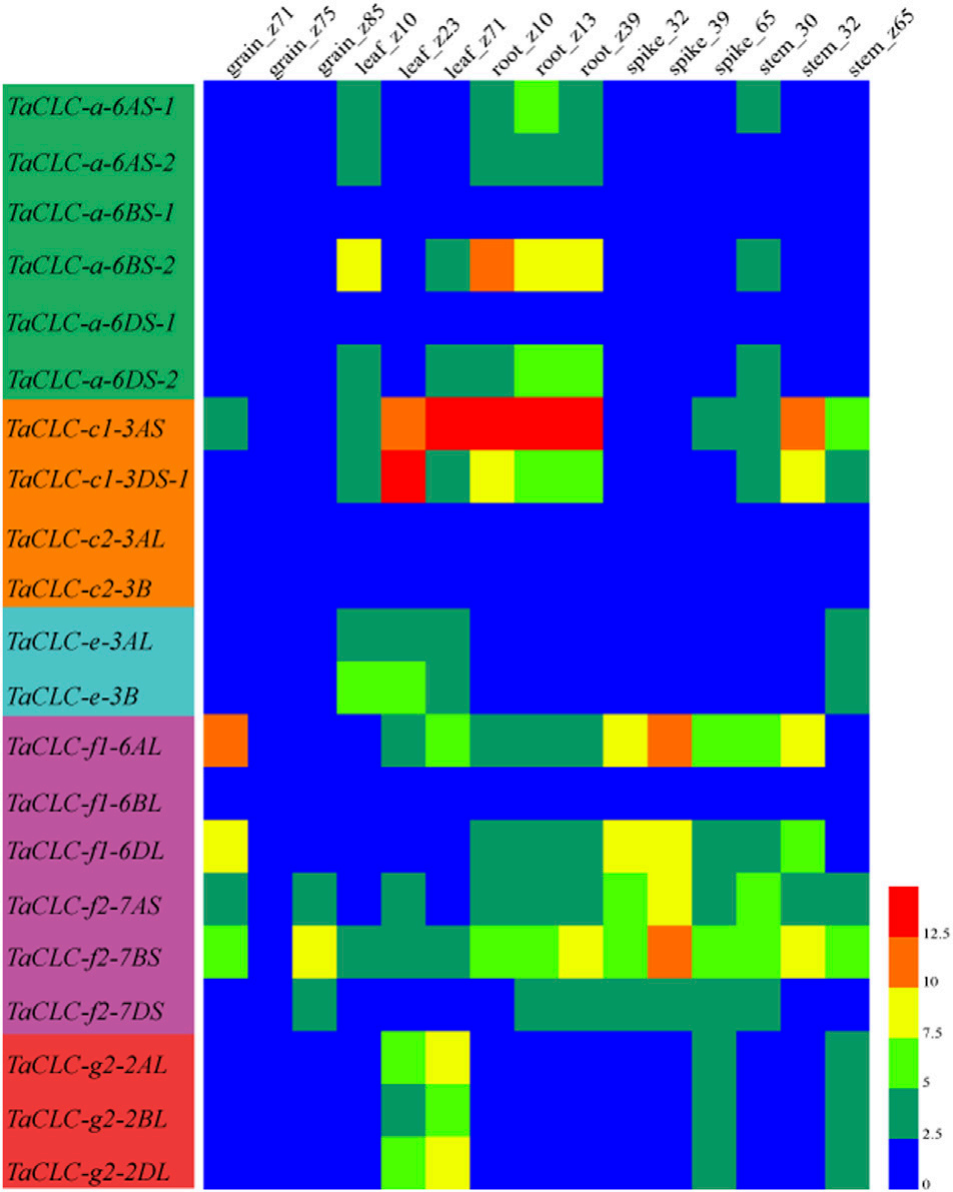
Heat map of *TaCLC* genes expressed at different developmental stages in wheat tissues. The values were obtained from the RNA-seq data of Chinese spring wheat in different tissues and periods provided by WheatExp database (Pearce et al., 2015). The values of 0–12.5 represent the lowest to the highest expression levels of the detected genes.

The expression patterns of *TaCLC* genes in wheat tissues (roots, stems, leaves, spikes, and grains) at different developmental stages were analyzed using available wheat RNA-seq databases (Figure 6). The developmental stages were as follows: z10 (seedling), z13 (three leaves), z23 (three tillers), z30 (spike at 1 cm), z32 (two nodes), z39 (meiosis), z65 (anthesis), z71 (2 DAA), z75 (14 DAA) and z85 (30 DAA). Of the 23 genes, the expression data on 21 genes were obtained from RNA-seq databases. The only missing genes were *TaCLC-c1-3DS-2* and *TaCLC-c2-3DL*. *TaCLC-c2-3AL*, *TaCLC-c2-3B*, *TaCLC-a-6BS-1*, *TaCLC-a-6DS-1*, and *TaCLC-f1-6BL* had low expression level in all detected periods and tissues. This was especially true for the z75 (14 DAA) period, which had very low gene expression. The expression levels of *TaCLC-c1-3AS* in roots of z10 (seedling stage), z13 (three leaves stage), z39 (meiosis stage), and leaves of z71 (2 DAA stage), as well as *TaCLC-c1-3DS-1* in leaves of z13 (three-leaves stage) were all higher in all examined *TaCLC* genes.

*TaCLC-a-6BS-2* had the highest expression level in cluster -a, while all the genes of cluster -c2 were not expressed. The expression level of *TaCLC-c1-3AS* was higher than that of *TaCLC-c1-3DS-1* during all the stages except for the leaves of z23 (three tillers) in cluster -c1. In cluster -e, the expression levels of *TaCLC-e-3AL* and *TaCLC-e-3B* were similar in all stages and tissues except in leaves of z10 (seedling) and z13 (three leaves). Among the three cluster -f1 genes, the expression level of *TaCLC-f1-6AL* was the highest and expressed in all tissues at each stage, while the expression level of *TaCLC-f2-7BS* was the highest among the three genes of cluster -f2. In cluster -g2, the expression levels of *TaCLC-g2-2AL* and *TaCLC-g2-2DL* genes in leaves of z23 (three tillers) and z71 (2 DAA) stages were higher than those of *TaCLC-g2-2BL*.

### Expression Analysis of *Triticum aestivum* L. CLCs Gene Under Low Nitrogen Stress or Salt Stress

**FIGURE 7:**
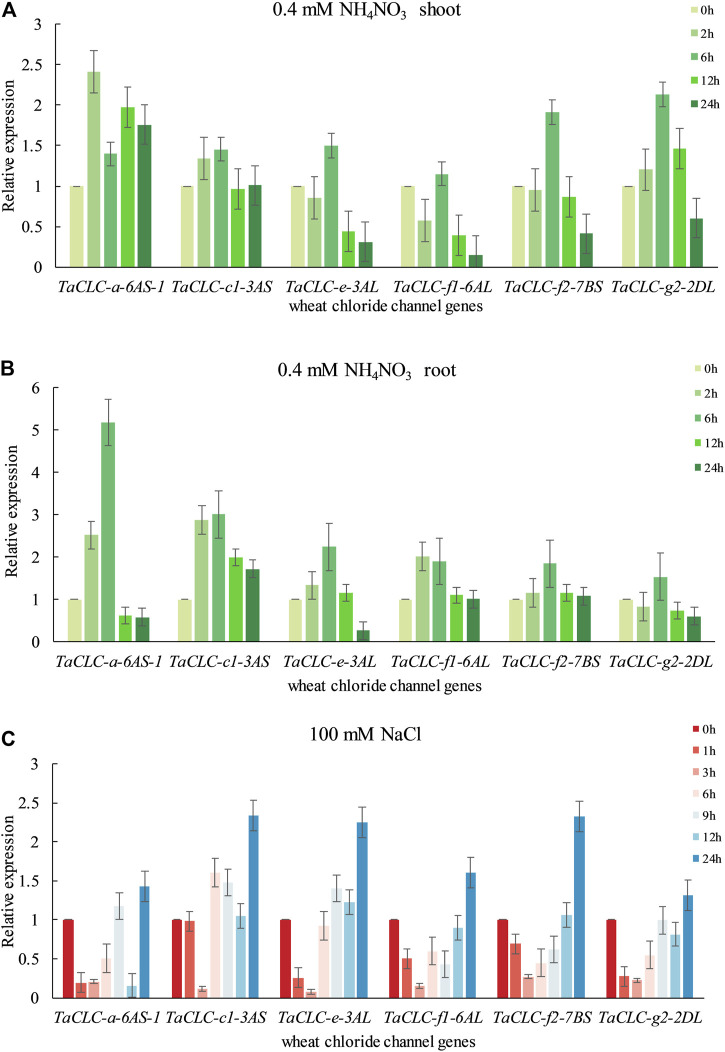
Quantitative real-time PCR validation of *TaCLC* genes in wheat seedlings under different stresses. **(A)** Expression of *TaCLC* genes in the wheat shoot tissue after 0.4 mM NH_4_NO_3_ treatment for 0, 2, 6, 12 and 24 h. **(B)** Expression of *TaCLC* genes in the wheat root after 0.4 mM NH_4_NO_3_ treatment for 0, 2, 6, 12 and 24 h. **(C)** Expression of *TaCLC* genes in wheat seedlings after 100 mM NaCl treatment for 0, 1, 3, 6, 9, 12 and 24 h.

According to the predictive analysis of gene expression heat map, we selected *TaCLC-a-6AS-1*, *TaCLC-c1-3AS*, *TaCLC-e-3AL*, *TaCLC-f1-6AL*, *TaCLC-f2-7BS*, *TaCLC-g2-2DL*, and *TaCLC-c2-3AL* from each cluster to analyze the expression patterns under low nitrogen stress or salt stress using qRT-PCR. The gene expression of this family of genes was determined to be up-regulated under conditions of low nitrogen stress or salt stress except for *TaCLC-c2-3AL* (Figure 7). This finding was consistent with the data from the wheat RNA-seq database that the genes of cluster -c2 were not expressed across all stages.

The expression patterns of different genes in different parts were also diverse under low nitrogen stress (Figures 7A,B). Among them, the expression of the *TaCLC-a-6AS-1* gene was at its highest level after 2 h in the shoot and at its highest level at 6 h in the root tissues when compared with the untreated tissues. Under conditions of low nitrogen stress, expression patterns in the shoot tissues were mainly divided into three types (Figure 7A). The expression levels of the other five genes were the highest after 6 h of low nitrogen stress, except for the *TaCLC-a-6AS-1* gene. The expression patterns of *TaCLC-e-3AL*, *TaCLC-f1-6AL*, and *TaCLC-f2-7BS* genes were all down-regulated and then up-regulated. Comparatively, the expression patterns of the *TaCLC-c1-3AL* and *TaCLC-g2-2AL* genes were slowly up-regulated and then down-regulated. The expression pattern of the *TaCLC-a-6AS-1* gene was completely different from that of the other five genes, indicating that *TaCLC-a-6AS-1* belonged to a gene type that was stress-induced and involved rapid up-regulation. Notably, its expression level remained higher than when without stress. In the root parts and under conditions of low nitrogen stress, the expression patterns of the other five genes were first up-regulated and then down-regulated. This occurred across all genes except for the *TaCLC-g2-2DL* gene. The expression of *TaCLC-a-6AS-1*, *TaCLC-c1-3AS*, *TaCLC-e-3AL*, and *TaCLC-f2-7BS* all reached their highest respective levels at 6 h after low nitrogen stress treatment (Figure 7B). Only *TaCLC-f1-6AL* reached its highest expression level at 2 h; the expression pattern of the *TaCLC-g2-2DL* gene was first down-regulated, up-regulated, and finally down-regulated.

As shown in Figure 7C, all six genes reached their highest respective expression levels after 24 h salt stress treatment. The relative expression levels of *TaCLC-c1-3AS*, *TaCLC-e-3AL*, and *TaCLC-f2-7BS* were all higher than those of the other genes. The other five genes showed a down-regulated expression pattern under transient salt stress, and began to show an upward tendency at approximately 6 h treatment, except for the *TaCLC-c1-3AS* gene. Three genes—*TaCLC-a-6AS-1*, *TaCLC-e-3AL*, and *TaCLC-g2-2DL—*were all down-regulated, and finally up-regulated across 6–24 h. The *TaCLC-f1-6AL* and *TaCLC-f2-7BS* genes were both down-regulated to their lowest levels at 3 h and then up-regulated to their highest level at 24 h. The expression of *TaCLC-c1-3AS* did not change much before 1 h, and reached its lowest level at 3 h. After a brief up-regulation from 3 to 6 h, its expression began to be down-regulated from 6 to 12 h. Its expression was finally up-regulated to its highest level from 12 to 24 h.

### Functional Complementation of *Triticum aestivum* L. CLC Members in Yeast Mutant

**FIGURE 8:**
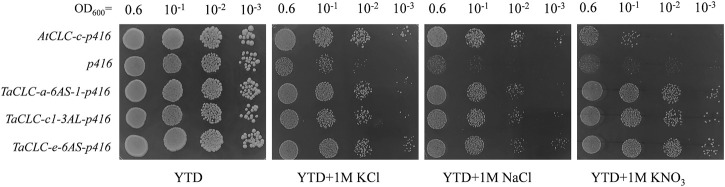
Yeast *Δgef1* mutant complementation experiment. Yeast transformed with empty vector (p416) was used as a negative control and *AtCLC-c* was used as a positive control. The tested YTD (1% yeast extract/2% tryptone/2% dextrose) media supplemented with different concentrations of compounds (1 M KCl/1 M NaCl/1 M KNO_3_) are indicated under each panel. Serial dilutions (10^−1^) of yeast culture were plated.

The budding yeast *S. cerevisiae* has been shown to be an excellent model for studying ion transport properties and physiological function of ion homeostasis (Xu et al., 2013). The existence of mutant strains lacking their own transport systems has provided an efficient tool for the molecular study of transporters from higher eukaryotes upon their expression in yeast cells (Xu et al., 2008). In *S. cerevisiae*, the *GEF1* gene encodes a single putative CLC chloride channel/transporter. The corresponding mutant strain—*Δgef1—*lacks GEF1 gene and is sensitive to extracellular cations (Lv et al., 2009). Studies have shown that the *AtCLC-c* gene compensates for the inhibited growth of *Δgef1* deletion mutant yeast cells on YTD medium containing high concentrations of either NaCl or KCl (Lv et al., 2009). The growth of *Δgef1* cells is not significantly different from that of wild type cells, indicating that the toxicity of these salts on the growth of *Δgef1* cells is more related to the properties of anions than the properties of cations (Lv et al., 2009). In this study, we investigated the function of *TaCLC-a-6AS-1*, *TaCLC-c1-3AS*, and *TaCLC-e-3AL* using the yeast strain YJR040w (*Δgef1*). The AtCLC-c-p416 recombinant plasmid vector was transferred into the yeast strain YJR040w and used as a positive control, while the p416 vector was transferred into the yeast strain YJR040w and used as a negative control. The results illustrated that the three genes *TaCLC-a-6AS-*1, *TaCLC-c1-3AS*, and *TaCLC-e-3AL* also compensated for the inhibitory effects of the GEF1 deletion mutant yeast YJR040w cells on the growth of YTD medium containing either 1 M NaCl or 1 M KCl (Figure 8). All the test transgenic CLC genes partially restored the growth function of mutant yeast cells YJR040w on YTD medium containing 1 M KNO_3_, indicating that *TaCLC-a-6AS-1*, *TaCLC-c1-3AS*, and *TaCLC-e-3AL* in wheat and *AtCLC-c* in Arabidopsis had certain NO_3_^−^ transport ability. These results showed that the three selected TaCLC genes compensated for the fact that the GEF1 mutant YJR040w blocks transport of Cl^−^ or NO_3_^−^ after the deletion of the CLC genes. The proteins encoded by these three genes of *TaCLC-a-6AS-1*, *TaCLC-c1-3AS*, and *TaCLC-e-3AL* exhibited anion transport activity of Cl^−^ or NO_3_^−^.

## Discussion

The main nitrogen sources for plants are either nitrate nitrogen or ammonium nitrogen. Under natural field conditions, the content of nitrate nitrogen in soil is much higher than that of ammonium nitrogen. Therefore, understanding the efficient absorption mechanism(s) of nitrate nitrogen in crops would provide a theoretical basis for improving crop absorption—potentially by using biotechnological approaches (Meng et al., 2019). Currently, there remains little information on the CLC gene family in plants, with information relegated to only some species like *Arabidopsis thaliana*, *Glycine max*, *Oraza sativa*, and *Zea mays* (Diedhiou and Golldack, 2006; Lv et al., 2009; Wang et al., 2015; Wei et al., 2019). For wheat, there remains no previous work regarding a genome-wide identification and analysis of wheat *TaCLC* family genes. With the successful completion of genome sequence of wheat, it is now possible to identify members of the TaCLC gene family at the whole genome level (Appels et al., 2018). According to the homologous relationship of *CLC* genes in different species, a total of 23 *TaCLC* genes in seven clusters were identified using the wheat genome database. Their homologous relationships, gene structure, chromosomal localization, and expression pattern were subsequently analyzed. These results will lay the foundation for the functional study of *TaCLC* genes and provide a theoretical reference for understanding nitrate transport in wheat.

Across species, the name of the CLC protein family remains confusing because different researchers have updated the data at different stages. For example, in addition to *ZmCLC1*, *ZmCLC2*, and *ZmCLC3* genes in maize, there are also *ZmCLC-a, -b, -c, -d* and other genes (Yu et al., 2017). Moreover, the rice *OsCLC* genes used in this study were originally named *OsCLC 1–7*. After comparing, we corrected the name to *OsCLC-a*, *OsCLC-c1*, and other more consistent gene names (Supplementary Table S1). In this study the *TaCLC* genes were named according to the Arabidopsis classification criteria ( -a, -b, -c, -d, -e, -f, -g). During the structural analysis of the TaCLC protein, we found that the TaCLC-a/-c1/-c2/-g2 contained a common conserved domain that was also found in other species. The x residue in the conserved region (I) of the TaCLC-a cluster was proline, while in the TaCLC-c1/-c2/-g2 it was serine. Therefore, we speculated that the function of the TaCLC-a cluster of genes was similar to that of the CLC-a cluster of genes in other species. Moreover, NO_3_^−^ was preferentially transported, which may indicate the gene’s role as a NO_3_^−^/H^+^ exchanger. The TaCLC-c1/-c2/-g2 cluster genes have a higher affinity for Cl^−^ and preferentially transport Cl^−^ (Nedelyaeva et al., 2019). This hypothesis is also consistent with the up-regulation of the *TaCLC-a-6AS-1* gene in our qRT-PCR results.

In plants, the functional research of *CLC* genes has mainly focused on the physiological and molecular regulatory functions of either Cl^−^ or NO_3_^−^ absorption, transportation, and chloride (salt) tolerance (Li et al., 2006; Nakamura et al., 2006; Wei et al., 2013; Wong et al., 2013; Liao et al., 2018; Nedelyaeva et al., 2018; Nedelyaeva et al., 2019). Therefore, genetic research into the CLC-a/-c cluster is more extensive. In Arabidopsis, studies have found that *AtCLC-e* and *AtCLC-a* also have interconnected transporters in the nitrate assimilation pathway (Monachello et al., 2009). In addition, *AtCLC-a* plays a different role in the regulation of guard cell expansion in that *AtCLC-a* promotes anion accumulation during light-induced guard cell expansion and stomata opening (Wege et al., 2014). The N-terminus of *AtCLC-a* is phosphorylated by *AtSnRK 2.6* (*AtOST1*, *At4g33950*), which mediates stoma closure by excluding anions (Wege et al., 2014). *AtPP2A-C5* (*At1g69960*) interacts with *AtCLC* family genes, and *AtPP2A-5C* overexpression in plants increases the activity of *AtCLC-c.* This increases the ability of Cl^−^ to enter into vacuoles and improves salt tolerance of plants (Hu et al., 2017). Based on the hypothesis of homologous sequence alignment, it was speculated that the *TaSnRK 2.6* (*TaOST1, TraesCS5D02G081700*) and TaCLC-a cluster of genes*,* as well as the *TaPP2A-5C* (*TraesCS6D02G1714000*) and TaCLCs should be related.

Among plants, only three *PtCLC* genes have been identified in *Populus trichocarpa* (Yu et al., 2017), and 22 *GhCLC* genes in upland cotton (*Gossypium hirsutum* L.) (Liu X. et al., 2020). In most cases, the number of *CLC* genes identified in other plants is 5–7, while the number of *TaCLC* genes identified in wheat is up to 23 genes. The physical location and collinearity analysis of the gene on its corresponding chromosome indicated that there was a tandem repeat in the evolution of wheat *TaCLC* genes, which might be the reason why there are more *TaCLC* genes in wheat than other species. In this study, the TaCLC-b/-d subfamily genes were not found in the wheat genome, indicating that the fragment and gene loss of the homologous gene in this family may have simultaneously occurred. It may also be the artificial deletion caused by the incompleteness of the conserved domain in the previous analysis of 34 *CLC* genes annotated in the Emsembl Plant database. For example, the CD-search results of proteins encoded by the *AtCLC-b* and *AtCLC-d* genes in NCBI did not have a complete voltage gated CLC domain (PF00654), so we removed TaCLC-b/-d in the identification of the wheat TaCLC gene family. At the same time, we also named the 11 removed genes which had incomplete domains according to the phylogenetic tree (Supplementary Figure S3; Supplementary Table S6). In the *CLC* genes identified in both *Camellia sinensis* and *Nicotiana tabacum*, the number of motifs used was 20 and 24, respectively (Zhang et al., 2018; Xing et al., 2020). According to the HMM logo in the Pfam database, the motifs number of TaCLCs was determined to be 33 and the structure type was similar to the motif’s position structural type of the CLC which can be divided into two types. In addition, two different motif position structure types were also found in tobacco, which indicated that the functions of the *CLC* genes have become diversified. RNA-seq database analysis showed that the function and expression of each gene were different. Notably, even homologous genes played different functions at different development stages, such as *TaCLC-a-6AS-1*, *TaCLC-a-6BS-1*, and *TaCLC-a-6DS-1*. It seems that evolutionary pressures can extend the members of the gene family (Abdullah et al., 2021; Musavizadeh et al., 2021). Moreover, mutations in coding sites and promoter regions can affect the function of members of the gene family (Faraji et al., 2021).

To analyze the expression patterns of the TaCLC gene family under low nitrogen stress or salt stress, seven genes from each cluster of the TaCLC gene family were selected for fluorescence qRT-PCR. The results showed that the family genes were up-regulated under short-term low nitrogen stress or salt stress. This was true except for the TaCLC-c2 cluster genes, which basically had no expression across the various periods of stress. The expression pattern of the CLC gene family in most species has been focused on across different tissue types as well as during long-term abiotic stress (Lv et al., 2009; Jossier et al., 2010; Wei et al., 2015; Zhang et al., 2018; Liu X. et al., 2020). However, the expression patterns of *TaCLC* at each time point were different from those of other species. For instance, *AtCLCa-d* had a relatively stable expression abundance at all developmental stage. The expression level of *AtCLC-e* was equivalent to that of *AtCLC-f*, but was significantly lower than that of subclass I genes (Wei et al., 2015). In tobacco, all expressed *NtCLC* genes had low expression levels in the roots. After 7 days of salt stress (300 mM NaCl), the expression levels of multiple *NtCLC* genes were all significantly up-regulated (Zhang et al., 2018). In pomegranate (*Punica granatum*), the expression level of the *PgCLC* genes under salt stress was high in leaves and low in roots. Moreover, *PgCLC* genes have been shown to affect the accumulation of Cl^−^, SO_4_^2−^, and NO_3_^−^ in pomegranate tissues under salt stress (Liu C. et al., 2020). Our results showed that all *TaCLC* genes were expressed under low nitrogen stress conditions over the short term. For low nitrogen stress, the response of the *TaCLC-a-6AS-1* gene revealed it had the highest expression level. The relative expression levels of the *TaCLC-c1-3AS*, *TaCLC-e-3AL*, and *TaCLC-g2-2DL* genes were all higher under salt stress. These results were consistent with the characteristics that cluster -a had NO_3_^−^ transport capacity and clusters -c1/-g2 had Cl^−^ transport capacity in the CLC gene family. Based on the results of qRT-PCR analysis, we speculated that *TaCLC* genes (except the genes of TaCLC-c2 cluster) could respond to anion deficiency stresses.

The budding yeast, *S. cerevisiae* allows for large-scale, genome-wide analyses in a fast and economically efficient manner. Work with *S. cerevisiae* allows for the discovery and/or characterization of many aspects of ion transporter function (Locascio et al., 2019). Studies have shown that *GEF1*, as a chloride channel gene, maintains the charge balance in yeast cells. Through its acidic interior, the cation can be localized in either the internal organs or vacuoles of the cell, thus playing a role in cation detoxification (Gaxiola et al., 1998; Li et al., 2015; Nagatsuka et al., 2017). The mutant yeast *Δgef1* lacks the chloride ion channel gene and is blocked when transporting Cl^−^ in the intracellular vesicles (vacuoles or Golgi apparatus) of yeast cells (Hechenberger et al., 1996). It also has hypersensitivity to several extracellular cations. At present, *Arabidopsis thaliana*, *Glycine max*, *Oryza sativa* L., and *Suaeda altissima* (L.) have been studied using yeast mutants (Nakamura et al., 2006; Lv et al., 2009; Nedelyaeva et al., 2019; Wei et al., 2019). In this study, various chlorides, sulfates, and nitrates were respectively added to SD, SR, YPEG, YPG, YPD, or YTD media to determine whether *TaCLC* genes inhibit the sensitivity of yeast mutant cells to metal cations and to determine their anion transport function. In these experiments, we found that the growth of *Δgef1* yeast cells with YTD medium as the culture substrate was most suitable for YJR040w mutation. This stood in contrast to using SD, SR, YPEG, YPG, or YPD media as the culture substrate.

Previous studies showed that *AtCLC-c* gene had the ability to transport Cl^−^ (Lv et al., 2009). In our study, we found that when transformed one of the genes *TaCLC-a-6AS-1*, *TaCLC-c1-3AS*, and *TaCLC-e-3AL*, transgenic yeast mutant strains had a strong ability to transport Cl^−^ compared with control yeast strain, albeit they did not reach the tolerance of *AtCLC-c* gene transformation (Figure 8). It indicated that a large number of *TaCLC* genes may simultaneously play roles in the transport of anions such as Cl^−^ or NO_3_^−^. For a single *TaCLC* gene, its anion transport capacity was not very strong. In addition, *AtCLC-c* gene-transformed yeast mutant strains have not been studied in a medium containing KNO_3_. In our study, we found that *AtCLC-c* gene-transformed yeast mutant strains could transport NO_3_^−^ and inhibit the cation hypersensitivity in yeast GEF1 mutants (Figure 8). There was no difference in the growth of *TaCLC-a-6AS-1*, *TaCLC-c1-3AS*, and *TaCLC-e-3AL* in YTD medium containing 1 M KNO_3_, and the three genes had similar NO_3_^−^ transport ability without exogenous Cl^−^ interference. According to the regularity of the conserved motifs of the CLC and the analysis of the TaCLC protein sequences, there may be differences in preferential transport ability of NO_3_^−^ or Cl^−^ among *TaCLC-a-6AS-1*, *TaCLC-c1-3AS*, and *TaCLC-e-3AL* in the case of exogenous Cl^−^ interference.

## Conclusion

In this study, a genome-wide identification of *CLC* genes in wheat was performed and 23 *TaCLC* genes were identified. The gene structure, chromosomal location, conserved motif and expression pattern of the members of the family were then analyzed. The family was divided into two main subclasses (I and II) and seven clusters (-a, -c1, -c2, -e, -f1, -f2, and -g2). The 23 *TaCLC* genes of the family were unevenly distributed on wheat chromosomes and some genes in the cluster had tandem duplication. *TaCLC* gene expression was illustrated using qRT-PCR, and the results showed that the expression pattern of this gene family was induced by low nitrogen stress or salt stress except for *TaCLC-c2* which was from subfamily -c. The function of some *TaCLC* genes was studied using yeast mutant strains. The results of yeast mutant complementation experiments showed that *TaCLC-a-6AS-1*, *TaCLC-c1-3AS*, and *TaCLC-e-3AL* all had anion transport functions for NO_3_^−^ or Cl^−^ and compensated the hypersensitivity of yeast GEF1 mutants in restoring anion-sensitive phenotype. Collectively, these results provide theoretical reference for studying the response of *TaCLC* family genes to low nitrogen stress and the physiological functions of anion transport in wheat.

## Data Availability

All datasets generated for this study are included in the article/Supplementary Material.
