# Supplementary material for: Genome-Wide Identification and Functional Characterization of the Chloride Channel TaCLC Gene Family in Wheat (Triticum aestivum L.)
Source: Front Genet. 2022 Mar 16;13:846795. doi: 10.3389/fgene.2022.846795 (PMC8966409; doi:10.3389/fgene.2022.846795)

Supplementary Fig. S1 Visual protein topology of TaCLCs gene.Gene names were marked on each Visual protein topology


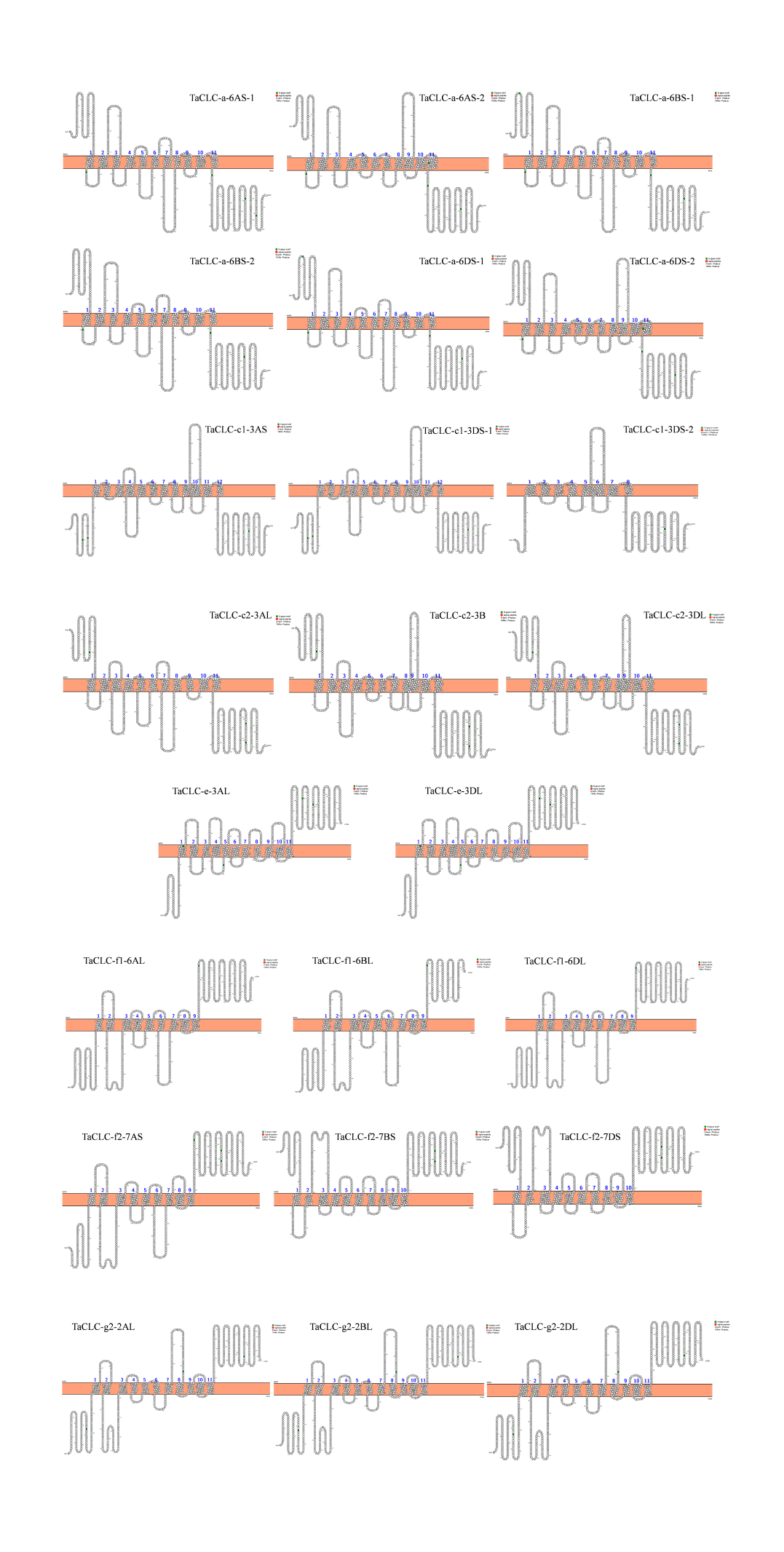


Supplementary Fig. S2 TaCLCs gene protein sequence alignment results


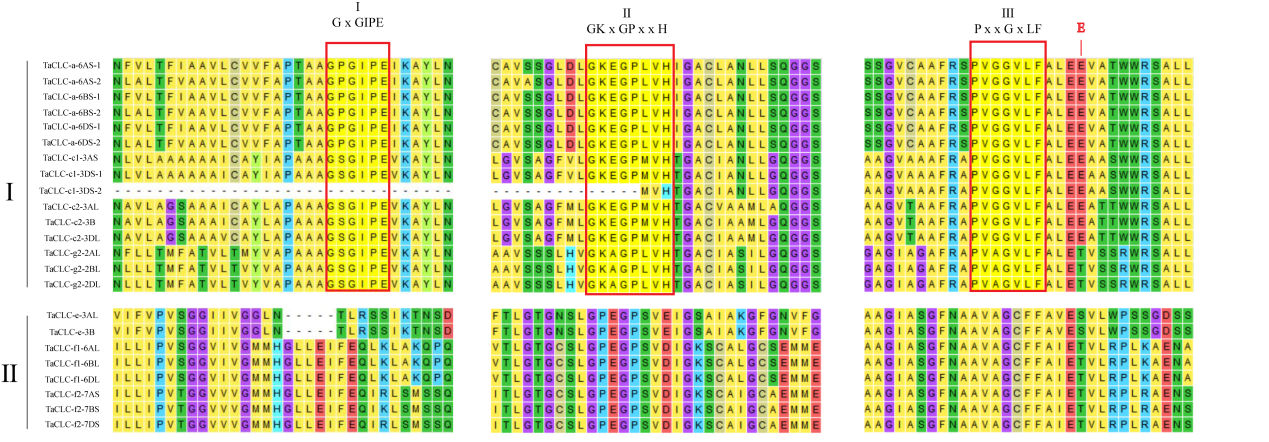


Supplementary Fig. S3 Genetic evolution tree of CLC gene containing incomplete domain


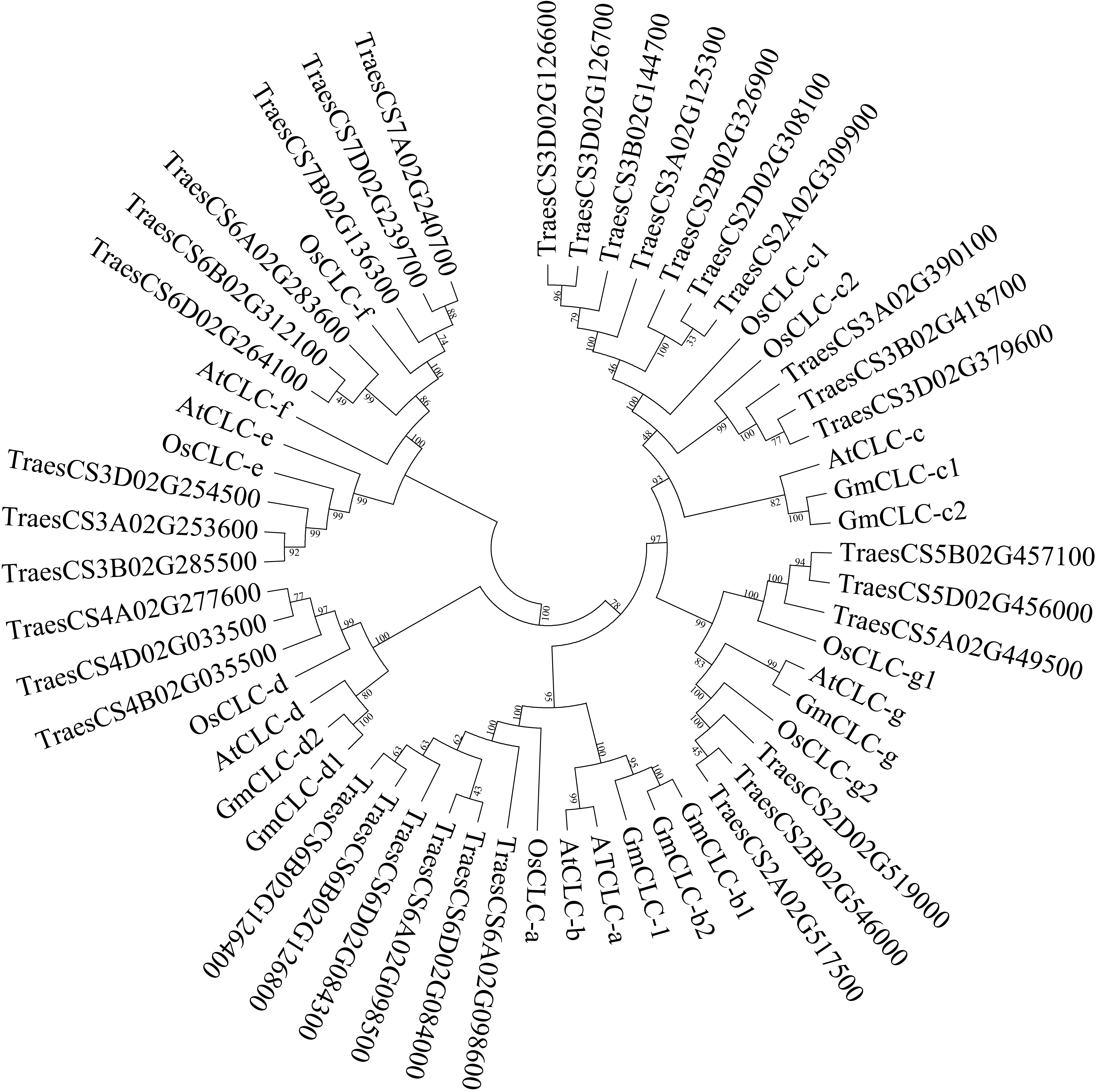

Supplement: Supplementary file 1 [file Table1.DOCX]
