# Supplementary figures and images for: Genome-Wide Identification and Functional Characterization of the Chloride Channel TaCLC Gene Family in Wheat (Triticum aestivum L.)
Source: Front Genet. 2022 Mar 16;13:846795. doi: 10.3389/fgene.2022.846795 (PMC8966409; doi:10.3389/fgene.2022.846795)

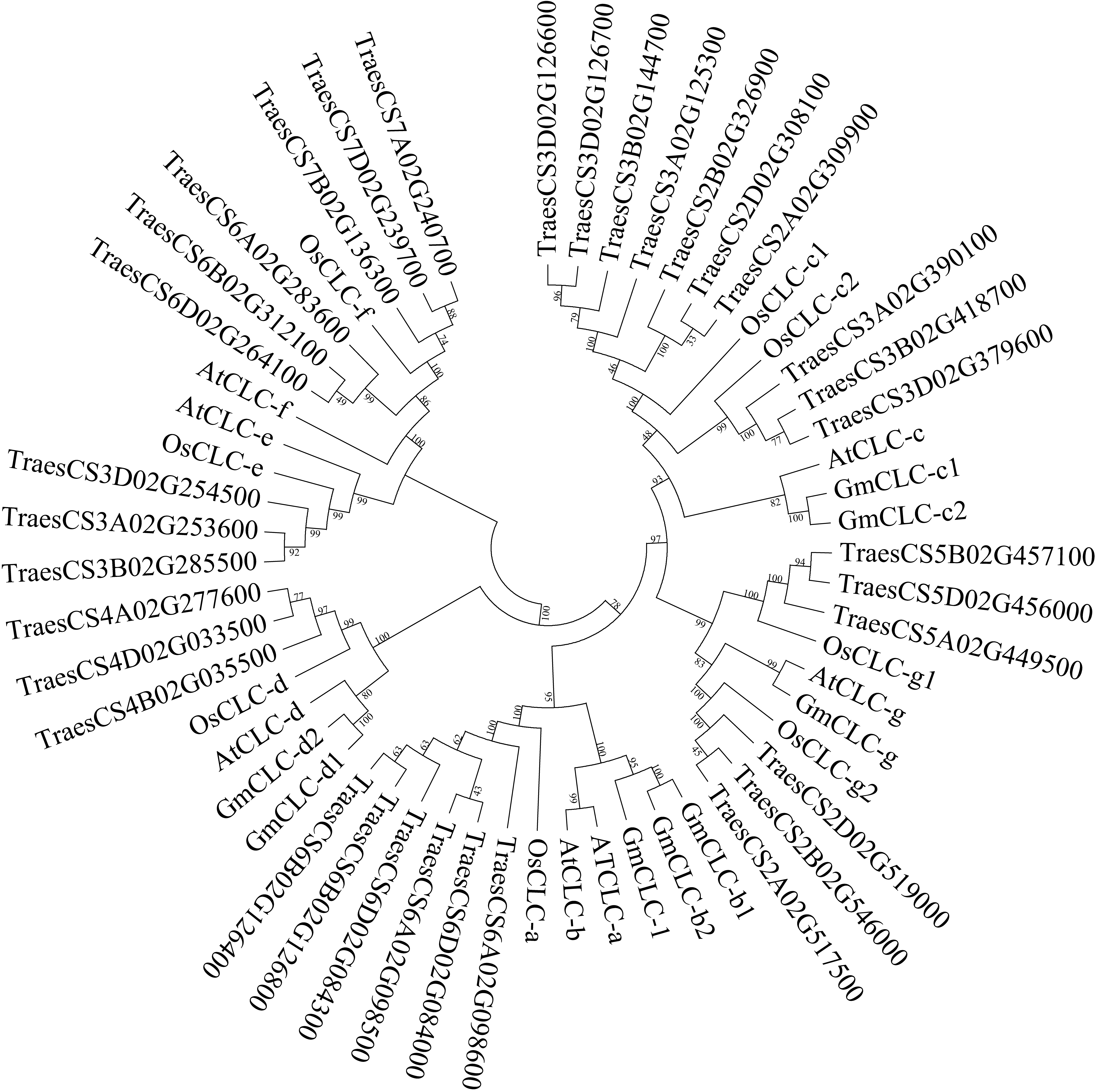

Supplement: Supplementary file 2 [file Image3.tif]

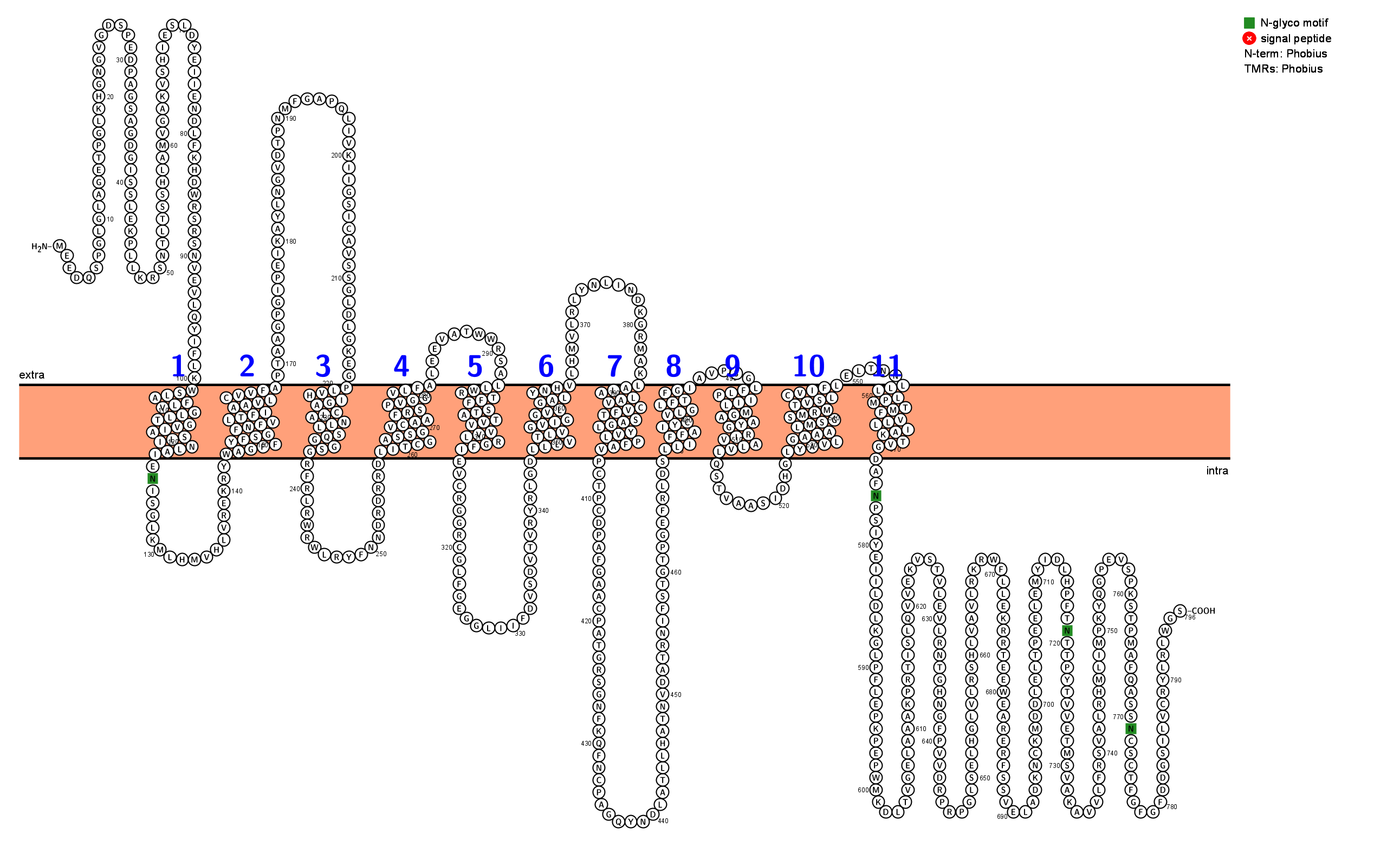

Supplement: Supplementary file 3 [file DataSheet1.ZIP › TaCLC-a-6AS-1.png]

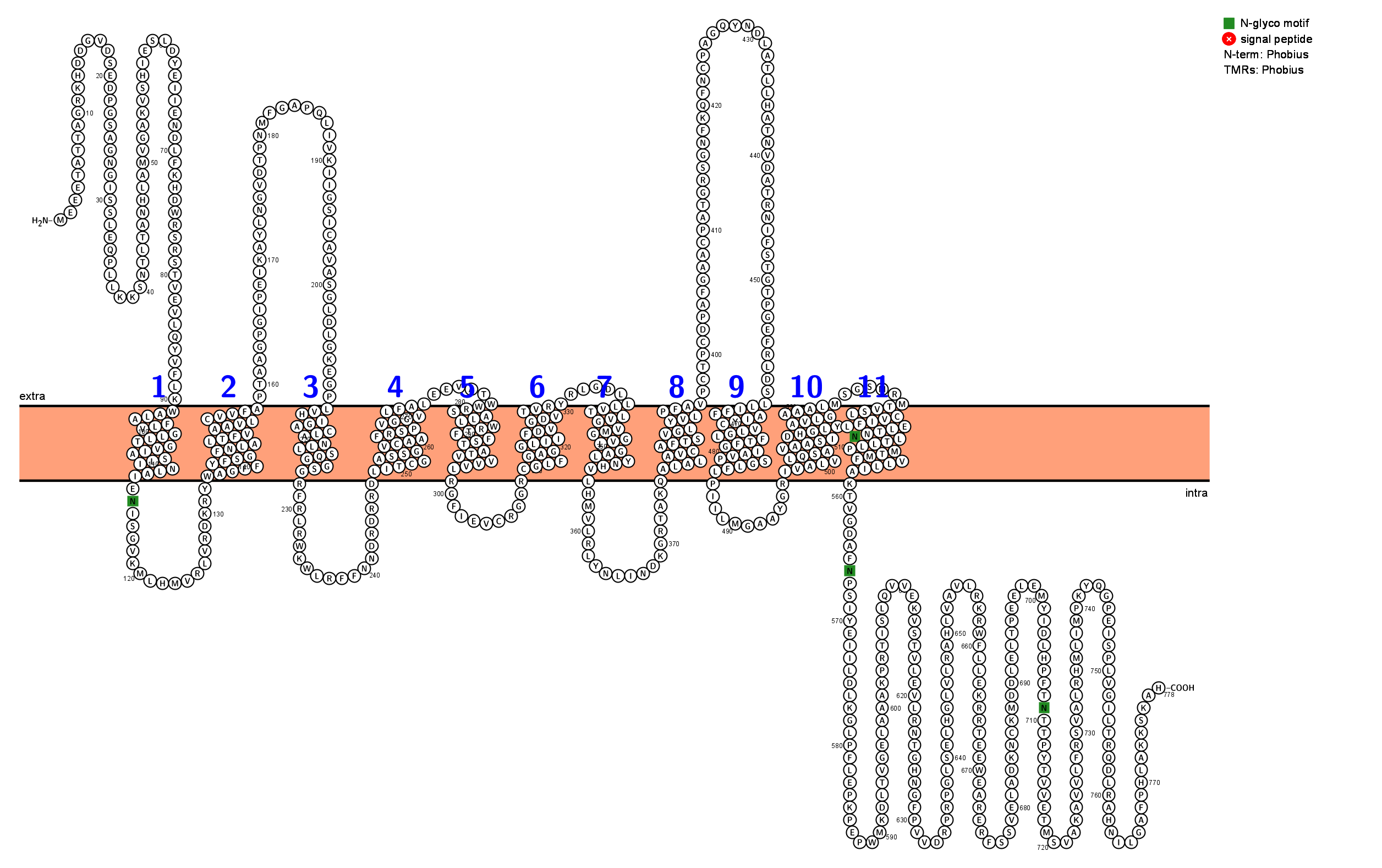

Supplement: Supplementary file 3 [file DataSheet1.ZIP › TaCLC-a-6AS-2.png]

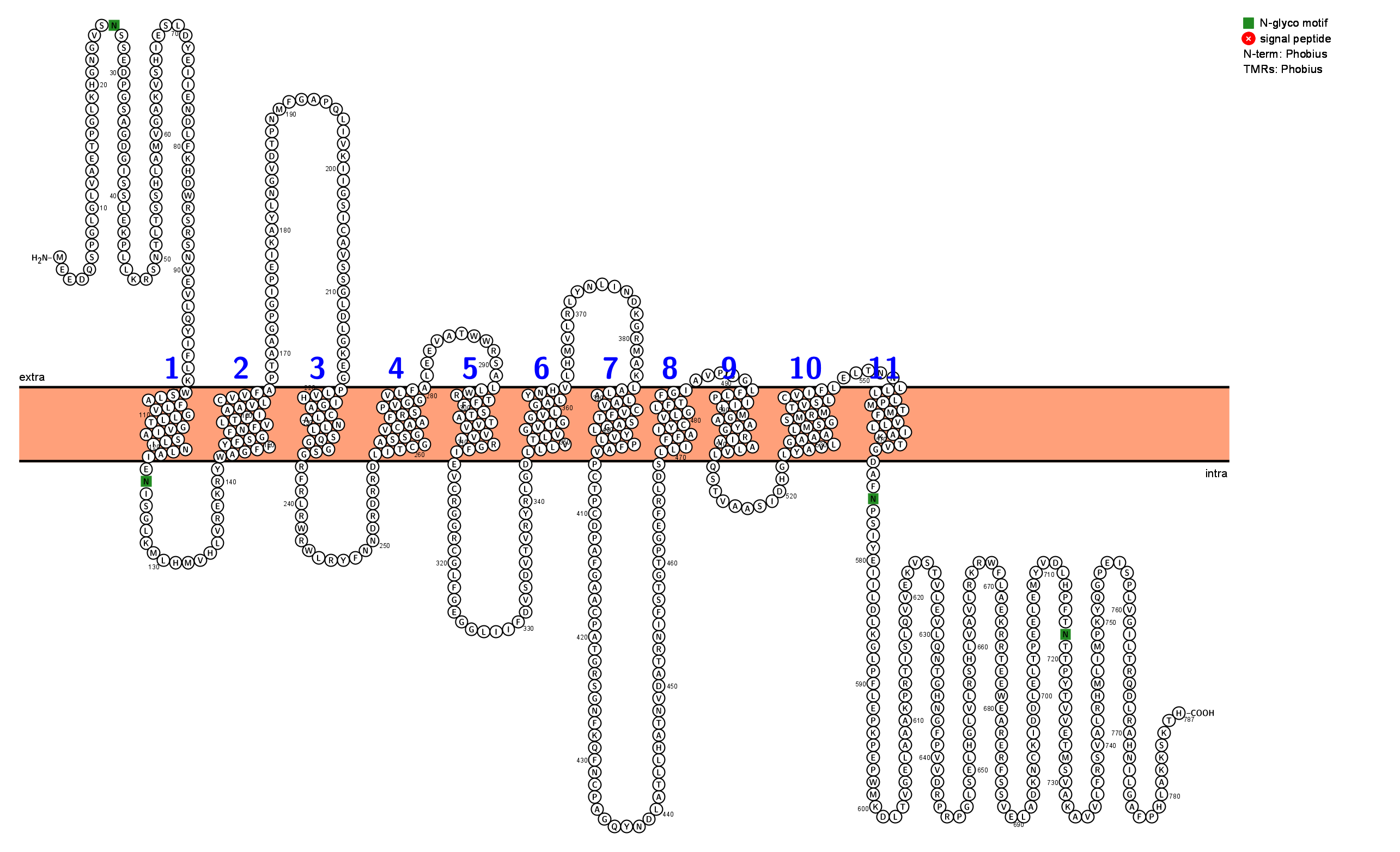

Supplement: Supplementary file 3 [file DataSheet1.ZIP › TaCLC-a-6BS-1.png]

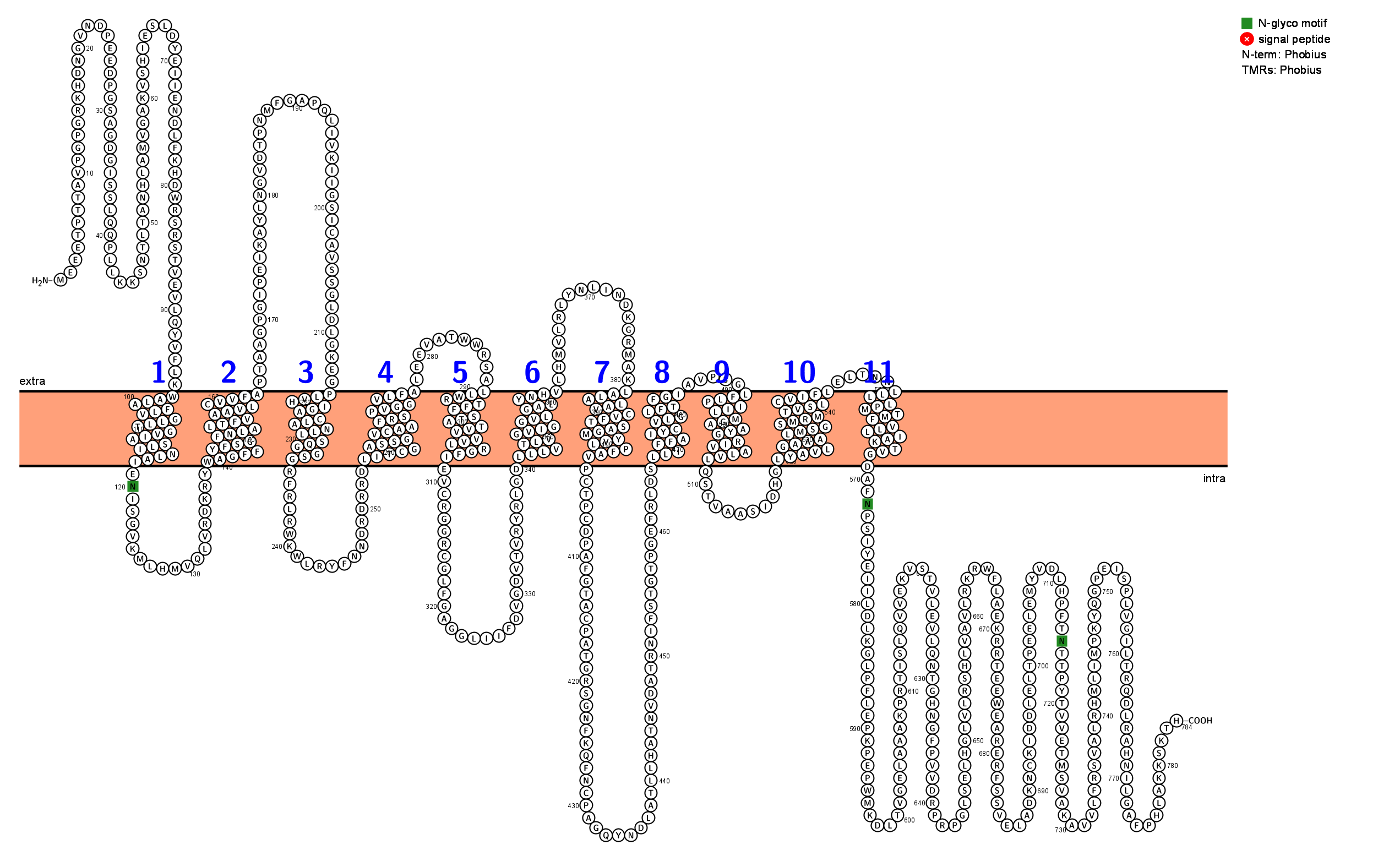

Supplement: Supplementary file 3 [file DataSheet1.ZIP › TaCLC-a-6BS-2.png]

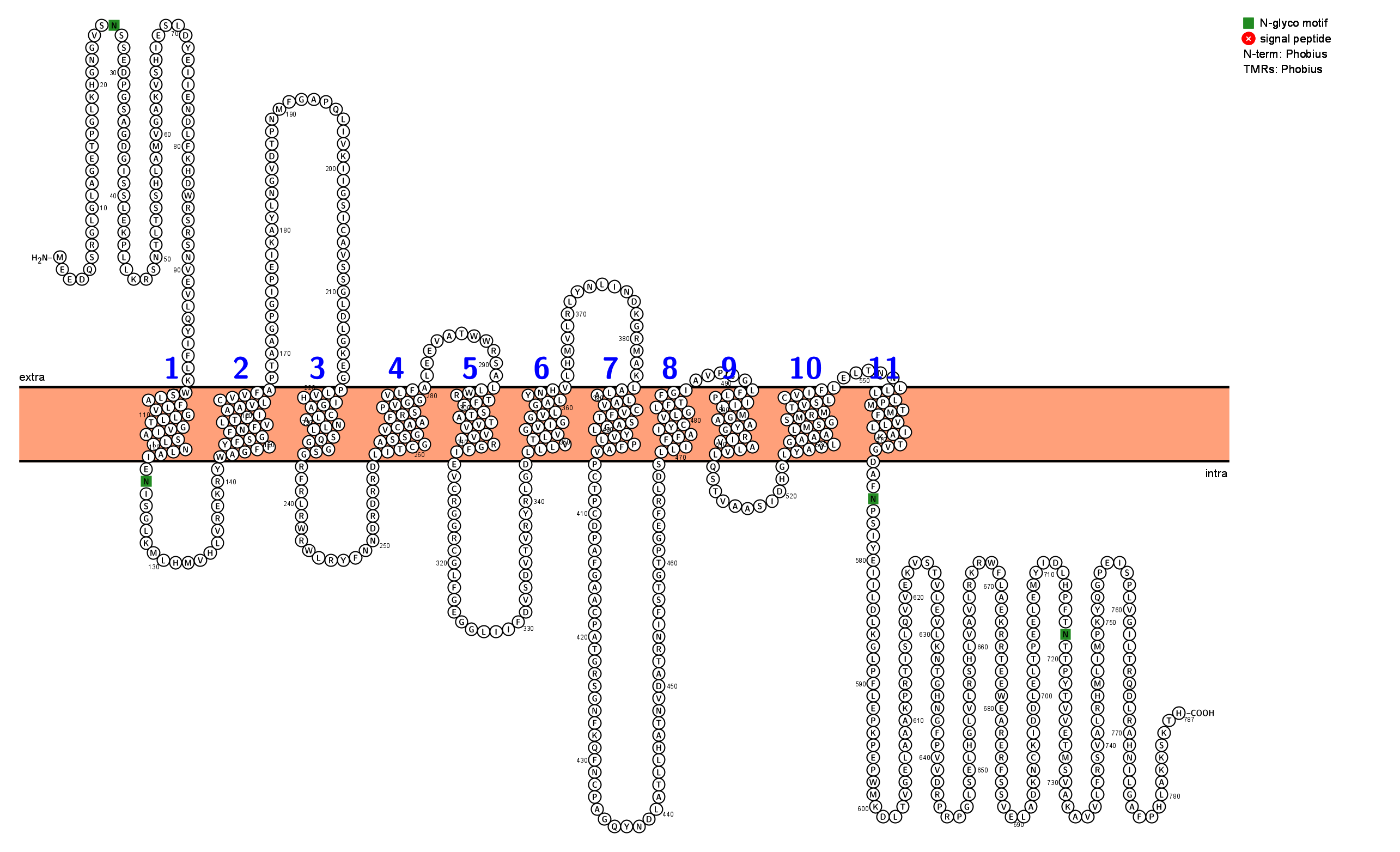

Supplement: Supplementary file 3 [file DataSheet1.ZIP › TaCLC-a-6DS-1.png]

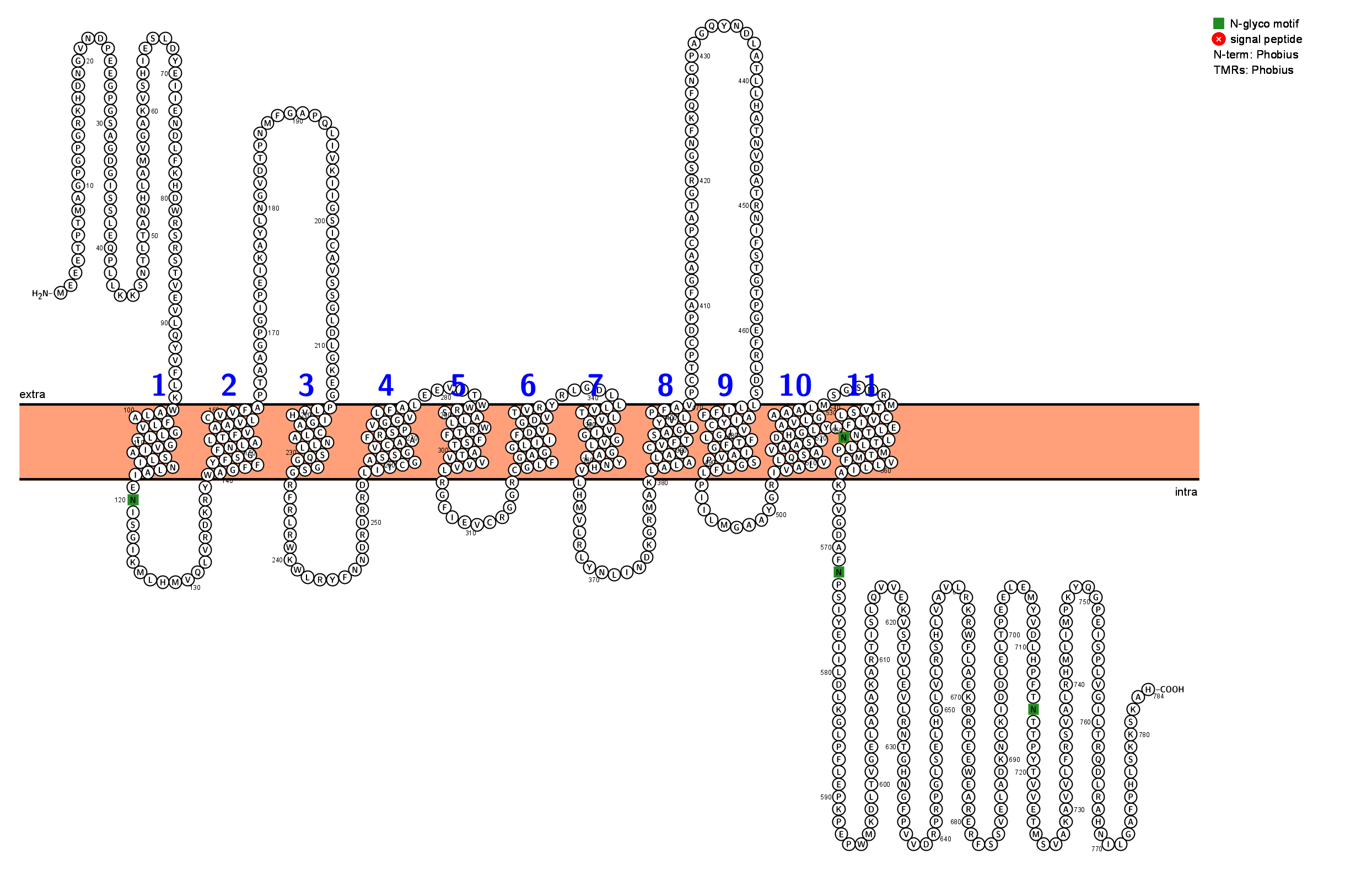

Supplement: Supplementary file 3 [file DataSheet1.ZIP › TaCLC-a-6DS-2.png]

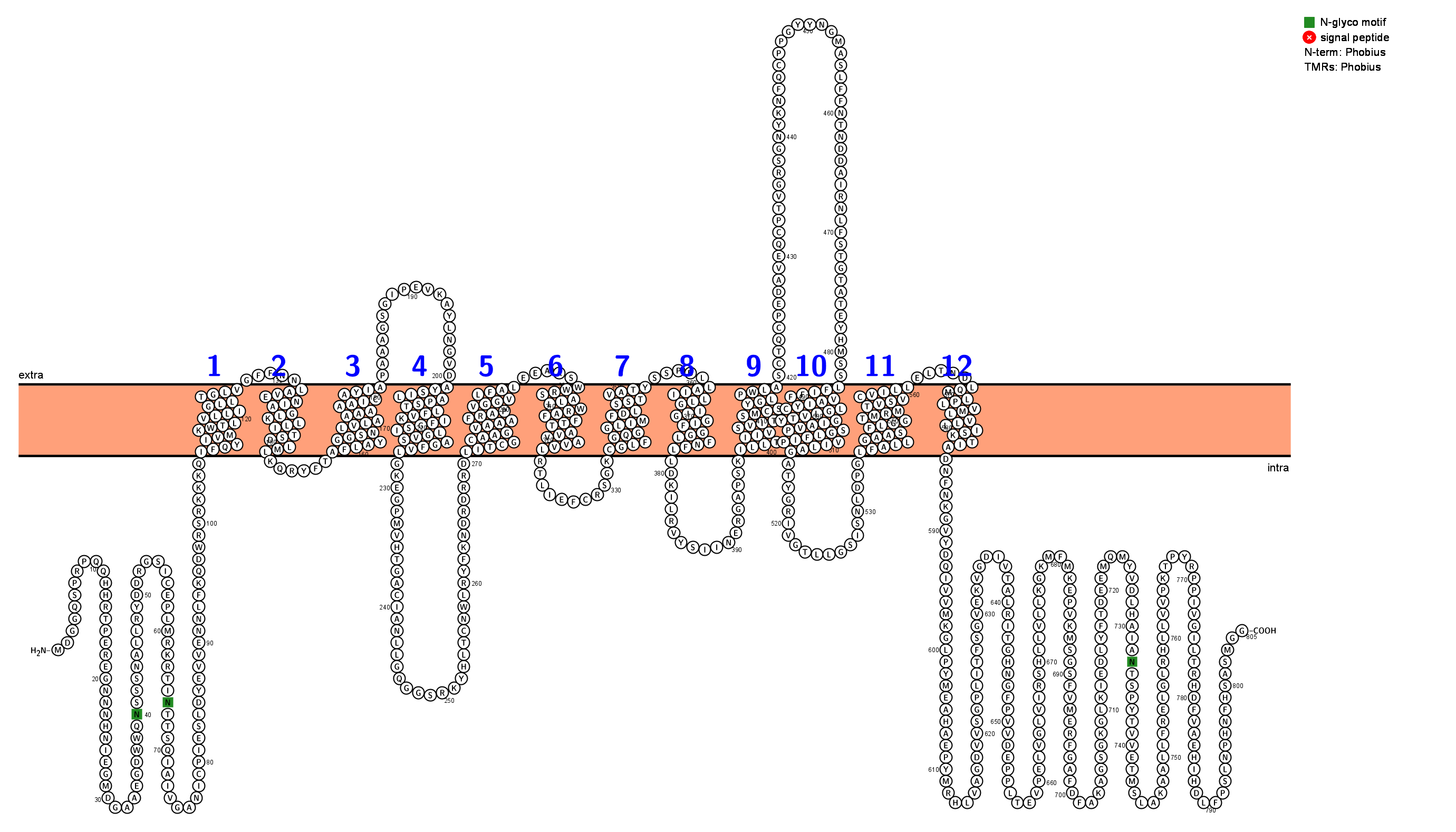

Supplement: Supplementary file 3 [file DataSheet1.ZIP › TaCLC-c1-3AS.png]

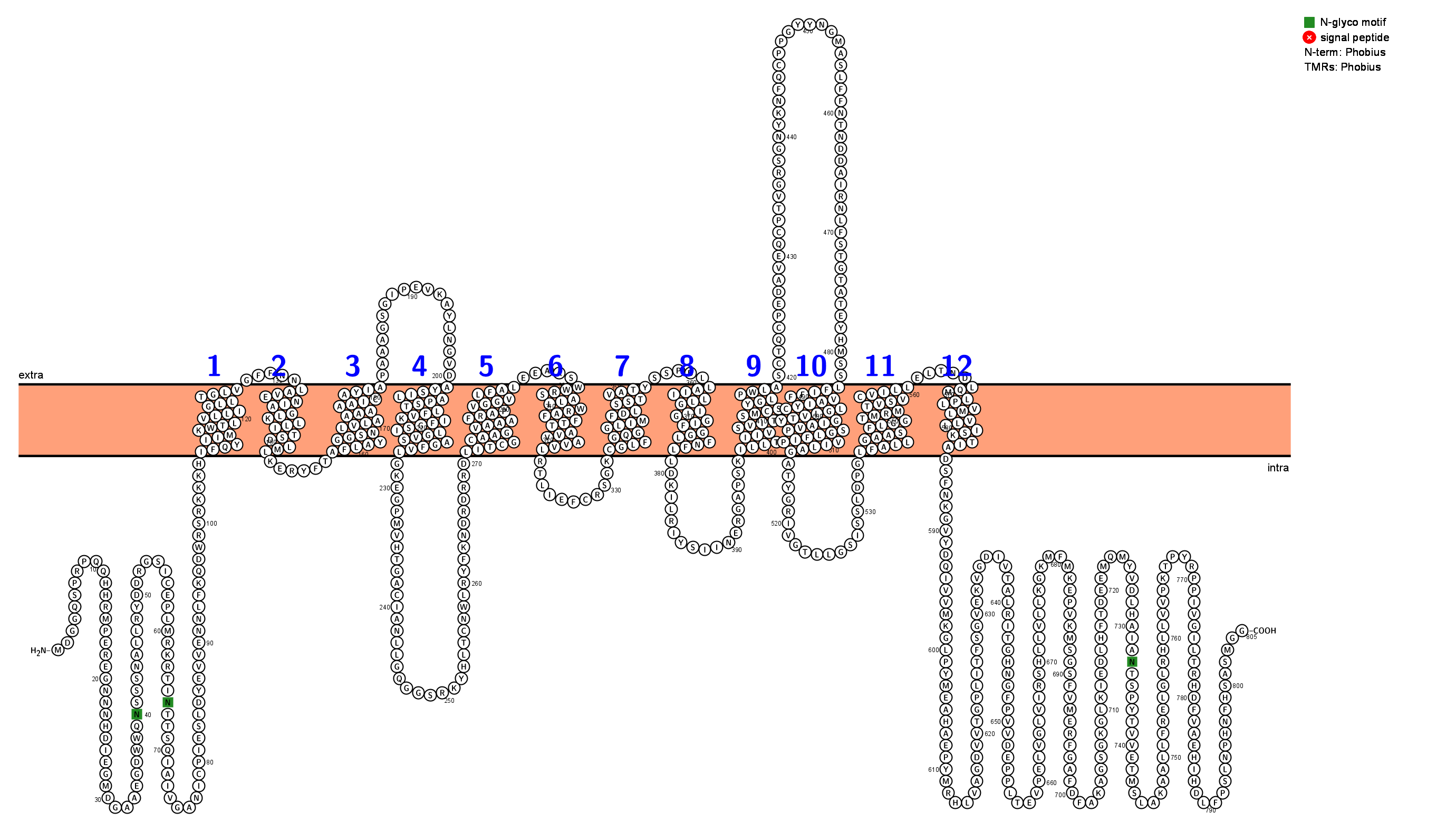

Supplement: Supplementary file 3 [file DataSheet1.ZIP › TaCLC-c1-3DS-1.png]

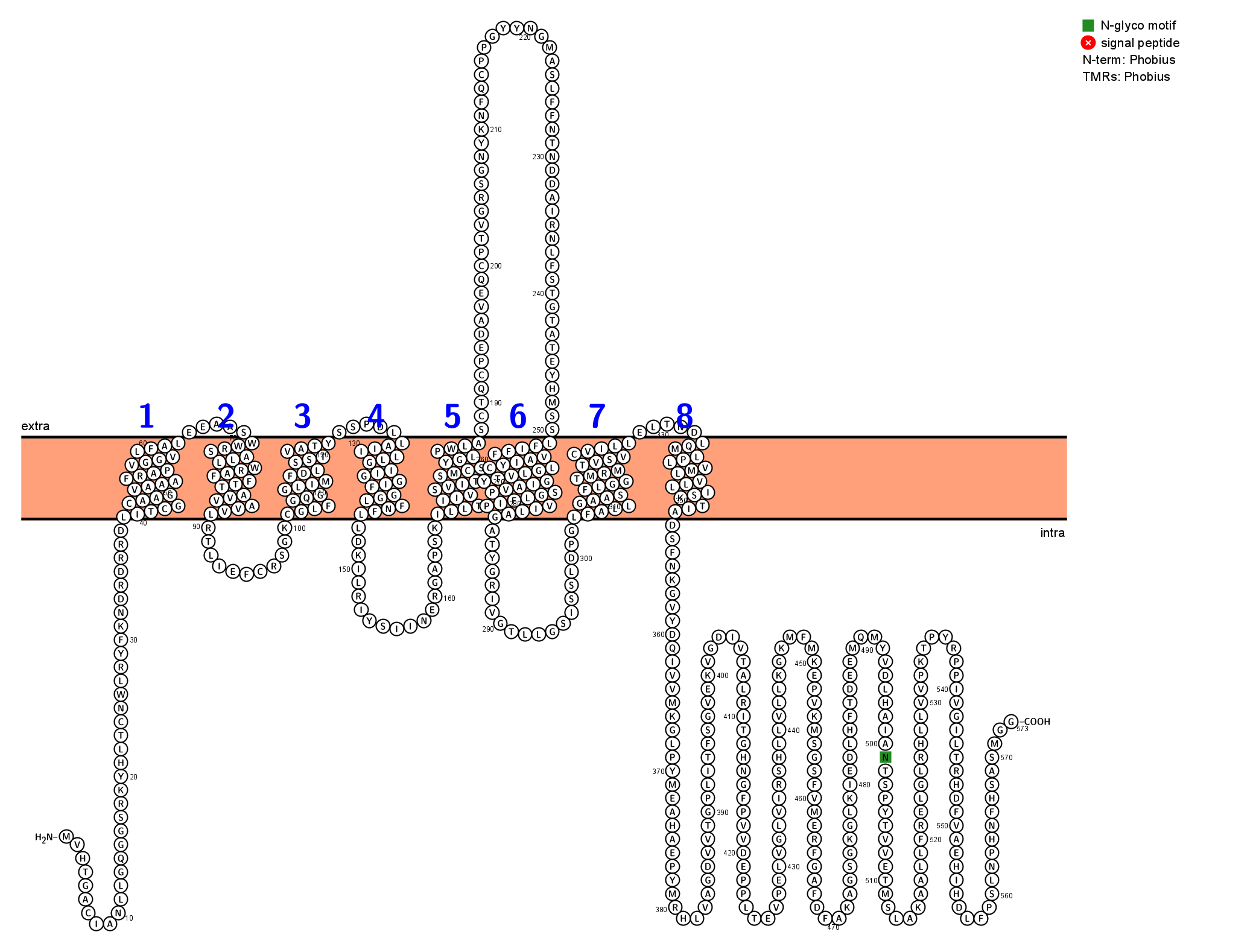

Supplement: Supplementary file 3 [file DataSheet1.ZIP › TaCLC-c1-3DS-2.png]

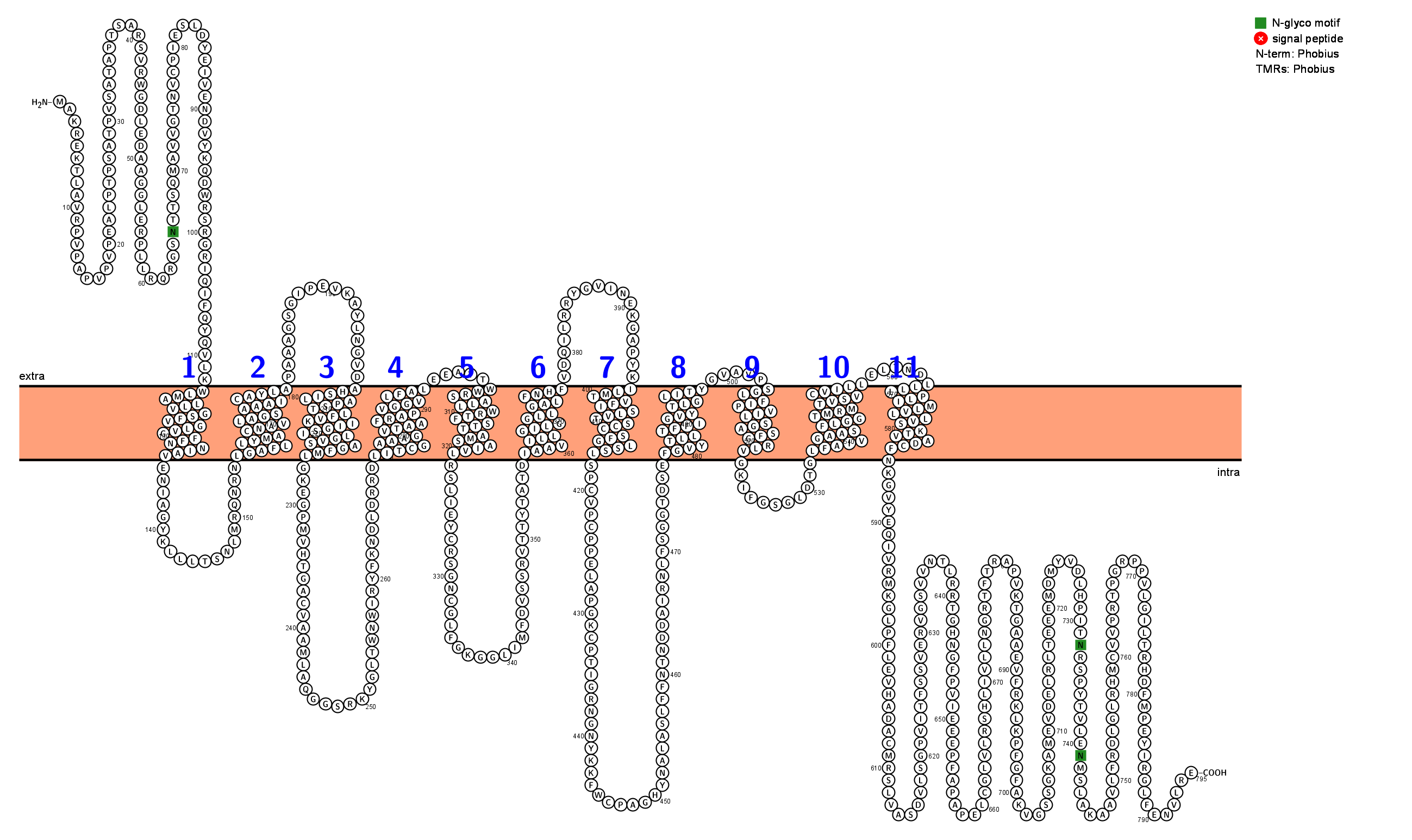

Supplement: Supplementary file 3 [file DataSheet1.ZIP › TaCLC-c2-3AL.png]

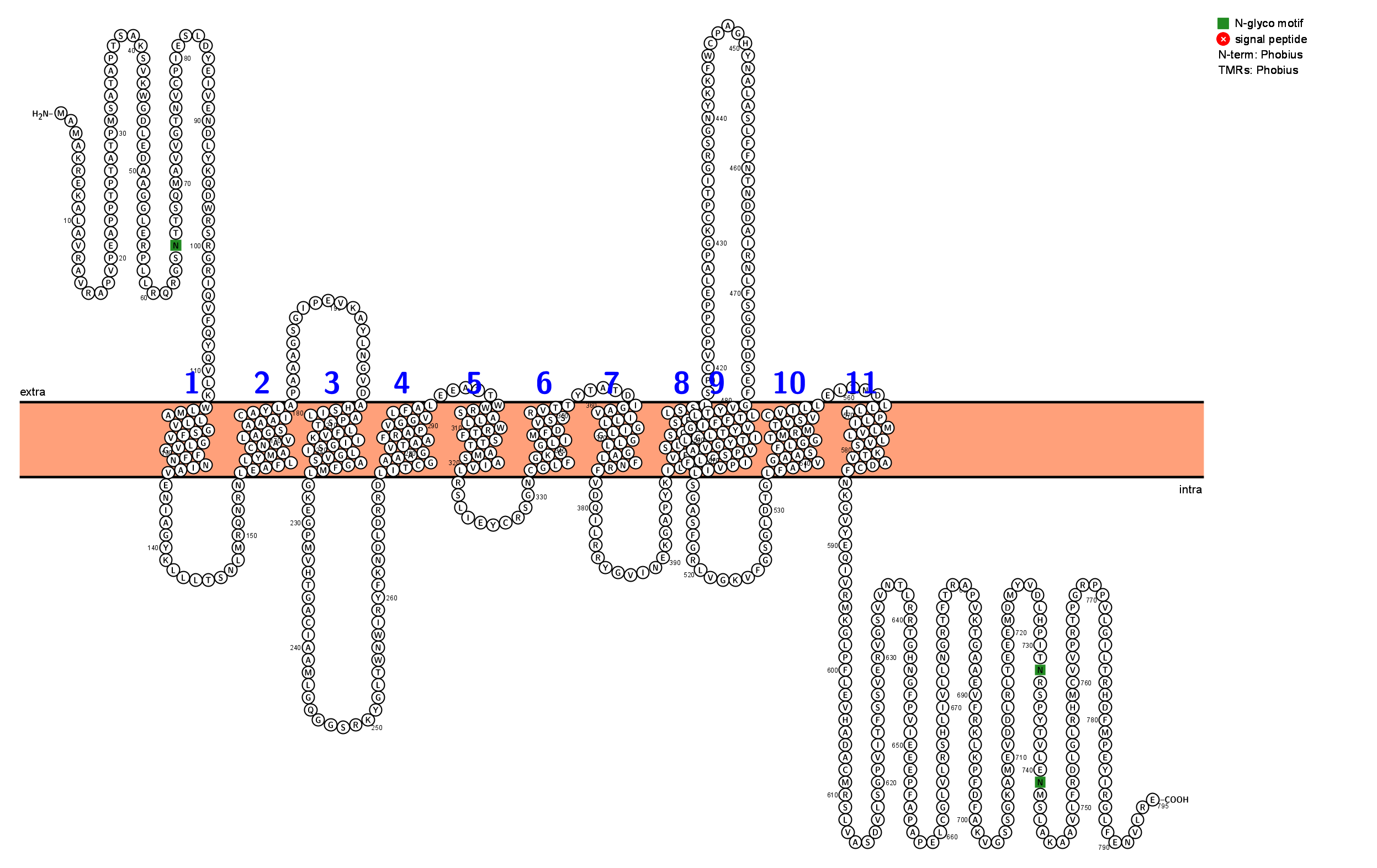

Supplement: Supplementary file 3 [file DataSheet1.ZIP › TaCLC-c2-3B.png]

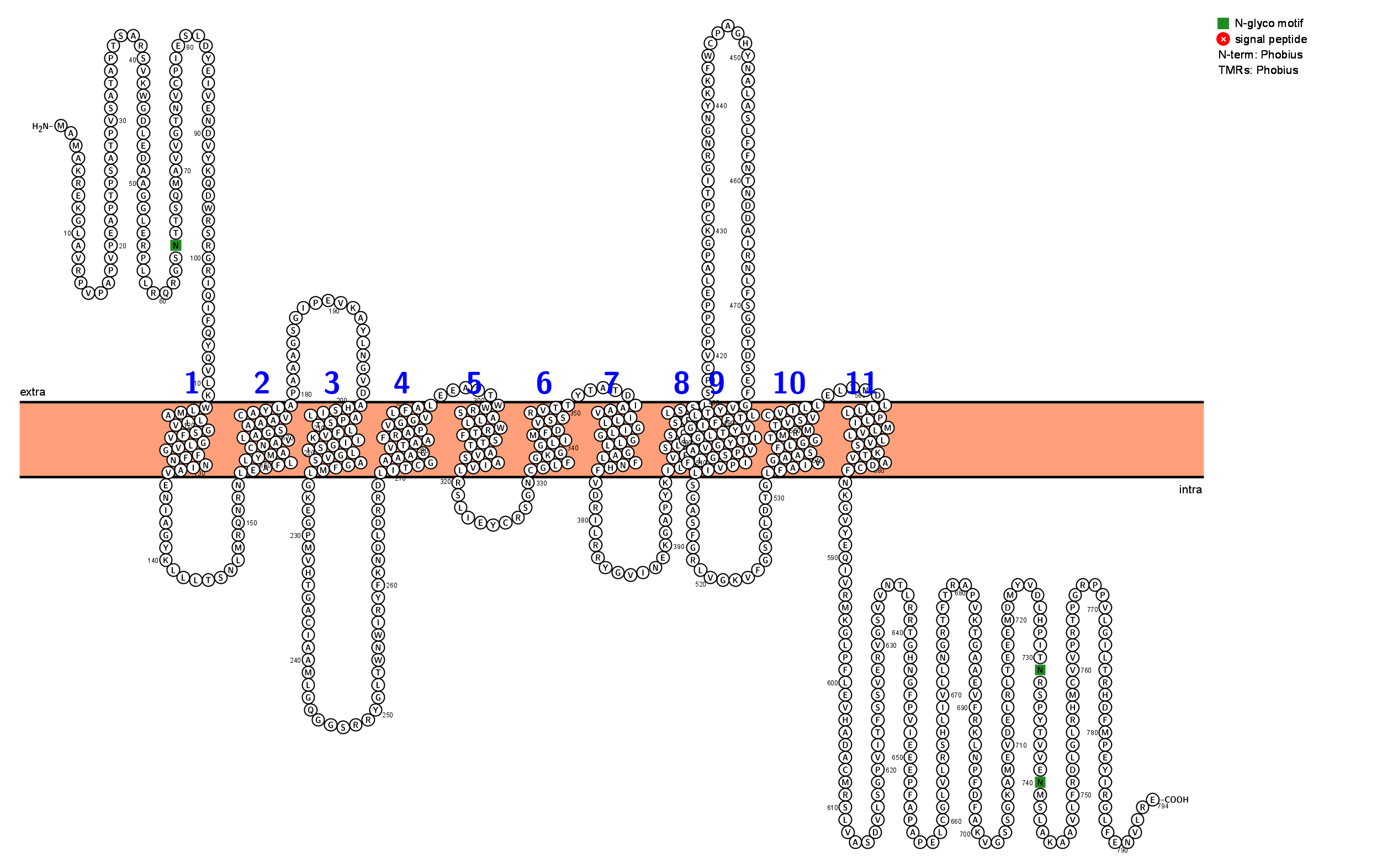

Supplement: Supplementary file 3 [file DataSheet1.ZIP › TaCLC-c2-3DL.png]

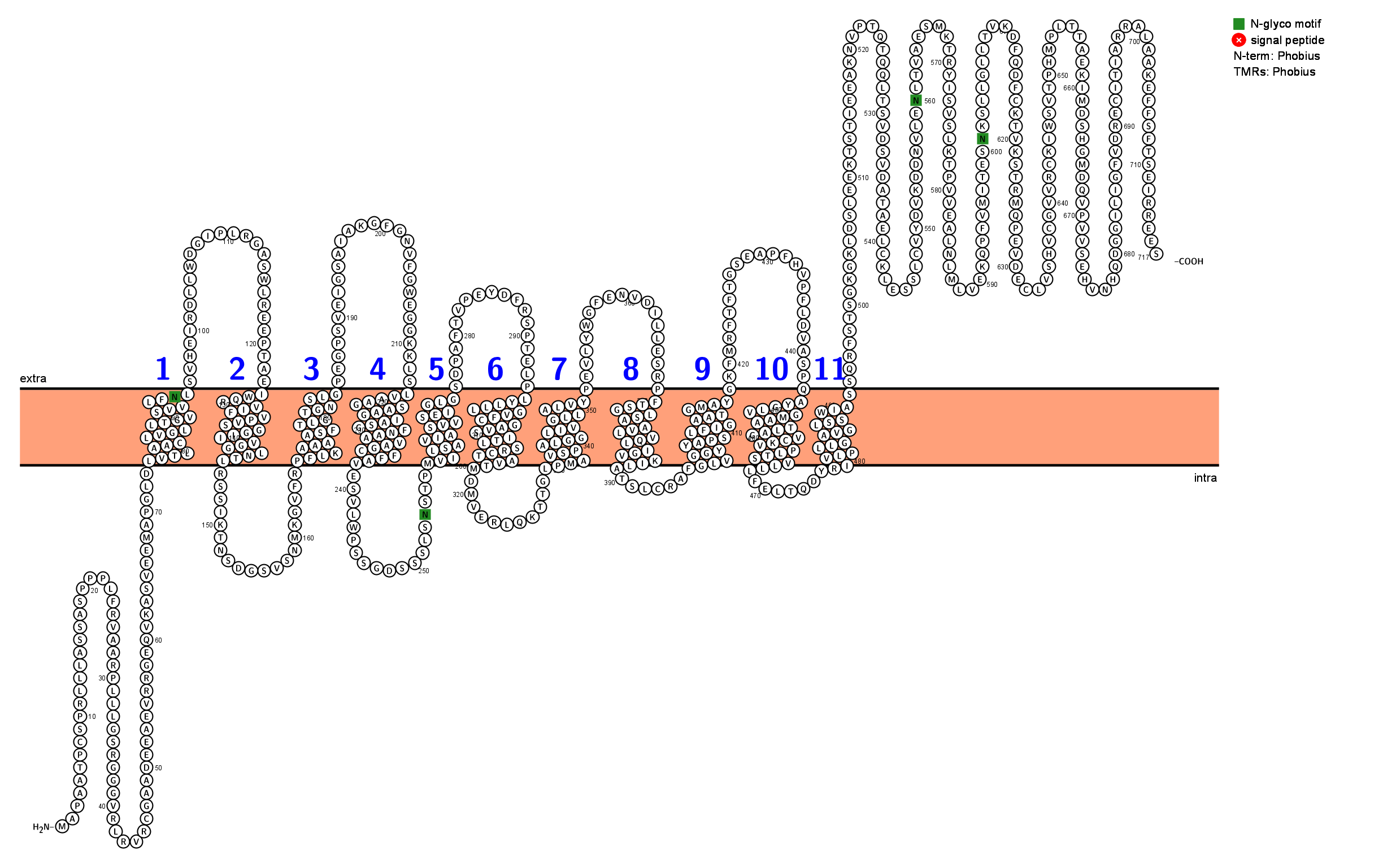

Supplement: Supplementary file 3 [file DataSheet1.ZIP › TaCLC-e-3AL.png]

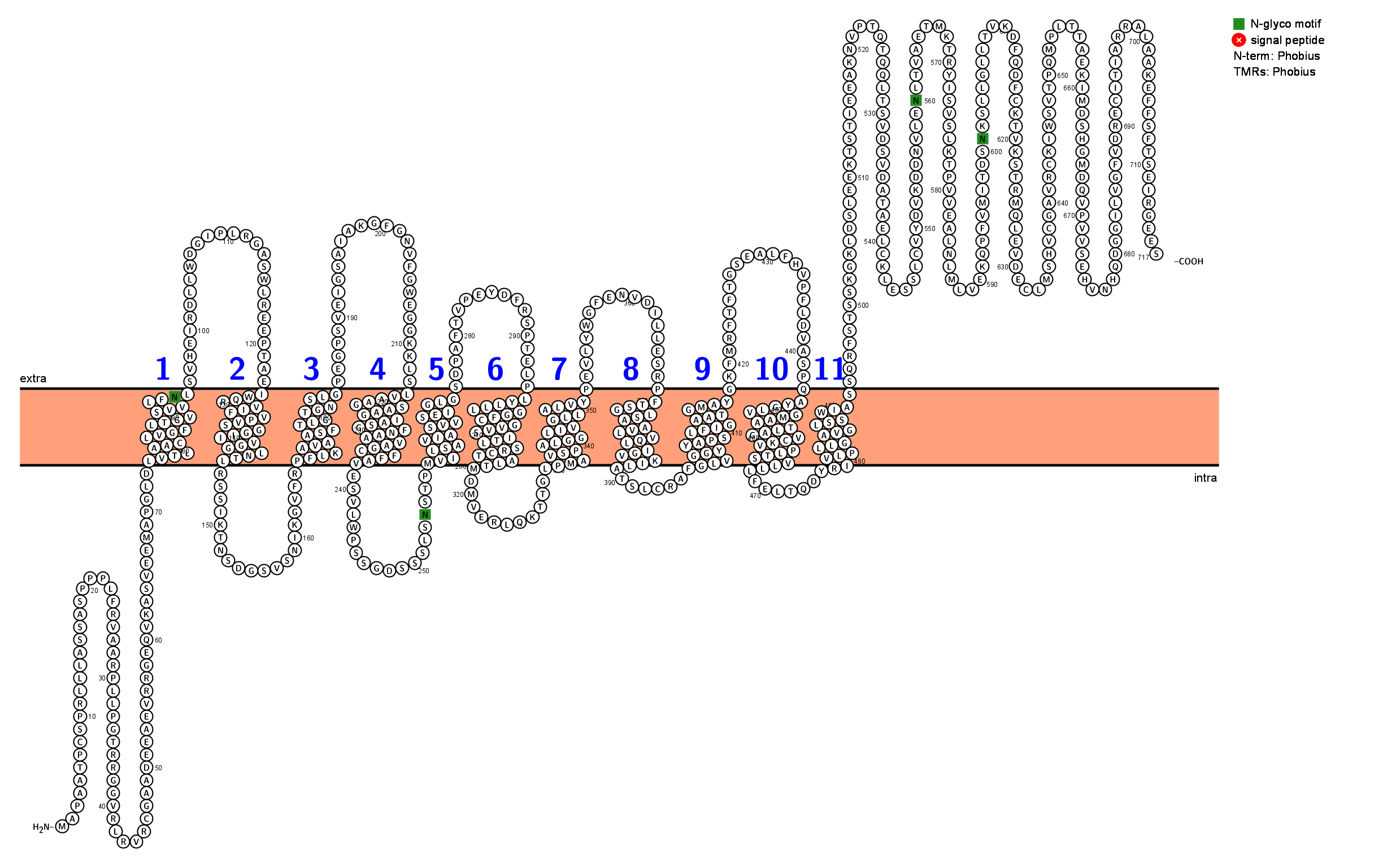

Supplement: Supplementary file 3 [file DataSheet1.ZIP › TaCLC-e-3B.png]

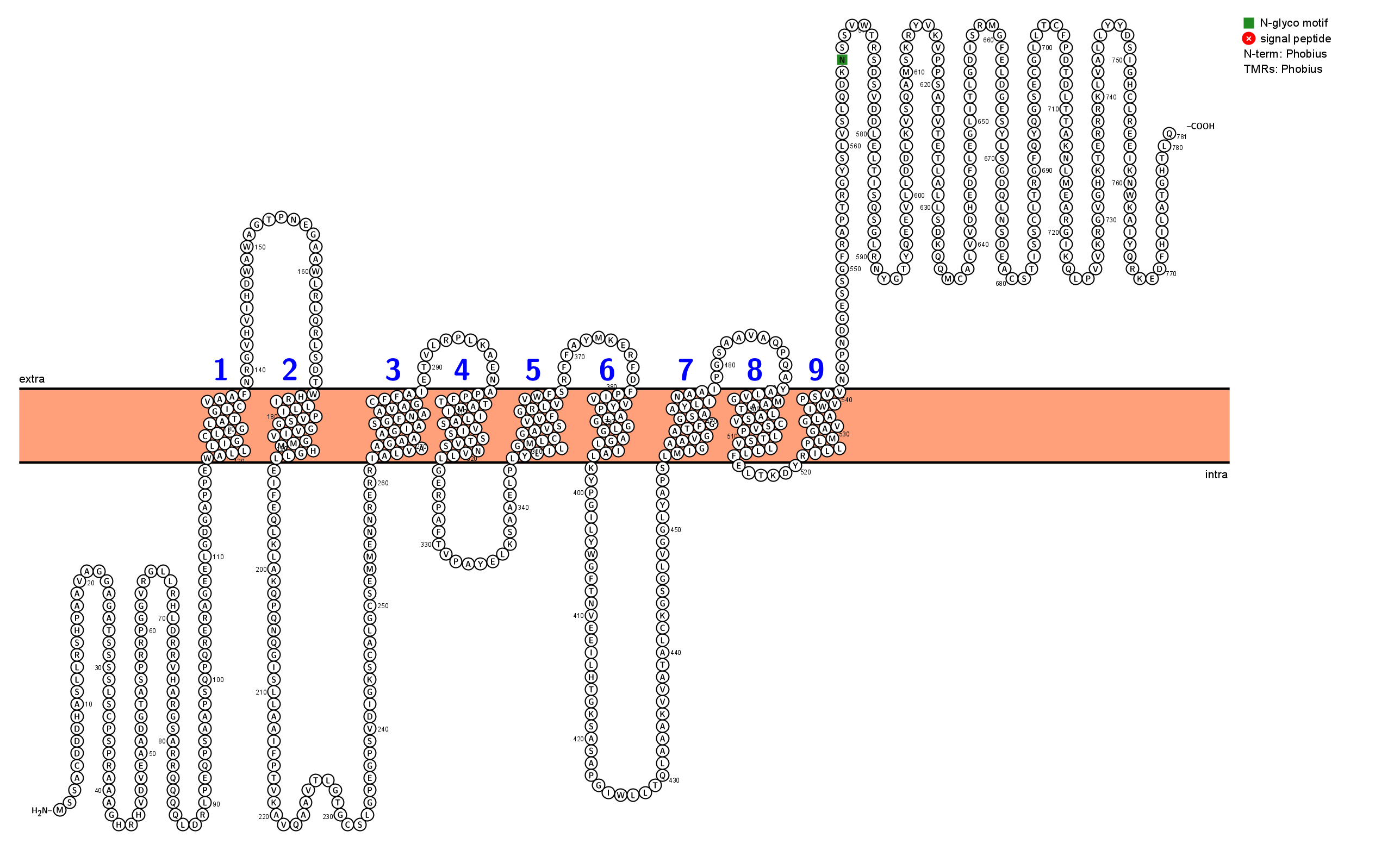

Supplement: Supplementary file 3 [file DataSheet1.ZIP › TaCLC-f1-6AL.png]

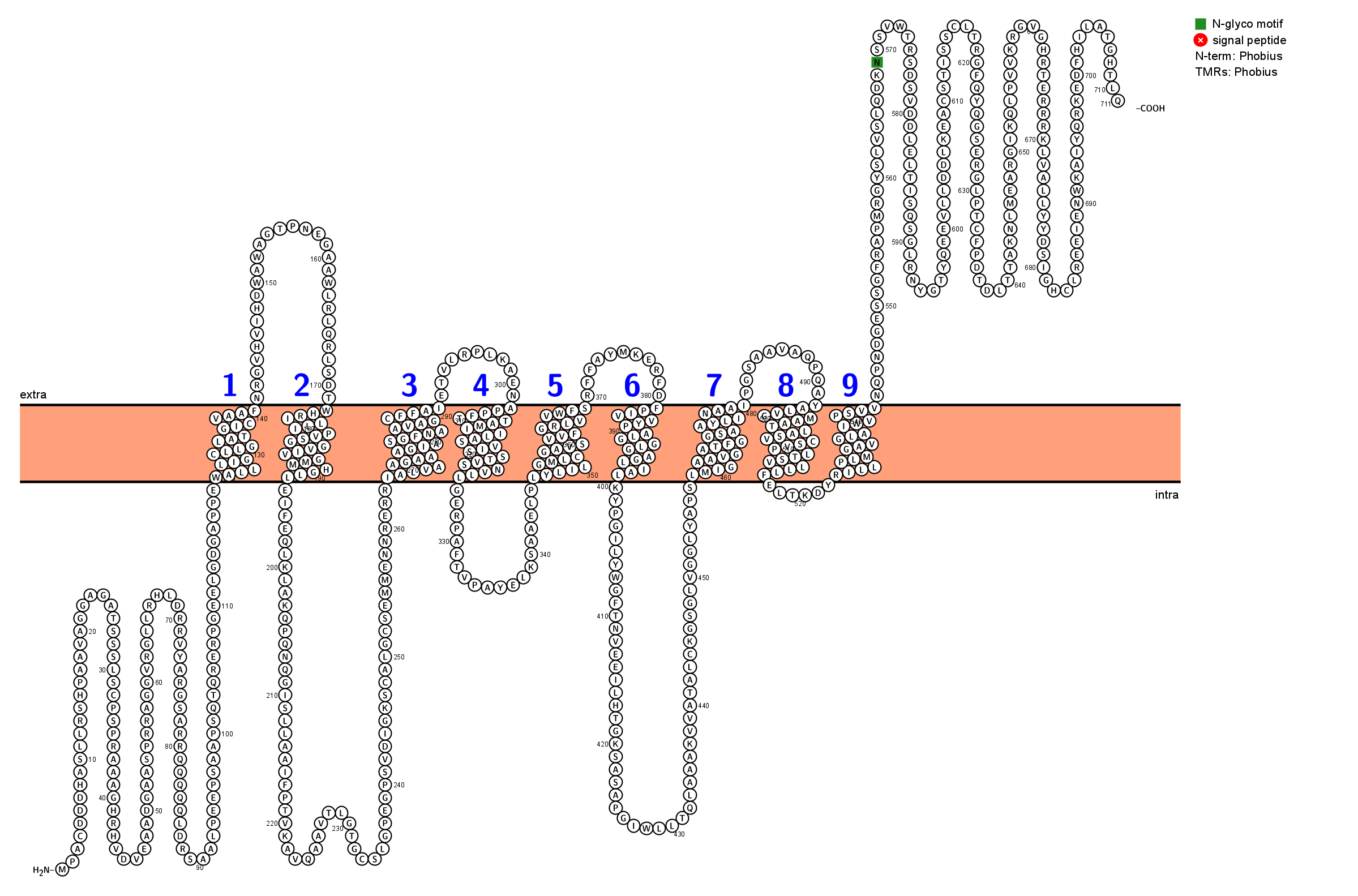

Supplement: Supplementary file 3 [file DataSheet1.ZIP › TaCLC-f1-6BL.png]

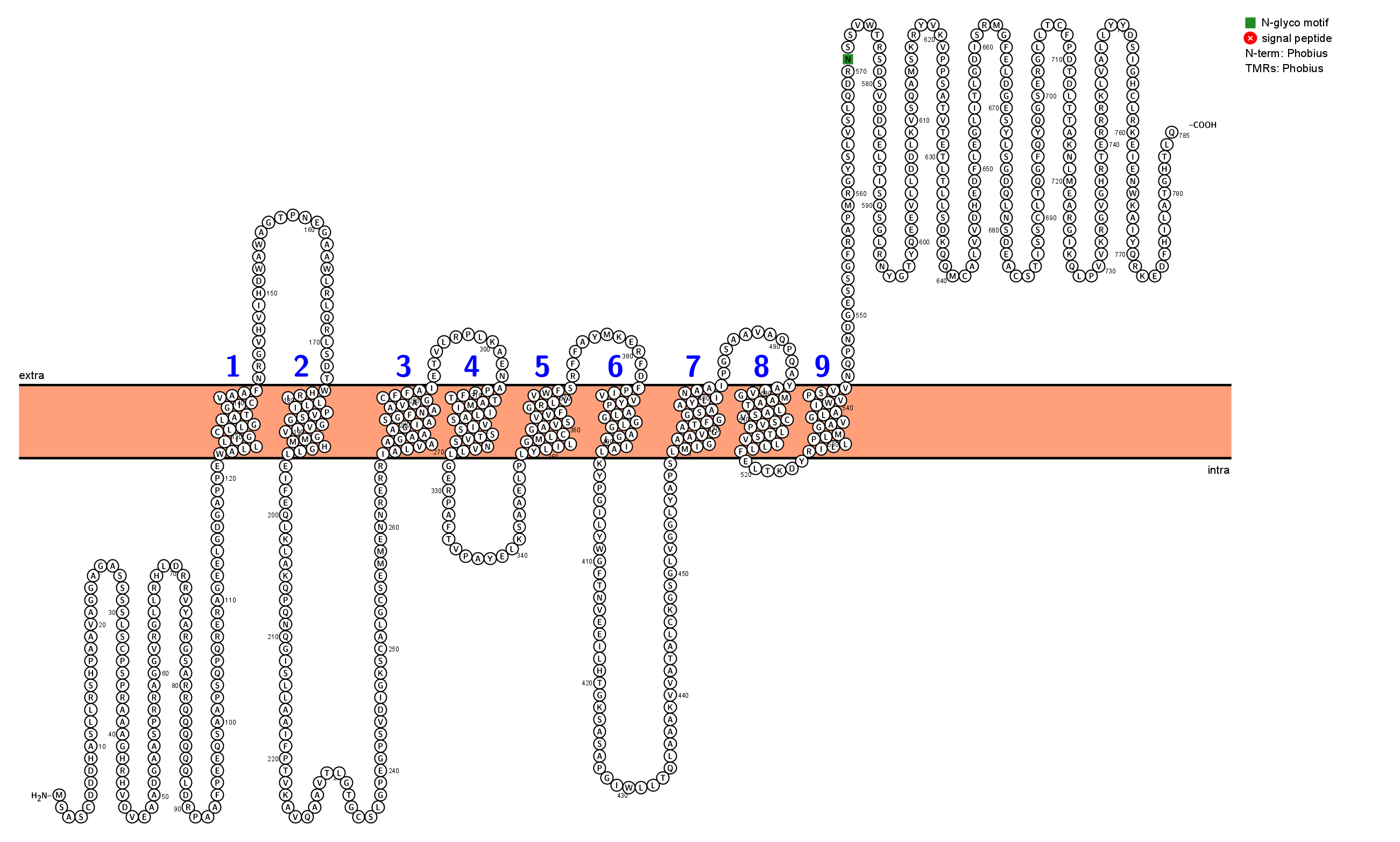

Supplement: Supplementary file 3 [file DataSheet1.ZIP › TaCLC-f1-6DL.png]

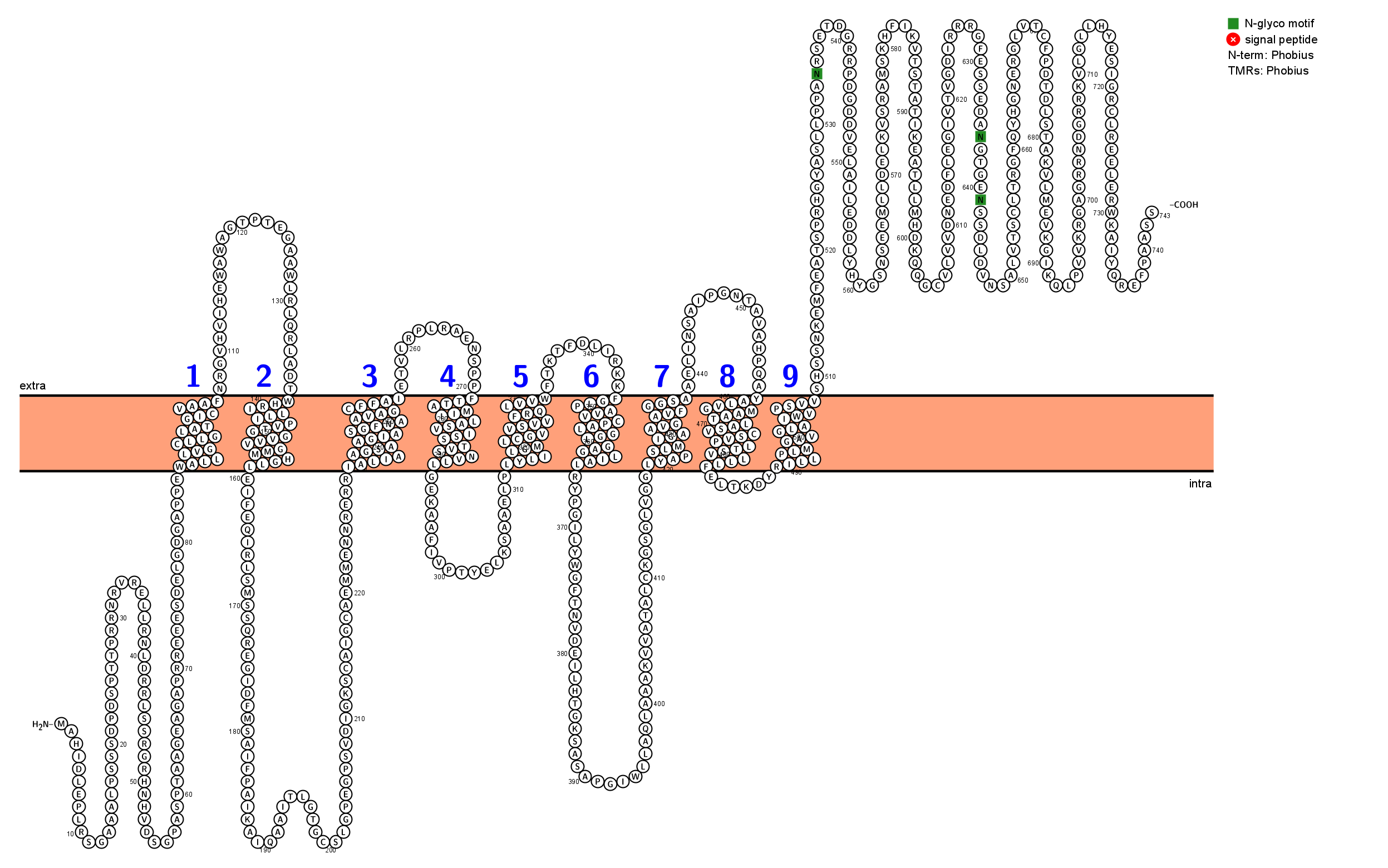

Supplement: Supplementary file 3 [file DataSheet1.ZIP › TaCLC-f2-7AS.png]

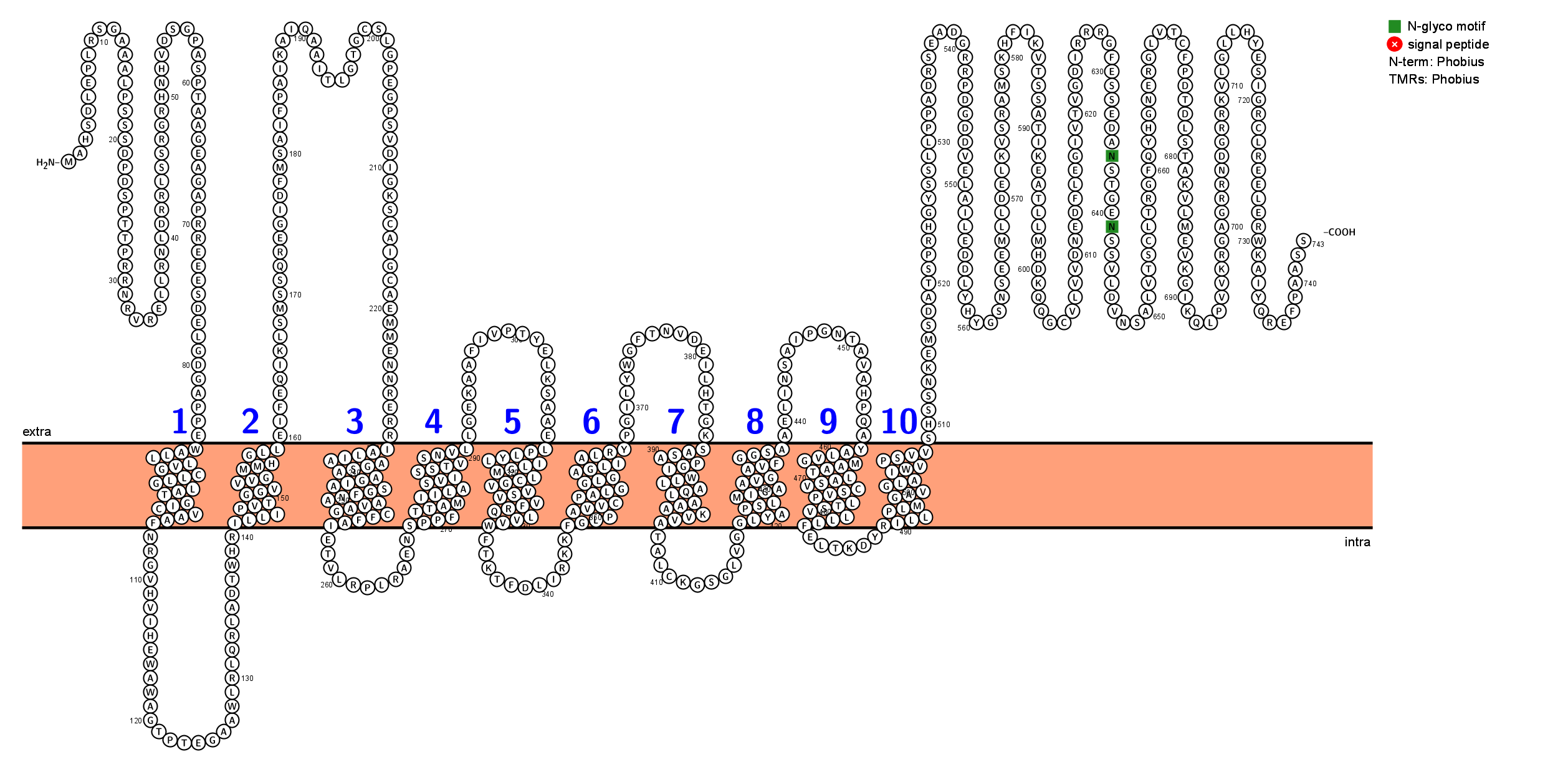

Supplement: Supplementary file 3 [file DataSheet1.ZIP › TaCLC-f2-7BS.png]

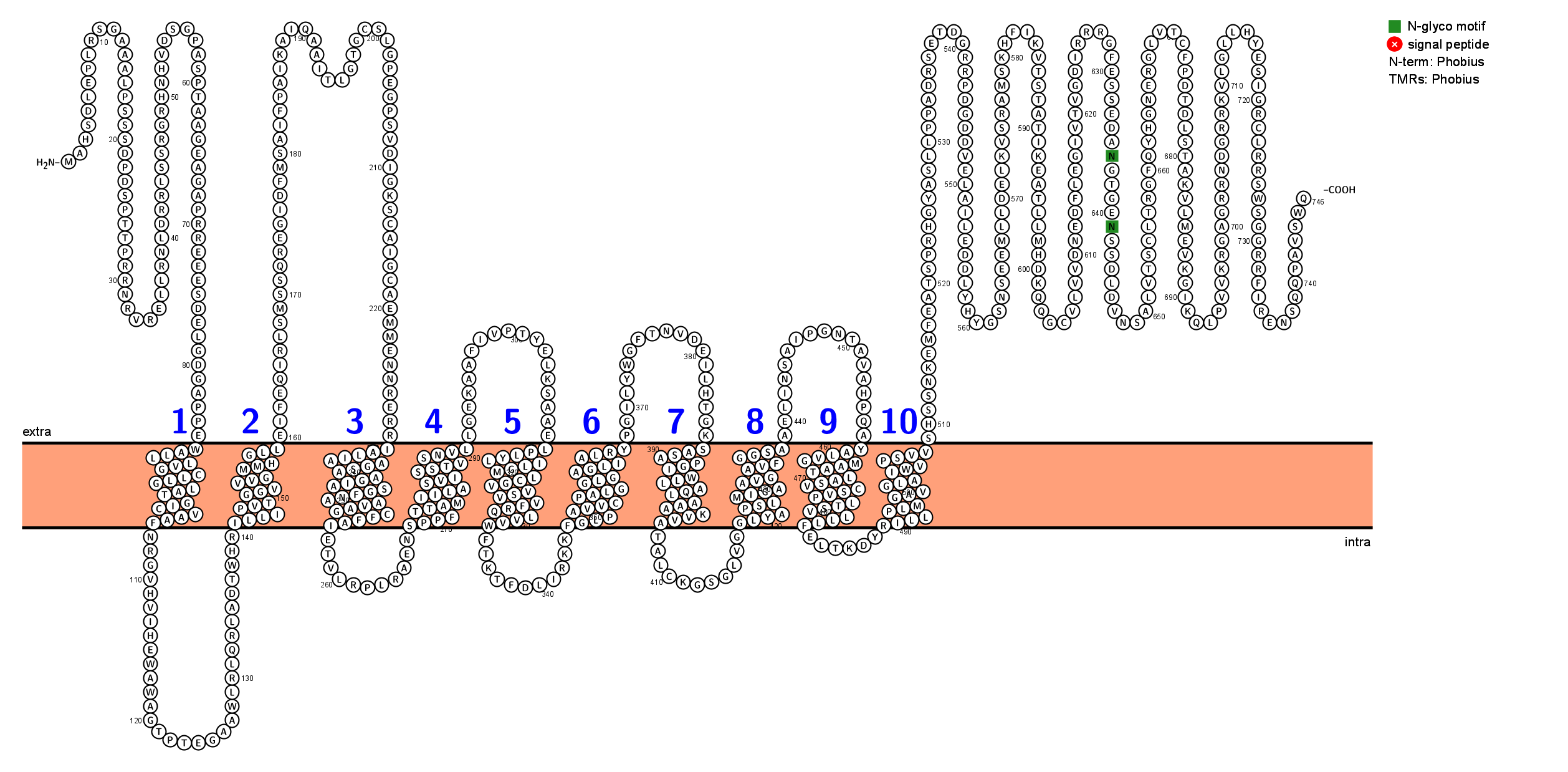

Supplement: Supplementary file 3 [file DataSheet1.ZIP › TaCLC-f2-7DS.png]

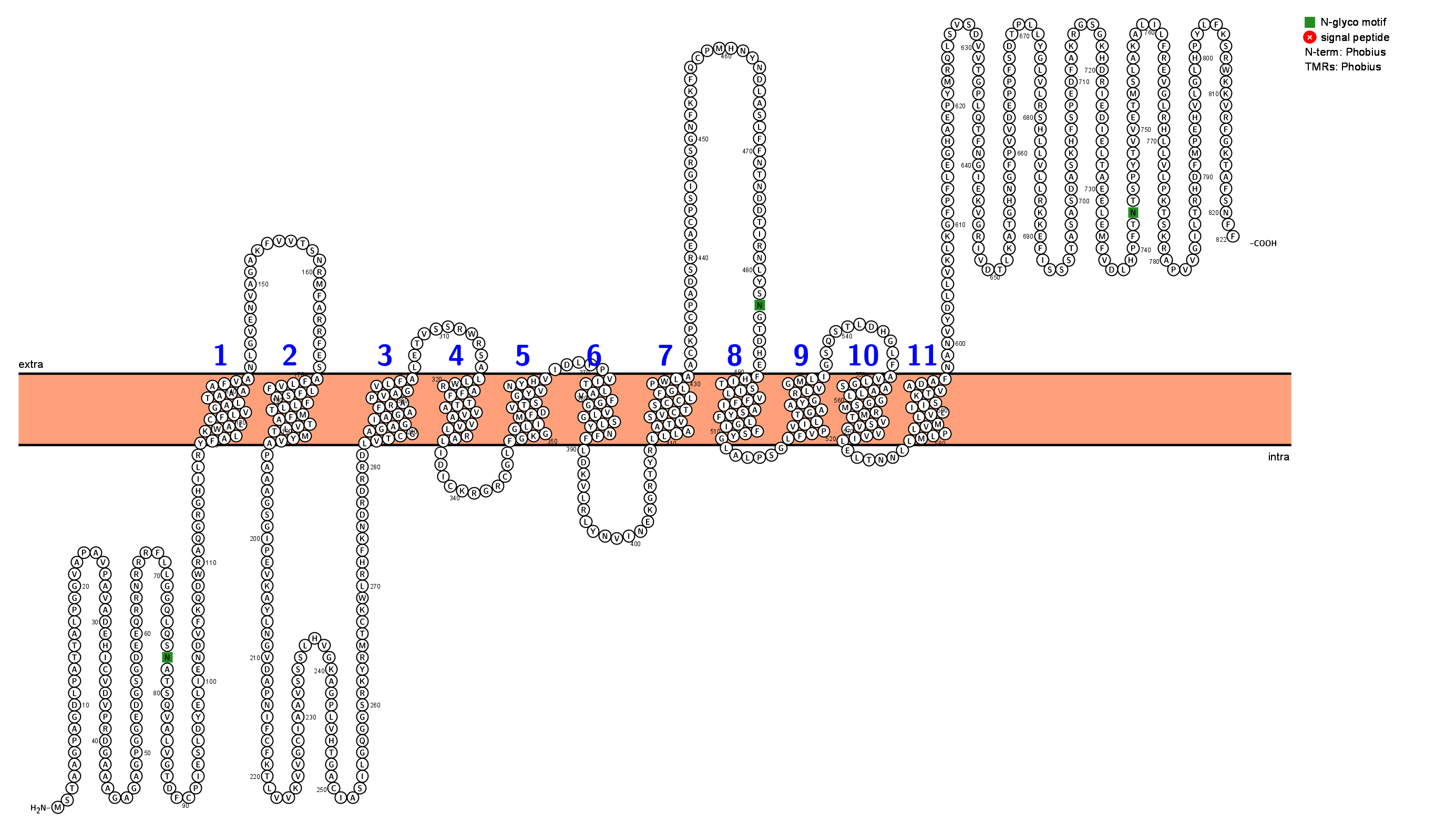

Supplement: Supplementary file 3 [file DataSheet1.ZIP › TaCLC-g2-2AL.png]

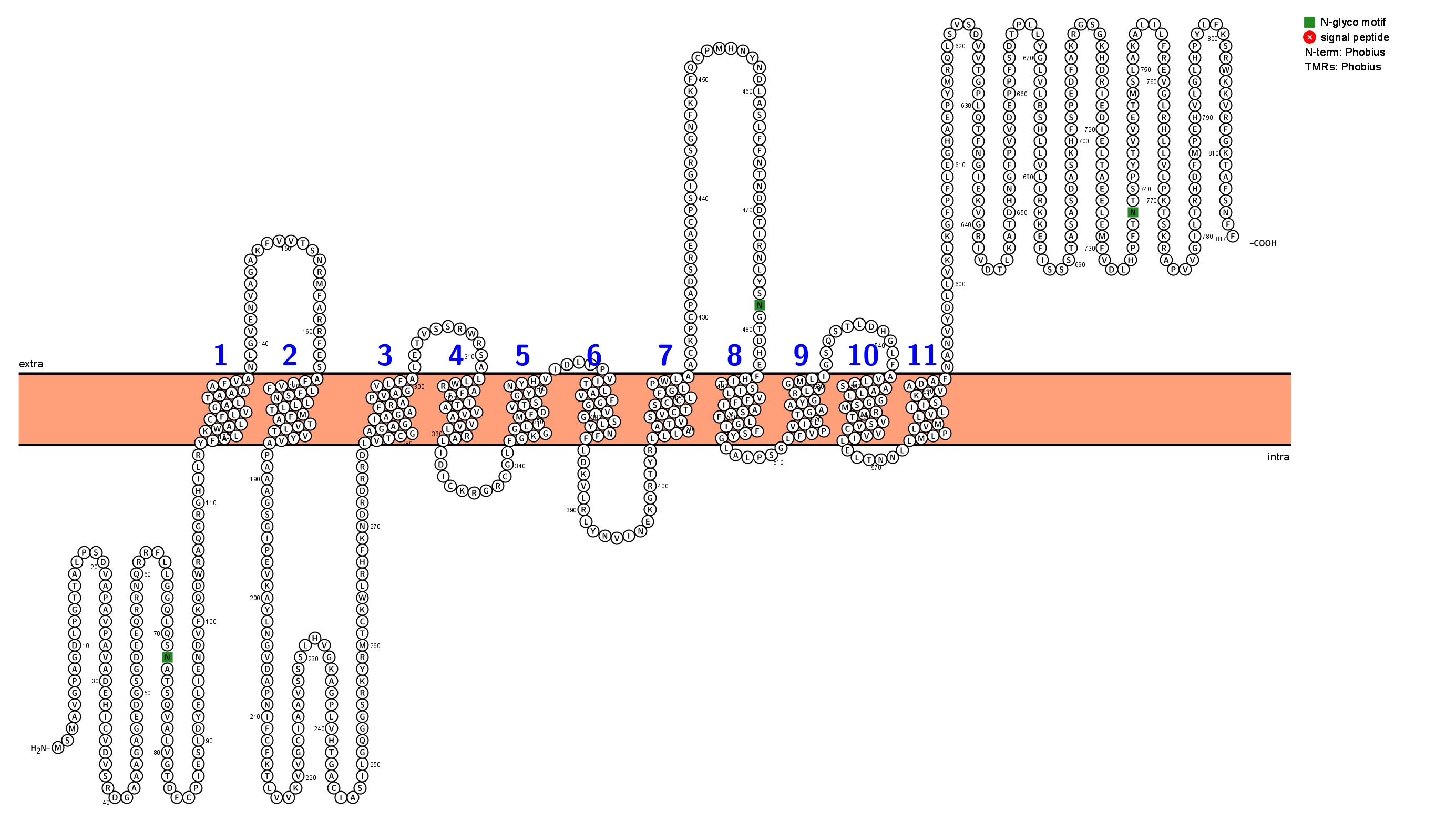

Supplement: Supplementary file 3 [file DataSheet1.ZIP › TaCLC-g2-2BL.png]

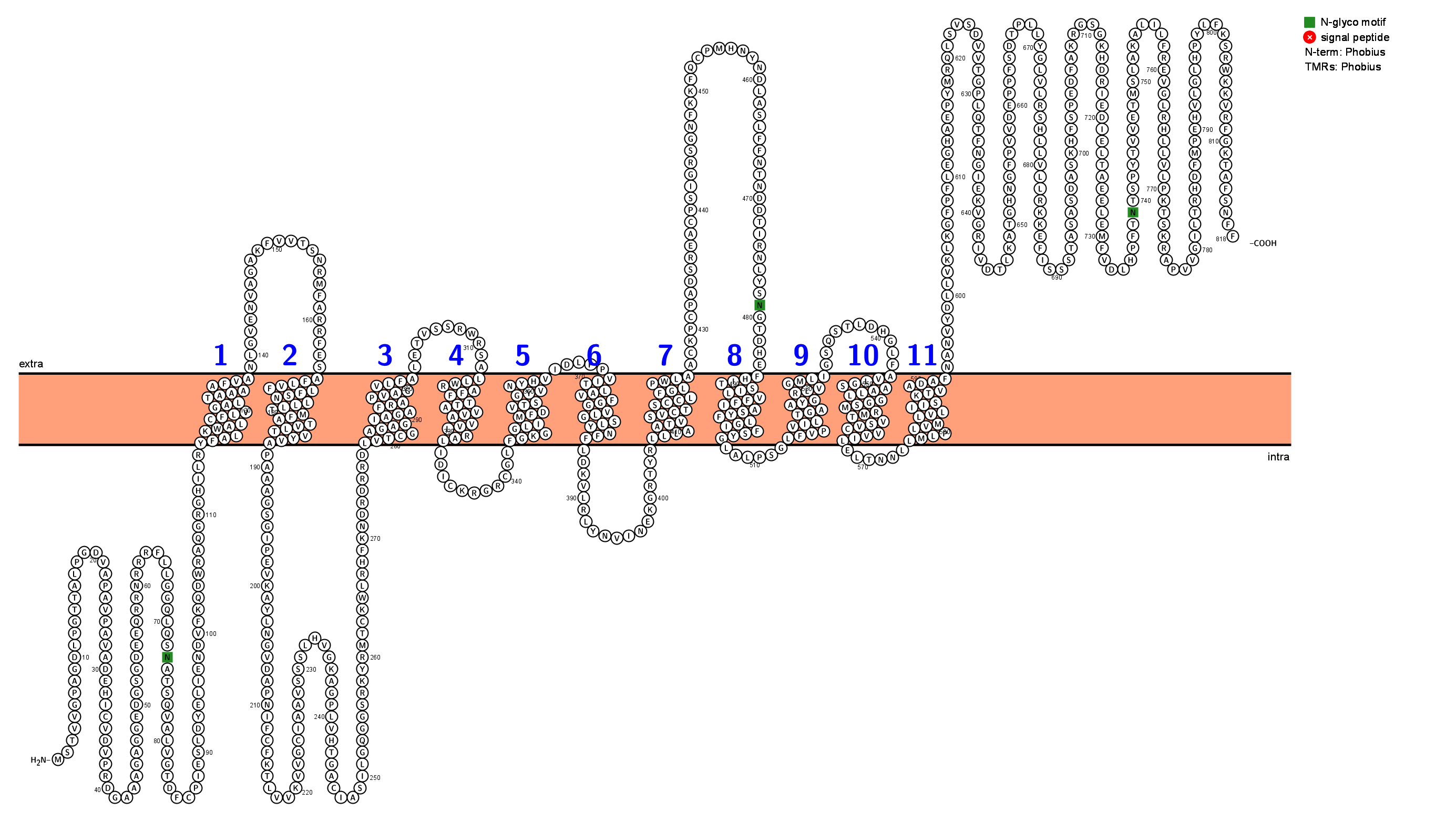

Supplement: Supplementary file 3 [file DataSheet1.ZIP › TaCLC-g2-2DL.png]

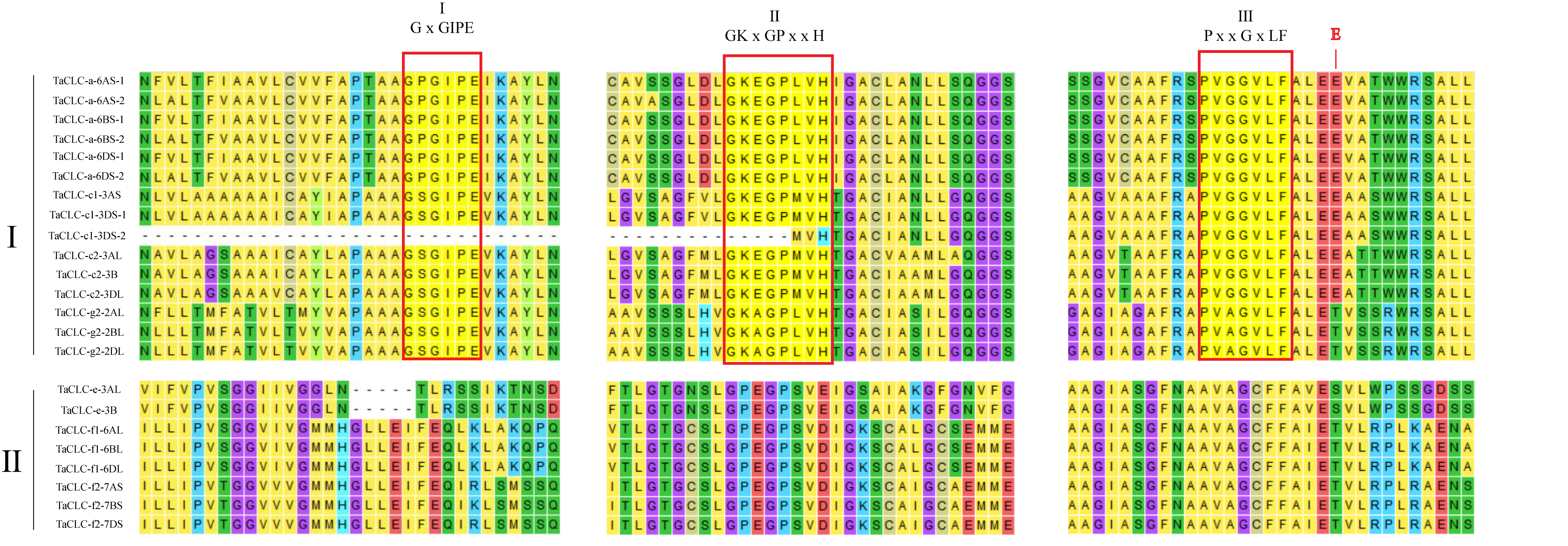

Supplement: Supplementary file 4 [file Image2.tif]

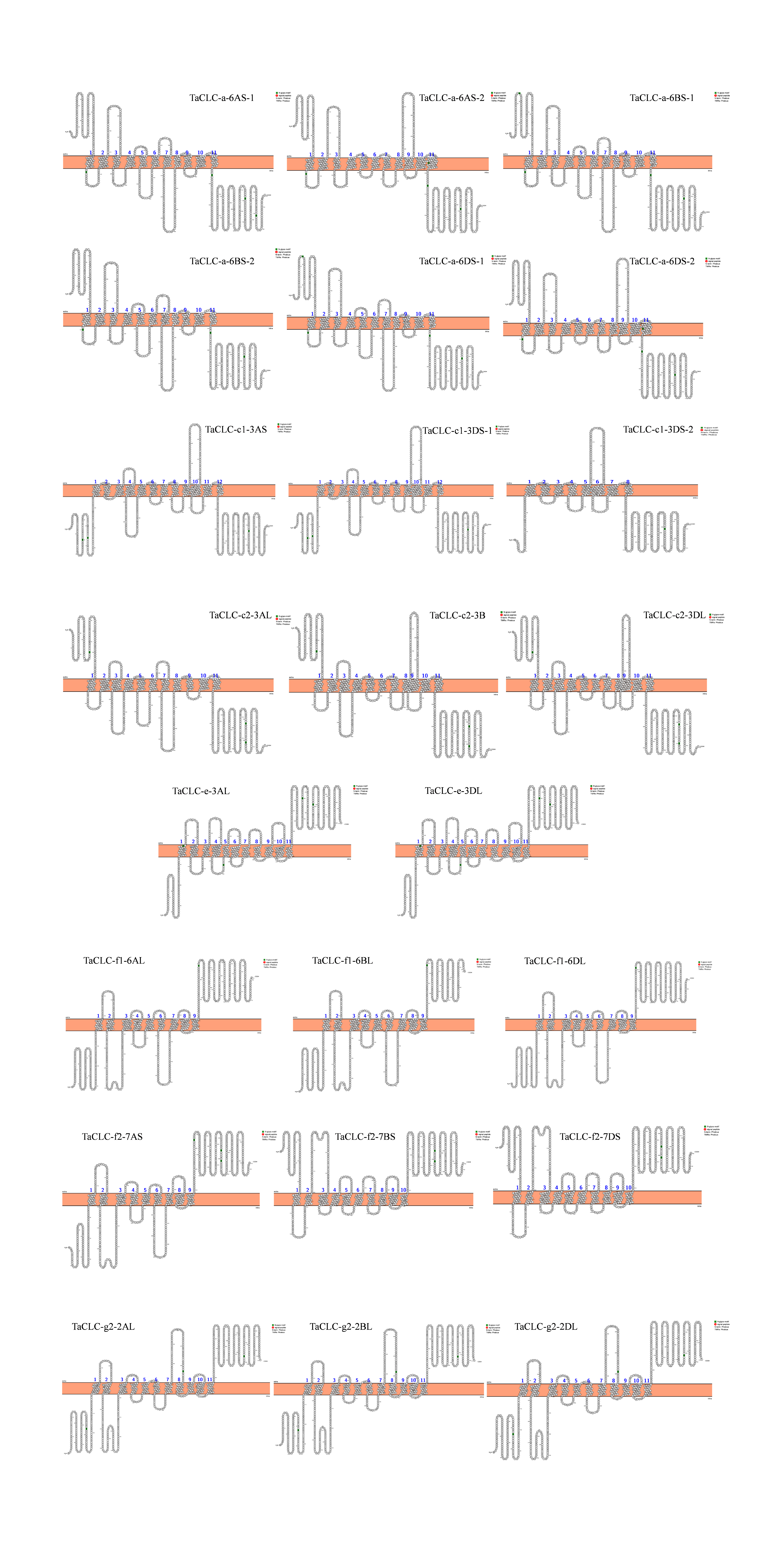

Supplement: Supplementary file 5 [file Image1.tif]
