# Supplementary material for: Genome-Wide Identification and Functional Characterization of the Chloride Channel TaCLC Gene Family in Wheat (Triticum aestivum L.)
Source: Front Genet. 2022 Mar 16;13:846795. doi: 10.3389/fgene.2022.846795 (PMC8966409; doi:10.3389/fgene.2022.846795)
Supplement: Supplementary file 6 [file Table2.DOC]

Supplementary Table. 1 The protein loci of rice, soybean, and Arabidopsis TaCLC family proteins.AtCLCs gene protein sequence can be found in NCBI and Ensembl Plant according to original gene name, OsCLCs gene protein sequence can be found in RiceDate according to original gene name, and GmCLCs gene protein sequence can be found in SoyBase according to original gene name.

| gene name | original gene name | amino acid sequence |
| --- | --- | --- |
| *AtCLC-a* | *CAA96057/AT5G40890* | MDEDGNLQISNSNYNGEEEGEDPENNTLNQPLLKRHRTLSSTPLALVGAKVSHIESLDYEINENDLFKHDWRSRSKAQVFQYIFLKWTLACLVGLFTGLIATLINLAVENIAGYKLLAVGYYIAQDRFWTGLMVFTGANLGLTLVATVLVVYFAPTAAGPGIPEIKAYLNGIDTPNMFGFTTMMVKIVGSIGAVAAGLDLGKEGPLVHIGSCIASLLGQGGPDNHRIKWRWLRYFNNDRDRRDLITCGSASGVCAAFRSPVGGVLFALEEVATWWRSALLWRTFFSTAVVVVVLRAFIEICNSGKCGLFGSGGLIMFDVSHVEVRYHAAHIIPVTLIGVFGGILGSLYNHLLHKVLRLYNLINQKGKIHKVLLSLGVSLFTSVCLFGLPFLAECKPCDPSIDEICPTNGRSGNFKQFNCPNGYYNDLSTLLLTTNDDAVRNIFSSNTPNEFGMVSLWIFFGLYCILGLITFGIATPSGLFLPIILMGSAYGRMLGTAMGSYTNIDQGLYAVLGAASLMAGSMRMTVSLCVIFLELTNNLLLLPITMFVLLIAKTVGDSFNLSIYEIILHLKGLPFLEANPEPWMRNLTVGELNDAKPPVVTLNGVEKVANIVDVLRNTTHNAFPVLDGADQNTGTELHGLILRAHLVKVLKKRWFLNEKRRTEEWEVREKFTPVELAEREDNFDDVAITSSEMQLYVDLHPLTNTTPYTVVQSMSVAKALVLFRSVGLRHLLVVPKIQASGMSPVIGILTRQDLRAYNILQAFPHLDKHKSGKAR |
| *AtCLC-b* | *CAA96058/AT3G27170* | MVEEDLNQIGGNSNYNGEGGDPESNTLNQPLVKANRTLSSTPLALVGAKVSHIESLDYEINENDLFKHDWRKRSKAQVLQYVFLKWTLACLVGLFTGLIATLINLAVENIAGYKLLAVGHFLTQERYVTGLMVLVGANLGLTLVASVLCVCFAPTAAGPGIPEIKAYLNGVDTPNMFGATTMIVKIVGSIGAVAAGLDLGKEGPLVHIGSCIASLLGQGGTDNHRIKWRWLRYFNNDRDRRDLITCGSAAGVCAAFRSPVGGVLFALEEVATWWRSALLWRTFFSTAVVVVVLREFIEICNSGKCGLFGKGGLIMFDVSHVTYTYHVTDIIPVMLIGVIGGILGSLYNHLLHKVLRLYNLINEKGKIHKVLLSLTVSLFTSVCLYGLPFLAKCKPCDPSIDEICPTNGRSGNFKQFHCPKGYYNDLATLLLTTNDDAVRNLFSSNTPNEFGMGSLWIFFVLYCILGLFTFGIATPSGLFLPIILMGAAYGRMLGAAMGSYTSIDQGLYAVLGAAALMAGSMRMTVSLCVIFLELTNNLLLLPITMIVLLIAKTVGDSFNPSIYDIILHLKGLPFLEANPEPWMRNLTVGELGDAKPPVVTLQGVEKVSNIVDVLKNTTHNAFPVLDEAEVPQVGLATGATELHGLILRAHLVKVLKKRWFLTEKRRTEEWEVREKFPWDELAEREDNFDDVAITSAEMEMYVDLHPLTNTTPYTVMENMSVAKALVLFRQVGLRHLLIVPKIQASGMCPVVGILTRQDLRAYNILQAFPLLEKSKGGKTH |
| *AtCLC-c* | *CAA96059/AT5G49890* | MDDRHEGDHHDIEVEGGALHGFERKISGILDDGSVGFRQPLLARNRKNTTSQIAIVGANTCPIESLDYEIFENDFFKQDWRSRKKIEILQYTFLKWALAFLIGLATGLVGFLNNLGVENIAGFKLLLIGNLMLKEKYFQAFFAFAGCNLILATAAASLCAFIAPAAAGSGIPEVKAYLNGIDAYSILAPSTLFVKIFGSIFGVAAGFVVGKEGPMVHTGACIANLLGQGGSKKYRLTWKWLRFFKNDRDRRDLITCGAAAGVAAAFRAPVGGVLFALEEAASWWRNALLWRTFFTTAVVAVVLRSLIEFCRSGRCGLFGKGGLIMFDVNSGPVLYSTPDLLAIVFLGVIGGVLGSLYNYLVDKVLRTYSIINEKGPRFKIMLVMAVSILSSCCAFGLPWLSQCTPCPIGIEEGKCPSVGRSSIYKSFQCPPNHYNDLSSLLLNTNDDAIRNLFTSRSENEFHISTLAIFFVAVYCLGIITYGIAIPSGLFIPVILAGASYGRLVGRLLGPVSQLDVGLFSLLGAASFLGGTMRMTVSLCVILLELTNNLLMLPLVMLVLLISKTVADCFNRGVYDQIVTMKGLPYMEDHAEPYMRNLVAKDVVSGALISFSRVEKVGVIWQALKMTRHNGFPVIDEPPFTEASELCGIALRSHLLVLLQGKKFSKQRTTFGSQILRSCKARDFGKAGLGKGLKIEDLDLSEEEMEMYVDLHPITNTSPYTVLETLSLAKAAILFRQLGLRHLCVVPKTPGRPPIVGILTRHDFMPEHVLGLYPHIDPLK |
| *AtCLC-d* | *CAA96065/AT5G26240* | MLSNHLQNGIESDNLLWSRVPESDDTSTDDITLLNSHRDGDGGVNSLDYEVIENYAYREEQAHRGKLYVGYYVAVKWFFSLLIGIGTGLAAVFINLSVENFAGWKFALTFAIIQKSYFAGFIVYLLINLVLVFSSAYIITQFAPAAAGSGIPEIKGYLNGIDIPGTLLFRTLIGKIFGSIGSVGGGLALGKEGPLVHTGACIASLLGQGGSTKYHLNSRWPQLFKSDRDRRDLVTCGCAAGVAAAFRAPVGGVLFALEEVTSWWRSQLMWRVFFTSAIVAVVVRTAMGWCKSGICGHFGGGGFIIWDVSDGQDDYYFKELLPMAVIGVIGGLLGALFNQLTLYMTSWRRNSLHKKGNRVKIIEACIISCITSAISFGLPLLRKCSPCPESVPDSGIECPRPPGMYGNYVNFFCKTDNEYNDLATIFFNTQDDAIRNLFSAKTMREFSAQSLLTFLAMFYTLAVVTFGTAVPAGQFVPGIMIGSTYGRLVGMFVVRFYKKLNIEEGTYALLGAASFLGGSMRMTVSLCVIMVEITNNLKLLPLIMLVLLISKAVGDAFNEGLYEVQARLKGIPLLESRPKYHMRQMIAKEACQSQKVISLPRVIRVADVASILGSNKHNGFPVIDHTRSGETLVIGLVLRSHLLVLLQSKVDFQHSPLPCDPSARNIRHSFSEFAKPVSSKGLCIEDIHLTSDDLEMYIDLAPFLNPSPYVVPEDMSLTKVYNLFRQLGLRHLFVVPRPSRVIGLITRKDLLIEENGESSAVELQQSTSVRGRYSDTATRMDAARPLLDDLLG |
| *AtCLC-e* | *AEE86511/AT4G35440* | MAATLPLCAALRSPVSSRRFAPIHKTDVPFQFNVVLSPFFGSVAIGGRIFPRLPAAKQETDQDEVGFDQQPSQELAIASACLVGVLTGVSVVLFNNCVHLLRDFSWDGIPDRGASWLREAPIGSNWLRVILVPTIGGLVVSILNQLRESAGKSTGDSHSSLDRVKAVLRPFLKTVAACVTLGTGNSLGPEGPSVEIGASIAKGVNSLFNKSPQTGFSLLAAGSAAGISSGFNAAVAGCFFAVESVLWPSSSTDSSTSLPNTTSMVILSAVTASVVSEIGLGSEPAFKVPDYDFRSPGELPLYLLLGALCGLVSLALSRCTSSMTSAVDSLNKDAGIPKAVFPVMGGLSVGIIALVYPEVLYWGFQNVDILLEKRPFVKGLSADLLLQLVAVKIAATAWCRASGLVGGYYAPSLFIGGAAGMAYGKFIGLALAQNPDFNLSILEVASPQAYGLVGMAATLAGVCQVPLTAVLLLFELTQDYRIVLPLLGAVGMSSWITSGQSKRQETRETKETRKRKSQEAVQSLTSSDDESSTNNLCEVESSLCLDDSLNQSEELPKSIFVSEAMRTRFATVMMSTSLEEALTRMLIEKQSCALIVDPDNIFLGILTLSDIQEFSKARKEGNNRPKDIFVNDICSRSGGKCKVPWTVTPDMDLLAAQTIMNKHELSHVAVVSGSIDAPRIHPVGVLDRECITLTRRFPTLDVVEVKLVKKNYLQLFVCSSEERSESQSPGSSNQNVPPKFAVSVNGLAL |
| *AtCLC-f* | *AEE33275/AT1G55620* | MSSGGAGEYNEDRHLLRSTDGDEVGIGGGEGDLDVESQSPAIRSGAGGVRDLFKHIDRRFSLSGRRLSFKRMENIRVDRERHNPSSSSAFSAAGEEDGGGISNLHSVDDRNDEYGFDEEVLGDSAPPEWALLLIGCLIGVAAGICVAGFNKGVHVIHEWAWAGTPNEGAAWLRLQRLADTWHRILLIPVTGGVIVGMMHGLLEILDQIRQSNSSQRQGLDFLAGIYPVIKAIQAAVTLGTGCSLGPEGPSVDIGKSCANGFALMMENNRERRIALTAAGAASGIASGFNAAVAGCFFAIETVLRPLRAENSPPFTTAMIILASVISSTVSNALLGTQSAFTVPSYDLKSAAELPLYLILGMLCGAVSVVFSRLVTWFTKSFDFIKDKFGLPAIVCPALGGLGAGIIALKYPGILYWGFTNVEEILHTGKSASAPGIWLLAQLAAAKVVATALCKGSGLVGGLYAPSLMIGAAVGAVFGGSAAEIINRAIPGNAAVAQPQAYALVGMAATLASMCSVPLTSVLLLFELTKDYRILLPLMGAVGLAIWVPSVANQGKESDSSEGRSTGRGYSSLSPSERKTEGVWRHTDNADSLELTVIENPDHNSFLDEETILEDLKVMRVMSKNYVKVSSGTTLREARNILKESHQNCIMVVDDDDFLAGILTHGDIRRYLSNNASTILDENTCPVSSVCTKKISYRGQERGLLTCYPDATVGVAKELMEARGVKQLPVVKRGEVIHKGKRRKLLGLLHYDSIWTFLRDEMSRRRSINDRRKDKEVGTNGH |
| *AtCLC-g* | *AED93891/AT5G33280* | MPNSTTEDSVAVPLLPSLRRATNSTSQVAIVGANVCPIESLDYEIAENDFFKQDWRGRSKVEIFQYVFMKWLLCFCIGIIVSLIGFANNLAVENLAGVKFVVTSNMMIAGRFAMGFVVFSVTNLILTLFASVITAFVAPAAAGSGIPEVKAYLNGVDAPEIFSLRTLIIKIIGNISAVSASLLIGKAGPMVHTGACVASILGQGGSKRYRLTWRWLRFFKNDRDRRDLVTCGAAAGIAASFRAPVGGVLFALEEMSSWWRSALLWRIFFSTAVVAIVLRALIDVCLSGKCGLFGKGGLIMFDVYSENASYHLGDVLPVLLLGVVGGILGSLYNFLLDKVLRAYNYIYEKGVTWKILLACAISIFTSCLLFGLPFLASCQPCPVDALEECPTIGRSGNFKKYQCPPGHYNDLASLIFNTNDDAIKNLFSKNTDFEFHYFSVLVFFVTCFFLSIFSYGIVAPAGLFVPVIVTGASYGRFVGMLLGSNSNLNHGLFAVLGAASFLGGTMRMTVSTCVILLELTNNLLLLPMMMVVLLISKTVADGFNANIYNLIMKLKGFPYLYSHAEPYMRQLLVGDVVTGPLQVFNGIEKVETIVHVLKTTNHNGFPVVDGPPLAAAPVLHGLILRAHILTLLKKRVFMPSPVACDSNTLSQFKAEEFAKKGSGRSDKIEDVELSEEELNMYLDLHPFSNASPYTVVETMSLAKALILFREVGIRHLLVIPKTSNRPPVVGILTRHDFMPEHILGLHPSVSRSKWKRLRIRLPFFS |
| *GmCLC-1* | *Glyma05g14760* | MGEESSLLKESTSINDTNMVEEVEERDPESNPLNEPLLKRNRTLSSNPLALVGAKVSYIESLDYEINENDLFKQDWRSRSRTQVLQYIFWKWTLAFLVGLLTGVIATLINLAVENIAGYKFLAVVNFIQKERYLRGFLYFTGINFLLTFVASILCVCFAPTAAGPGIPEIKAYLNGVDTPNMYGATTLFVKIIGSIGAVSAGLDLGKEGPLVHIGSCIASLLGQGGPDNYRIKWRWLRYFNNDRDRRDLITCGASSGVFLFALEEVATWWRSALLWRTFFSTAVVVVVLRASIELCHKGKCGLFGEGGLIMYDVSDVTVRYNVMDIIPVVIIGVLGGVLGSLYNYLLHKVLRVYNLINQKGKMYKLLLSLSVAIFTSACQYGLPFLAKCTPCDPSLSDVCPTNGRSGNFKQFNCPKGYYNDLATLLLTTNDDAVRNIFSTNTPLEYQPSSIIIFFALYCILGLITFGIAVPSGLFLPIILMGSGYGRLLGILMGPHTNIDQGLFAVLGAASLMAGSMRMTVSLCVIFLELTNNLLLLPITMIVLLIAKTVGDSFNPSIYEIILHLKGLPFMDANPEPWMRNLTVGELVDVKPAVVSFKGVEKVANIVNALKNTTHNGFPVMDCGLVPTTGVANEATELHGIILRAHLIQVLKKKWFLKERRRTEEWEVREKFTWVELAEREGNIEDVAVTKEEMEMFVDLHPLTNTTPFTVLESMSVAKAMVLFRQVGLRHMLVVPKYQASGVSPVIGILTRQDLLAYNILTVFPHLAKSKRK |
| *GmCLC-b1* | *Glyma16g06190* | MGEDSREFGKSTKINHKMEEVQREEEIDPESNPLNEPLLLKRTRTLSSNPLALVGEKVSYIESLDYEINENDLFKHDWRSRSRVQVLQYIFLKWLLAFLVGLLTGIIATLINLAVENIAGYKLLAVLKYIHKERYLTGFLYFTGINFVLTFVAAILCVCFAPTAAGPGIPEIKAYLNGVDTPNMFGATTLIVKIIGSIGAVSAGLDLGKEGPLVHIGSCIASLLGQGGPDNYRTKWHWLRYFNNDRDRRDLITCGSSSGVCAAFRAPVGGVLFALEEVATWWRSALLWRTFFSTAVVVVVLRAFIEICHTGKCGLFGEGGLIMFDVSNVTVRYHVMDIVLVVVIGIIGGVLGSLYNHVLHKVLRLYNLINQKGRTHKLLLSLAVALFTSMCQYGLPFLAKCTPCDPSLPESACPTNGRSGNFKQFNCPPGYYNDLATLLLTTNDDAVRNIFSTNTPQEYQPLSLVIFFLLYCILGLITFGIAVPSGLFLPIILMGSGYGRLLGIYMGPHTNIDQGLFAVLGAASLMAGSMRMTVSLCVIFLELTNNLLLLPITMIVLLIAKTVGDSFNPSIYEIILHLKGLPFIDANPEPWMRNLTVGELVDVKPPVVTLHGVEKVAKIVDVLKNTTHNAFPVMDNGVVPPVVGQANGGTELHGLILRAHLIQAIKKKWFLKERRRTEEWEVREKFTWVELAEREGSIEEVAVTSEEMEMFVDLHPLTNTLPLQC |
| *GmCLC-b2* | *Glyma19g25680* | MGEDSGEFGESTKINHKMENVEREEEIDPESNPLNEPLLKRNRTLSSNPLALVGEKVSYIESLDYEINENDLFKHDWRSRSRVQVLQYIFLKWLLAFLVGLLTGIIATLINLAVENIAGYKLLAVLKYIHKERYLTGFLYFTGINFVLTFVAAILCVCFAPTAAGPGIPEIKAYLNGVDTPNMFGATTLIVKIIGSIGAVSAGLDLGKEGPLVHIGSCIASLLGQGGPDNYRIKWRWLRYFNNDRDRRDLITCGSSSGVCAAFRAPVGGVLFALEEVATWWRSALLWRTFFSTAVVVVVLRAFIEICHTGKCGLFGEGGLIMFDVSNVTVRYHVMDIVLVVVIGIIGGVLGSLYNHVLHKVLRLYNLINQKGRIHKLLLSLAVALFTSMCEYGLPFLAKCTPCDPSLPESTCPTNGRSGNFKQFNCPPGYYNDLATLLLTTNDDAVRNIFSTNTPQEYQPLSLVIFFVLYCILGLITFGIAVPSGLFLPIILMGSGYGRLLGIYMGPHTNIDQGLFAVLGAASLMAGSMRMTVSLCVIFLELTNNLLLLPITMIVLLIAKTVGDSFNPSIYEIILHLKGLPFMDANPEPWMRNLTVGELVDVKPSVVTLHGVEKVAKIVDVLKNTTHNAFPVMDDGVVPPVVGQANGGTELHGLILRAHLIQALKKKWFLKERRRTEEWEVREKFTWVELAEREGSIEEVAVTSEEMEMFVDLHPLTNTTPFTVLESMSVAKAMILFRQVGLRHLLVVPKYQASGVSPVIGILTRQDLLAHNILTVFPHLAISKGREKRN |
| *GmCLC-c1* | *Glyma09g28620* | MMTPTGGEGAEKMDAEDGVNCSDYEHDIENEESGGYWSGIYDRNMAHTMPLLMKRANTTSQIAIVGANPCPIESLDYEIFDNEILKNDWRSKKKVQIIHYVMLKWGFALLIGLGTGLVGFFNSFAVENIAGFKLLMTTGLMSKHRYLDAFLAYAGANMCLAAAAAALCAFIAPAAAGSGIPEVKAYLNGVDAQNILAPSTLFVKIFGSILGVSAGFVVGKEGPMVHTGACIASLLGQGGSHKYHLTCTWLRYFKNDRDRRDMITCGAAAGVAAAFRAPVGGVLFALEEAASWWRSALLWRTFFTTAVVAIVLRVAIQFCATGKCGLFGEGGLIMYDVSSANITYSASGIFAVLLLGAIAGILGSIYNYLVDKVVRTYSIINGKGAFSKISLVVTIALLTSCCYYFLPWIAKCIRCPSNSTVICPSVDESGDYKSFQCPPGYYNDLASLFLNTNDDAIRNLFSPRIIKEFHITSLFIYFATIYCLGIITYGIAIPSGLFIPVILAGAAYGRLFGRLFETITELDRGLFALLGAASFLGGTMRMTVSLCVILLELTNDLLLLPLVMLVLLVSKSVADSFNKGVYDQILKIKGLPYLEAHAEPYMRNLVTRDVVSGPLITFSGIEKVANILQALNTTGHNGFPVIDEPPFSDSPELCGLVLRSHLLVLLKEKIFSRDRGFANQRIFQRISTLDFGKAGSGKGIKLEDLDIQEEEMDMYVDLHPITNASPYTVVETMSLAKAAILFRQHGLRHMCVVPKSQGRPPVVGILTRHDFMPEHVLGLHPDIMPHKWK |
| *GmCLC-c2* | *Glyma16g33351* | MTPTSGEGAEKMDAEDGVNCSDYEHDIENEDLWDGNEESGGYWSGIYDRNKAHTKPLLMKGTNTTSQIAIIGANLCPIESLDYEIFDNEILKNDWSSRKKVQIIHYVMLKWGFALLIGLGTGLVGFFNSFAVENIAGFKLFMTTSLMSKHRYLEAFLAYAGANMCLAAAAAALCAFIAPAAAGSGIPEVKAYLNGVDAQHILAPSTLFVKIFGSILGVSAGFVVGKEGPMVHTGACIASLLGQGGSRKYHLTCTWLRYFKNDRDRRDMITCGAAAGVAAAFRAPVGGVLFALEEAATWWRSALLWRTFFTTAVVAIVLRVAIQFCATGGKCGLFGEGGLIMYDVSSANITYSASGIFAVLLMGAIAGILGSIYNYLVDKVVRTYSIINGKGAFSKISLVVTIALLTSCCYYFLPWIAYCIPCPSNSTVICPSVDESGEYKNFQCPPGYYNDLASLFLNTNDDAIRNLFSPRIIKEFHITSLFIYFATIYCLGIITYGIAIPSGLFIPVILAGAAYGRLFGRLFETITKLDRGLFALLGAASFLGGTMRMTVSICVILLELTNDLLLLPLVMLVLLVSKTVADSFNKGVYDQILKIKGLPYLEAHAEPYMRNLVTRDVVSGPLITFSGIEKVRNILHALHTTGHNGFPVIDEPPFSDAPELCGLVLRSHLLVLLKEKIFSRDRGFANPVIFQRISTLDFGKAGSGKEIKLEDLDIQEEEIDMYVDLHPITNASPYTVVETMSLAKAAILFRQHGLRHMCVVPKSQGRPPVVGILTRHDFMPEHVLGLYPDIKPHKWQ |
| *GmCLC-d1* | *Glyma01g44950* | MLSNHFQNGIETARLVWSRIPNSEESQLLDDAVGILKKNDGGGVESLDYEVIENFAYREEQAQRGKLYVSYLLVVKWFFALLIGICTGLAAVFINIAVENFAGWKFSVTFNIIQKSYIAGFVVYVLINLALVFSSVYIITQFAPAAAGSGIPEIKGYLNGVDIHGILLFRTLIGKIFGSIGSVGGGLALGKEGPLVHTGACIASLLGQGGSTKYHLNSRWFQVFKSDRDRRDLVTCGCAAGVAAAFRAPVGGVLFALEEVTSWWRSQLMWRVFFTSAVVAVVVRAAMGWCKSGKCGHFGSGGFIIWDISDGQEDYSFAELFPMAIIGVIGGLLGSLFNQLTLYITTWRRNHLHKKGNRVKIIEACLVSILTSAISFGLPLLRKCSPCPDSDPASGIECPRPPGMYGNYVNFFCSKDKEYNDLATIFFNTQDDAIRNLFSAKTINEYSSQSLLTFLVMFYALAVVTFGTAVPAGQFVPGIMIGSTYGRLVGMFVVKYYRKLNIEEGTYALLGAASFLGGSMRMTVSLCVIMVEISNNLKFLPLIMLVLLISKAVGDAFNEGIYEEQAQLRGIPLLESRPKYEMRNMTAKEACGSGRVVSFPRVVKVSDVVSILRSNKHNGFPVIDHTRSGEPLVIGLVLRSHLLVILQSKVDFQHSPLPSDPRGGGRSIRHDSGEFAKPVSSKGICIDDIHLSSDDLEMYIDLAPFLNPSPYIVPEDMSLTKVYNLFRQLGLRHLFVVPRPSRVLGLITRKDLLIEDKENVNTLELQSTSVRIPHQNKRLMTRNIDVEHPLLSGLLQNQIPD |
| *GmCLC-d2* | *Glyma11g00690* | MLANHFQNGIETARLVWSRIPNSEESQLLDDAVGILKKNDGGGVESLDYEVIENFAYREEQAQRGKLYVSYLLVVKWFFALLIGICTGLAAVVINIAVENFAGWKFSVTFNIIQKSYIAGFVVYVLINLALVFSSVYIITQFAPAAAGSGIPEIKGYLNGVDIHGILLFRTLIGKIFGSIGSVGGGLALGKEGPLVHTGACIASLLGQVGGVLSSVLFTLLCYKYPFFDKGGSTKYHLNSRWFQVFKSDRDRRDLVTCGCAAGVAAAFRAPVGGVLFALEEVTSWWRSQLMWRVFFTSAVVAVVVRAAMGWCKSGKCGHFGSGGFIIWDISDGQEDYSFAELFPMAIIGVIGGLLGSLFNQLTLYITTWRRNHLHKKGSRVKIIEACLVSILTSAISFGLPLLRKCSPCPDSDLASGIECPRPPGMYGNYVNFFCSKDKEYNDLATIFFNTQDDAIRNLFSAKTINEYSSQSLLTFLVMFYALAVITFGTAVPAGQFVPGIMIGSTYGRLVGMFVVKYYRRLNIEEGTYALLGAASFLGGSMRMTVSLCVIMVEISNNLKFLPLIMLVLLISKAVGDAFNEGIYEEQAQLRGIPLLESRPKYEMRNMTAKEACGSGRVVSFPRVVKVSDVVSILRSNKHNGFPVIDHTRSGEPLVIGLVLRSHLLVILQSKVDFQHSPLPSDPRGGGRSIRHDSGEFAKPVSSKGICIDDIHLSSDDLEMYIDLAPFLNPSPYIVPEDMSLTKVYNLFRQLGLRHLFVVPRPSCVVGLITRKDLLIEDKENVNTLELQSTSVRIPQQNKRLMTRNIDVERPLLNGLLQNQIPD |
| *GmCLC-g* | *Glyma13g23080* | MSTNYSTNGDSETLLRRPLLSSQRSIVNSTSQVAIVGSNVSPIESLDYEIFENEFFKHDWRSRGKAQIFQFMIMKWLLCLLIGMIVCLVGFCNNLAVENLAGIKFVVTSNMMLERRFLMAFLVFFVSNLVLTVFACTITALIAPTATGSGIPEVKAYLNGVDAPGIFTVRTLLVKIIGSITAVSSSLLIGKAGPMVHTGACVAALLGQGGSKRYGLTWKWLKFFKNDRDRRDLIICGSAAGIAAAFRAPVGGVLFALEGMSSWWRSALLWRAFFTAAIVAILLRALIDLCLSGKCGLFGKGGLIMFDAYSASISYHLVDVPPVFVLGVIGGILGSLFNLILSKVLRIYNFINEKGTIFKILLACLISIFTSCLLFGLPWLTSCRPCPPDPSEPCPTIGRSGIYKKFQCPPNHYNDLASLIFNTNDDAIRNLFSKNTDDEFEFKSVFIFFITCFSLSIFSYGVVAPAGLFVPVIVTGASYGRIVGMLLGKKDSLSHGLYAVLGAASFLGGSMRTTVSLCVIILELTNNLLLLPLIMMVLFISKTVADAFNANIYDIIMKAKGLPYLETHAEPYMRQLSVGDVVTGPLQTFNGVEKVCNIVFILRTTGHNGFPVIDEPPISQAPVLFGIILRDHLLTLLKKKAFMSSPMATSGDVINEFSADDFAKKGSSKGRLKIEDIQLSEEEMDMFIDLHPFTNASPYTVVETMSLGKALTLFRELGLRHLLVVPKFSGRSPVVGILTRHDFMSEHILGLHPFLVRNTGKSLRF |
| *OsCLC-a* | *Os12g25200* | MLCVVFAPTAAGPGIPEIKAYLNGVDTPNMFGAPQLIVKIIGSICAVSSGLDLGKEGPLVHIGACLANLLSQGGSGRHRLRLRWLRYFDNDRDRRDLITCGASSGVCAAFRAPVGGVLFALEEVATWWRSALLWRTFFSTATVVVVLRGFIEVCRNGRCGLFGEGGLILFDVGDVAVRYHAGDLLPVTIVGVLGGVLGALYNHVLHKVLRVYNLINEKGRAAKLALALAVCALTSALLYVTPFAVPCTPCDPAFGGACPTLGKSGNFKRFNCPEGHYNDLATLLHATNVDATRNIFSTGTAGEFRLDSLLIFFAVYCVLGLFTFGIAVPSGLFLPIILMGSAYGRVTALVLSRFARIDHGLYAVLGAAALMSGSMRMTVSLVVIFLELTNNLLLLPITMFVLLIAKTVGDAFNPSIYEIILDLKGLPFLEAKPEPWMKDLTVGELAAAKPRAVALQVVERVSTVVEALRATRHNGFPVLDRPRPGVSELHGLVLRSHLVAALRKRWFLPERRRTEEWEAREMFSSAELADKCGGVDELEISPEEMGMYVDLHPLTNTTPYTVVETMSVAKAVVLFRSVALRHMLIMPKFQGPEISPIVGILTRQDLIAHNILGAFPHLASKRKTH |
| *OsCLC-c1* | *Os02g35190* | MEGQSQHRAPEREGSHNYDIESTDGSGGLWRRNGSSSGALLRYNDSGGGRSGSAGEPLLRKRTMNTTSQIAIVGANVCPIESLDYEVVENDLFKQDWRSRKKKQIFQYIVLKWTLVLLIGLLTGLVGFFNNLAVENIAGFKLLLTGNLMLKERYLTAFFAYGGCNLVLAAAAAAICAYIAPAAAGSGIPEVKAYLNGVDAYSILAPSTLFVKIFGSILGVSAGFVLGKEGPMVHTGACIANLLGQGGSRKYRLTCNWLRYFKNDRDRRDLITCGSAAGVAAAFRAPVGGVLFALEEAASWWRSALLWRAFFTTAVVAVVLRSLIEFCRSGKCGLFGQGGLIMFDLSSTVATYSTPDLIAIIILGIIGGIFGGLFNFLLDKVLRVYSIINERGAPFKILLTITISIITSMCSYGLPWLAACTPCPVDAVEQCPTIGRSGNFKNFQCPPGHYNDLASLFFNTNDDAIRNLFSNGTESEFHMSTLFIFFTAVYCLGILTYGVAVPSGLFIPVILAGATYGRIVGTLLGSISDLDPGLFALLGAASFLGGTMRMTVSVCVILLELTNDLAMLPLVMLVLLISKTIADNFNKGVYDQIVVMKGLPYMEAHAEPYMRHLVAGDVVSGPLITFSGVEKVGNIVHALRFTGHNGFPVVDEPPLTEAPELVGLVTRSHLLVLLNGKMFMKDQLKTSGSFVLQRFGAFDFAKPGSGKGLKIQDLDFTDEEMEMYVDLHPVTNTSPYTVVETMSLAKAAILFRALGLRHLLVVPKTPDRPPIVGILTRHDFVEEHIHGLFPNLNPHKFHSTSMGG |
| *OsCLC-c2* | *Os01g65500* | MAVGTESGSGGVAESAPSTPMSAMTTTPISARSVRWESDMPDIEEGGGGLERPLLRRRGTNTTSQMAIVGANVCPIESLDYELVENEVYKQDWRSRGKLQIFHYQILKWVLALLVGLIVGLIGFFNNIAVENIAGFKLLLTTNLMLQNRYKAAFLWFISCNAMLAAAAAALCAYFGPAAAGSGIPEVKAYLNGVDAPSILAPSTLFVKIVGSIFGVSAGFVLGKEGPMVHTGACVASFLGQGGSRKYGFTWNWLRYFKNDLDRRDLITCGAAAGVTAAFRAPVGGVLFALEEATSWWRSALLWRTFSTTAVAAMVLRSLIEYCRSGNCGLFGKGGLIMFDVSSQVTSYTTMDLAAVVLLAIVGGLLGALFNFLLNRILRVYSYINEKGAPYKIILTVVISLVTSCCSFGLPWLTACTPCPPELAASGHCPTIGRSGNFKNFRCPPGQYNAMASLFLNTNDDAIRNLFSGGTESEFGVPMLLAFFTAVYSLGLVTYGVAVPSGLFIPVILSGASFGRLLGKLLGVLTGLDTGLFALLGAASFLGGTMRMTVSVCVILLELTNDLLLLPLIMLVLLVSKTVADCFNKGVYEQMVRMKGLPYLEAHAEPCMRSLVAGDVVSAPLIAFSSVESVGTVVDTLRRTGHNGFPVIEDAPFAPEPELCGLVLRSHLLVLLRAKTFTADRVKTGAAEVFRKLAPFDFAKPGSGKGLTVDDLDLTEEEMAMYVDLHPIANRSPYTVVENMSLAKAAVLFRQLGLRHMCVVPRTPGRPPVVGILTRHDFMPGYIRGLFPNVLRE |
| *OsCLC-d* | *Os03g48940* | MARLAWTRLPTADGAGAGAEGPGPVPASPSSAGYELFAGGVESLDYEVIENYAYREEQAQRSKFWVPYYVMLKWLFSLLIGVGTGLAAIFINLAVENFSGWKYAATFAIIQHSYFVGFFVYIVFNLALVFSSVYIVTNFAPAAAGSGIPEIKGYLNGVDTHGILLFRTLVGKIFGSIGSVGGGLALGKEGPLVHTGACIASLLGQGGSAKYHLSSRWVRIFESDRDRRDLVTCGCAAGVAAAFRAPVGGVLFALEEVTSWWRSHLMWRVFFTSAVVAVVVRSAMNWCKSGKCGHFGSGGFIIWDISGGQEDYSYQELLPMAIIGVIGGLLGALFNQLTLYITKWRRTYLHKKGKRVKIFEACLISLVTSTISFVLPLMRKCSSCPQLETNSGIECPRPPGTDGNFVNFYCSKDNEYNDLATIFFNTQDDAIRNLFSAKTFHEYSAQSLITFLVMFYSLAVVTFGTAVPAGQFVPGIMIGSTYGRLVGMFVVKFYKKLNVEEGTYALLGAASFLGGSMRMTVSLCVIMVEITNNLKLLPLIMLVLLISKAVGDFFNEGLYEVQAQLRGIPLLDSRPKQVMRNMSAKDACKNQKVVSLPRVSRIVDIISVLRSNKHNGFPVVDRGQNGESLVIGLILRSHLLVLLQSKVDFQNSPFPCGPGILNRHNTSDFVKPASSKGKSIDDIHLTEDELGLYLDLAPFLNPSPYIVPEDMSLAKV |
| *OsCLC-e* | *Os01g50860* | MSELPLYLLLGIFCGLVSTTLSRCTALSMEIVQSLQTATGLPKAASPALGGLIVGLLALVYPEVLYWGFENVDILLESRPFTSGLSAAVLVQLIGVKVLATSLCRAFGLVGGYYAPSLFIGAATGMAYGKVMRFTFTGPDSLFQIPFLDVASPQAYGLVGMAATLAGVCKVPLTSVLLLFELTQDYRIVLPLLGAVGVSSWIASPQRFSRSNRSKPDSSEVKSSNFKQEKSVPSQTQGASVDTDKPITDLCKLESSLCVYDAKHENFQENLTVAEAMKTKYISVSKTTPVVEALNLMLVEKQPFVMIIESNRSLIGLVTLKDIQDFCRTAKTTRVQTEEPVQTYVCGAVKCKMWPVTPQTSLTTVEKIMDSYGVDQLPVVSEHVDRQDRGLLVGFVDKEGIAIARRAVATKDLLSFTSEIKGDR |
| *OsCLC-f* | *Os08g38980* | MMAQASDLEPLRSGAGALPSSADPDSPSTPRRSRVRELLRSLDRRLSSRGRHHRHAAEGAAASPRGGGGGEPGSEDSDELGDGAPPEWALLLVGCLLGLATGICVAAFNRGVHIIHEWAWAGTPTEGAAWLRLQRLADTWHRILLIPVTGGVVVGMMHGLLEIFEQIKQSLSSQREGVDFMAAIFPTIKAIQAAITLGTGCSLGPEGPSVDIGKSCAYGCAEMMENNRERRIALVAAGSAAGIASGFNAAVAGCFFAIETVLRPLRAENSPPFTTAMIILASVISSTVSNVLLGEKAAFIVPTYELKSAAELPLYLILGMLCGVVSVAFRQLVVWFTKTFDLIRKKFSLPAVVCPALGGLGAGLIALRYPGILYWGFTNVDEILHTGKSASAPGIWLLAQLAAAKVVATALCKGSGLVGGLYAPSLMIGAAVGAVFGGSAAELINSAIPGNTAVAHPQAYALVGMAATLASVCSVPLTSVLLLFELTKDYRILLPLMGAVGLAIWVPSVVMSGNKEAFEATSPRHGYSSLLPPTDRNETDRRRPDRDDVELAILDDDPYRYGINNEDMLLDDLKVSQAMSKQFIKVTPTVTIKEATRLMHDKQQNCVLVVDSEDFLEGIVTIGDIRRKGFESELSEDTPRNGANSSTLDANSSLVSSCLTRGFQYHGNERGLVTCFPDTDLSTAKVLMEVKGIKQLPVVKRRAGRRNDGRRKVLGLLHYDSIGWCLREELERWKAIYQRENFQQSAVNGH |
| *OsCLC-g1* | *Os08g20570* | MACTAPREEDLLMEEDEHRPPLNRALLHRSATNNTSQVAMVGSNPCPIESLDYEIIENDLFDQNWRSRGKADQVRYVVLKWTFCFAIGIITGIAGFVINLAVENVAGLKHTAVSALMESSSYWTAFWLFAGTNLALLLFASSITAFVSPAAGGSGIPEVKAYLNGVDAPNIFSLRTLAVKIIGNIAAVSSSLHVGKAGPMVHTGACIAAIFGQGGSRKYGLTCRWLRYFKNDRDRRDLVTIGAGAGVTAAFRAPVGGVLFALESLSSWWRSALIWRSFFTTAVVAVVLRMFIELCASGKCGLFGKGGLIMYDVSTKFDDLMTYHLKDIPIVVLIGVIGAILGALYNFLMMKVLRVYSVINERGNAHKLLLAAVVSILTSCCVFGLPWLAPCRPCPTAGAPSPPNGTCHSLNRFRRFHCPAGHYNDLASLFLNINDDAIRNLYSTGTNDVYHPGSMLAFFVASYALGVLSYGVVAPSGLFVPIILTGATYGRLVAMLLGGRSGLDHGLVAILGSASFLGGTLRMTVSVCVIILELTNNLLLLPLVMLVLLISKTVADSFNSSIYDLILNLKGLPHLDGHAEPYMRQLTVGDVVAGPLRSFNGVEKVGHIVHTLRTTGHHAFPVVDEPPFSPAPVLYGLVLRAHLLVLLKKREFLTAPVRCPKDYMAGRFEAQDFDKRGSGKQDTIADVELSPEEMEMYVDLHPFTNTSPYTVVETMSLAKALVLFREVGLRHLLVVPKSCDRSPVVGILTRHDFMPEHILGLHPVLVGSRWKRLRWQKGAVAKKFRSLLDWLANDSG |
| *OsCLC-g2* | *Os04g55210* | MAPRDQSCGDGGEVDPEGGIEAPLLSSGSSFFQDPAHEDGDGDEEARRRRRRFLLAGRSQSNTTSQVALVGVGVCPIESLDYELIENEVFKQDWRARGRGHILRYVALKWALCFLVGVLSAAAGFVANLGVENVAGAKFVVTSNLMLAGRYGTAFAVFLVSNFALTMLATVLTVYVAPAAAGSGIPEVKAYLNGVDAPDIFSLKTLVVKIVGCIAAVSSSLHVGKAGPLVHTGACIASILGQGGSSKYHLTCKWLRYFKNDRDRRDLVTCGAGAGIAAAFRAPVGGVLFALEAVSSWWRSALLWRAFFTTAMVAVVLRALIDFCKSDKCGLFGKGGLIMFDVTSDYITYHLVDLPPVITLGVLGGVLGSLHNFFLDKVLRLYNFINEKGQKYKLLLAAVVTICTSCCLFGLPWIASCKPCPSDTEEACPSIGRSGNFKKYQCAMNEYNDLASLFFNTNDDTIRNLYSAGTDDEFHISSILVFFFTSYFLGIFSYGLALPSGLFVPVILTGATYGRLVGMLIGSQSTLDHGLFAVLGSAALLGGSMRMTVSVCVVILELTNNLLMLPLVMLVLLISKTVADAFNANIYDLLVKLKGFPYLEGHVEPYMRQLSVSDVVTGPLQAFNGIEKVGHIVHVLRTTGHNGFPVVDEPPFSDSPVLFGLVLRAHLLVLLRKKDFIPNCSASALDASKQFLPHDFAKPGSGKHDRIEEIEFSAEELEMFVDLHPFTNTSPYTVVETMSLAKAHVLFREVGLRHLLVLPKSSKRAPVVGILTRHDFMPEHILGLHPFLFKTRWKKVRFGKSAFTNLVF |

Supplementary Table. 2 Improved Hoagland nutrient solution formula

| Element name | Reagent | Concentration (mol/L) |
| --- | --- | --- |
| N | NH_4_NO_3_ | 2×10^-3^/0 |
| K | K_2_SO_4_ | 1×10^-3^ |
| P | KH_2_PO_4_ | 5×10^-4^ |
| Ca | CaCl_2_、CaSO_4_ | 2.5×10^-3^ |
| Mg | MgSO_4_ | 1×10^-3^ |
| Fe | Fe-Na-EDTA | 1×10^-4^ |
| Mn | MnSO_4_·7H2O | 1×10^-6^ |
| Cu | CuSO_4_·5H2O | 5×10^-7^ |
| Zn | ZnSO_4_·7H2O | 1×10^-6^ |
| B | H_3_BO_3_ | 1×10^-6^ |
| Mo | Na_2_MoO_4_ | 3.5×10^-8^ |

Supplementary Table. 3 qRT-qPCR primer list

| Primer name | primer sequence(5 '- 3') | Primer name | primer sequence(5 '- 3') |
| --- | --- | --- | --- |
| TaCLC-a-6AS-1-RT-F | GAGAAGCCCCTGCTGAAGA | TaCLC-a-6AS-1-RT-R | GATGAGCGACGCGATGAC |
| TaCLC-c1-3AS-RT-F | GTTCTGTCGCAGTGGAAAGTG | TaCLC-c1-3AS-RT-R | GGCATCCTCGGGGCATT |
| TaCLC-c2-3AL-RT-F | GGCTAAACGGGAGAAAACCT | TaCLC-c2-3AL-RT-R | TTTGAGGACCTGGTACTGGAAG |
| TaCLC-e-3AL-RT-F | TCTTCATCCCTTTCCAACTCAA | TaCLC-e-3AL-RT-R | AGCCATCGGCAATCCTGT |
| TaCLC-f1-6AL-RT-F | AAAGGATTACAGGATTTTGCTACC | TaCLC-f1-6AL-RT-R | GCACATTTGCTGCTTATCACTC |
| TaCLC-f2-7BS-RT-F | CAACTGCTATGATTATTTTGGCATC | TaCLC-f2-7BS-RT-R | CAGGGTATCTTAGAGCTATTAAACCTG |
| TaCLC-g2-2DL-RT-F | ACCAACGATGACACCATTAGAAA | TaCLC-g2-2DL-RT-R | GAAGGGCAGCAGAACCAAG |
| TaCLC-a-6AS-1-F | CCATCCTCCTTGACCTGACC | TaCLC-a-6AS-1-R | GACGCAAGCAAATCCTGAAC |
| TaCLC-c1-3AS-F | GCTGCTGTGCTACGTCTACGC | TaCLC-c1-3AS-R | CCCACTTCCTTGACCTTCTGG |
| TaCLC-e-3AL-F | GCTACCAGTCCCGCAATCG | TaCLC-e-3AL-R | CTTCAGCCAGCTACTTCACTTTC |
| TaCLC-a-6AS-1-p416-F | TTAGTTTCGACGGATTCTAGAATGGAGGAAGACCAGAGCCC | TaCLC-a-6AS-1-p416-R | GTCGACGGTATCGATAAGCTTTCAGTGCGTCTTGCTCTTCTTG |
| TaCLC-c1-3AS-p416-F | TTAGTTTCGACGGATTCTAGAATGGACGGAGGCCAATCG | TaCLC-c1-3AS-p416-R | GTCGACGGTATCGATAAGCTTTCAACCTCCCATTGAGGCTG |
| TaCLC-e-3AL-p416-F | TTAGTTTCGACGGATTCTAGAATGGCGCCAGCAGCG | TaCLC-e-3AL-p416-R | GTCGACGGTATCGATAAGCTTTCAACTCTCTTCCCTTCTGATCTC |
| p416-F | TCCCTGAAATTATTCCCCTACTTG | p416-R | TTCGGTTAGAGCGGATGTGG |

Supplementary Table. 5 Colinearity analysis result of TaCLCs gene between *Triticum aestivum* L. and *Arabidopsis thaliana*, *Oryza sativa* L., *Triticum dicoccoides*.

| Chromosomes name of TaCLC Gene Sequence in Wheat | TaCLC gene sequence of wheat | | Chromosomes name of Gene sequences with collinearity in other species | Gene sequences with collinearity in other species |
| --- | --- | --- | --- | --- |
| 7B | transcript:TraesCS7B02G136300.1 | 1 | | transcript:AT1G55620.2 |
| 7D | transcript:TraesCS7D02G239700.3 | 1 | | transcript:AT1G55620.2 |
| 2A | transcript:TraesCS2A02G517500.3 | 4 | | transcript:Os04t0644800-01 |
| 2B | transcript:TraesCS2B02G546000.1 | 4 | | transcript:Os04t0644800-01 |
| 2D | transcript:TraesCS2D02G519000.2 | 4 | | transcript:Os04t0644800-01 |
| 3A | transcript:TraesCS3A02G253600.3 | 1 | | transcript:Os01t0704700-01 |
| 3A | transcript:TraesCS3A02G390100.1 | 1 | | transcript:Os01t0876100-00 |
| 3B | transcript:TraesCS3B02G418700.1 | 1 | | transcript:Os01t0876100-00 |
| 3B | transcript:TraesCS3B02G285500.1 | 1 | | transcript:Os01t0704700-01 |
| 3D | transcript:TraesCS3D02G379600.1 | 1 | | transcript:Os01t0876100-00 |
| 6A | transcript:TraesCS6A02G283600.3 | 2 | | transcript:Os02t0720700-01 |
| 6D | transcript:TraesCS6D02G264100.1 | 2 | | transcript:Os02t0720700-01 |
| 7A | transcript:TraesCS7A02G240700.2 | 8 | | transcript:Os08t0499200-01 |
| 7B | transcript:TraesCS7B02G136300.1 | 8 | | transcript:Os08t0499200-01 |
| 7D | transcript:TraesCS7D02G239700.3 | 8 | | transcript:Os08t0499200-01 |
| 2A | transcript:TraesCS2A02G517500.3 | 2A | | transcript:TRIDC2AG072610.5 |
| 2A | transcript:TraesCS2A02G517500.3 | 2B | | transcript:TRIDC2BG078810.2 |
| 2B | transcript:TraesCS2B02G546000.1 | 2A | | transcript:TRIDC2AG072610.5 |
| 2B | transcript:TraesCS2B02G546000.1 | 2B | | transcript:TRIDC2BG078810.2 |
| 2D | transcript:TraesCS2D02G519000.2 | 2A | | transcript:TRIDC2AG072610.5 |
| 2D | transcript:TraesCS2D02G519000.2 | 2B | | transcript:TRIDC2BG078810.2 |
| 3A | transcript:TraesCS3A02G253600.3 | 3A | | transcript:TRIDC3AG038010.11 |
| 3A | transcript:TraesCS3A02G390100.1 | 3A | | transcript:TRIDC3AG055590.2 |
| 3A | transcript:TraesCS3A02G125300.1 | 3A | | transcript:TRIDC3AG015550.2 |
| 3A | transcript:TraesCS3A02G253600.3 | 3B | | transcript:TRIDC3BG042750.8 |
| 3A | transcript:TraesCS3A02G125300.1 | 3B | | transcript:TRIDC3BG020310.1 |
| 3A | transcript:TraesCS3A02G390100.1 | 3B | | transcript:TRIDC3BG061820.1 |
| 3B | transcript:TraesCS3B02G285500.1 | 3A | | transcript:TRIDC3AG038010.11 |
| 3B | transcript:TraesCS3B02G418700.1 | 3A | | transcript:TRIDC3AG055590.2 |
| 3B | transcript:TraesCS3B02G285500.1 | 3B | | transcript:TRIDC3BG042750.8 |
| 3B | transcript:TraesCS3B02G418700.1 | 3B | | transcript:TRIDC3BG061820.1 |
| 3D | transcript:TraesCS3D02G126600.1 | 3A | | transcript:TRIDC3AG015550.2 |
| 3D | transcript:TraesCS3D02G379600.1 | 3A | | transcript:TRIDC3AG055590.2 |
| 3D | transcript:TraesCS3D02G379600.1 | 3B | | transcript:TRIDC3BG061820.1 |
| 3D | transcript:TraesCS3D02G126600.1 | 3B | | transcript:TRIDC3BG020310.1 |
| 6A | transcript:TraesCS6A02G283600.3 | 6A | | transcript:TRIDC6AG043290.3 |
| 6A | transcript:TraesCS6A02G098500.2 | 6A | | transcript:TRIDC6AG012750.1 |
| 6A | transcript:TraesCS6A02G283600.3 | 6B | | transcript:TRIDC6BG050880.1 |
| 6A | transcript:TraesCS6A02G098500.2 | 6B | | transcript:TRIDC6BG017830.1 |
| 6B | transcript:TraesCS6B02G126400.1 | 6A | | transcript:TRIDC6AG012750.1 |
| 6B | transcript:TraesCS6B02G312100.1 | 6B | | transcript:TRIDC6BG050880.1 |
| 6B | transcript:TraesCS6B02G126400.1 | 6B | | transcript:TRIDC6BG017830.1 |
| 6D | transcript:TraesCS6D02G264100.1 | 6A | | transcript:TRIDC6AG043290.3 |
| 6D | transcript:TraesCS6D02G084000.2 | 6A | | transcript:TRIDC6AG012750.1 |
| 7A | transcript:TraesCS7A02G240700.2 | 7A | | transcript:TRIDC7AG030750.3 |
| 7A | transcript:TraesCS7A02G240700.2 | 7B | | transcript:TRIDC7BG021630.1 |
| 7B | transcript:TraesCS7B02G136300.1 | 7A | | transcript:TRIDC7AG030750.3 |
| 7B | transcript:TraesCS7B02G136300.1 | 7B | | transcript:TRIDC7BG021630.1 |
| 7D | transcript:TraesCS7D02G239700.3 | 7A | | transcript:TRIDC7AG030750.3 |
| 7D | transcript:TraesCS7D02G239700.3 | 7B | | transcript:TRIDC7BG021630.1 |

Supplementary Table. 5 the 33 motifs of TaCLC gene family

| Motif | Motif Consensus |
| --- | --- |
| 1 | KIDDLELTPEELEMYVDLHPFTNTSPYTVVETMSLAKAVVLFREVGLRHMLVVPKTP |
| 2 | KQSNTNTTSQLAMVGAKVCPIESLDYEIIENDLFKQDWRSRGRVEILQYIFLKWALAFLVGLLTGVIAFLINLAVENIAGLKMLHTSNLMREKRYWAAFLYFSGFNLVLTFVAAVLCVYFAPAAAGSGIPEVKAYLNGVDAPNIFGAPTLIVKIIGSIC |
| 3 | MRMTVSVCVILLELTNBLLLLPLVMLVLLISKTVADAFNPSVYEIIVDLKGLPFLEPHAEPYMRDLTVGDVVAG |
| 4 | LRWRWLRYFNNDRDRRDLITCGASSGVCAAFRSPVGGVLFALEEVATWWRSALLWRTFFSTATVVVVLRGFIEVCRGGRCGLFGZGGLI IFDVSDVTVRYRLGDLLLVTLVGVIGGVLGALYNHVLHMVLRLYNLINDKGRMAKLALALAVCVFTSAGLYVLPFAVPCTPCDPAFGAA CPATGRSGNFKQFNCPAGQYND |
| 5 | HLGKEGPLVHTGACIAAILGQGGSRKYRLTWKWJRYFKNDRDRRDLITCGAAAGVAAAFRAPVGGVLFALEEVSSWWRSALLWRAFFTT AVVAVVLRALIEYCRSGRCGLFGKGGLIMFDVSSGVVTYTATDJPPVILLGILGGVLGALYNFFLDKILRLYNVINEKGAPYKJLLAVT VSVVTSCCLFGLPWLAPCKPCP |
| 6 | LPIVVYPALGGLGAGLIALKYPGILYWGFTNVDEILHTGKSASAPGIWLLAQLAAAKVVATALCKGSGLVGGLYAPSLMIGAAVGAVFG GSAAYLIN |
| 7 | PAIKAVQAAITLGTGCSLGPEGPSVDIGKSCALGCAEMMENNRERRIALVAAGSAAGIASGFNAAVAGCFFAIETVLRPLKAENSPPFT TAMI |
| 8 | ASLFFNTNDDAIRNJFSTGTPGEFRJDSLLIFF |
| 9 | EAGAPRREEEGEELGDGAPPEWALLLVGCLLGLATGICVAAFNRGVHVIHDWAWAGTPTEGAAWLRLQRLADTWHRILLIPVSGGVIVG MMHGLLEIFEQLKLAKSSQREGID |
| 10 | AIPGNAAVAHPQAYALVGMAATLASVCSVPLTSVLLLFELTKDYRILLPLMGAVGLAIWVPS |
| 11 | VVDEPPFPGLPELHGLVLRSHLVVLLRKRWFLAEPVKTEE |
| 12 | DEASALISSCLTRGFQYHGNERGLVTCFPDTDLSTAKVLMEVKGIKQLPVVKRGVGHRTDGRRKLLGLLHYDSIGHCLR |
| 13 | LGLFTYGIAVPSGLFIPVILMGAAYGRJVGLVL |
| 14 | ILASVISSTVSNVLLGEKPAFTVPAYELKSAAELPLYLILGMLCGVVSVVFGRLVVW |
| 15 | ERPPVVGILTRHDFMAEHILGLFPHLAKK |
| 16 | SEEMLLDELKVSRAMSKRYIKVTPSATVKEALLLMHDKQQGCVLVVDNEDFLEGJVTVGDIRRRGFESSED |
| 17 | SVVEKVGTVVEVLRNTGHNGF |
| 18 | ASSJDHGLFAVLGAAALLGGS |
| 19 | EDAPEACPTIGRSGNYKKFQCPAGHYNDL |
| 20 | LDLGKEGPLVHIGACLANLLSQGGSGRFR |
| 21 | DSSEDPGSAEDGRSSLERPLL |
| 22 | VRERFSPVELAKKNS |
| 23 | RSDSGDDLELAIEEDGLKHYG |
| 24 | RHLDRRLSARGRARHVDQGP |
| 25 | EEIENWKAIYQRKED |
| 26 | MSTAVGPAGDLPGTTALPGDVAPAVPAVADEHICVDVPRDG |
| 27 | RWKKVRFGKTAFSNF |
| 28 | MQPEVDECLMSHVCGVVRCKIWSVTPQMP |
| 29 | SSSLSCPSPRAAAGHRHV |
| 30 | RFFAYMKERFD |
| 31 | AIYC |
| 32 | FTKTFDLIRKK |
| 33 | NQPNDGESSGF |

Supplementary Table. 6 Naming of incomplete domain CLC genes

| Ensembl ID | Gene Name |
| --- | --- |
| TraesCS2A02G309900 | *TaCLC-c1-2AL* |
| TraesCS2B02G326900 | *TaCLC-c1-2BL* |
| TraesCS2D02G308100 | *TaCLC-c1-2DL* |
| TraesCS3B02G144700 | *TaCLC-c1-3B* |
| TraesCS3D02G254500 | *TaCLC-e-3DL* |
| TraesCS4A02G277600 | *TaCLC-d-4AL* |
| TraesCS4B02G035500 | *TaCLC-d-4BS* |
| TraesCS4D02G033500 | *TaCLC-d-4DS* |
| TraesCS5A02G449500 | *TaCLC-g1-5AL* |
| TraesCS5B02G457100 | *TaCLC-g1-5BL* |
| TraesCS5D02G456000 | *TaCLC-g1-5DL* |
